# Metal Defects in MAPbI_3_ Perovskites: Uncovering the Roles of Ni, Cu, Ag, and Au

**DOI:** 10.1021/acsomega.5c09558

**Published:** 2025-12-05

**Authors:** Lucas G. Chagas, Andreia de Morais, Israel C. Ribeiro, Zeno C. Brandão, Francisco C. Marques, Ramiro M. dos Santos, Juarez L. F. Da Silva, Jilian N. de Freitas, Matheus P. Lima

**Affiliations:** † Department of Physics, Federal University of São Carlos, 13565-905 São Carlos, São Paulo, Brazil; ‡ Center for Information Technology Renato Archer—CTI, 13069-901 Campinas, São Paulo, Brazil; § São Carlos Institute of Chemistry, University of São Paulo, Av. Trabalhador São-Carlense 400, 13560-970 São Carlos, São Paulo, Brazil; ∥ Institute of Physics Gleb Wataghin, University of Campinas, 13083-859 Campinas, São Paulo, Brazil

## Abstract

In perovskite solar cells, understanding how transition metals penetrate the perovskite layer and affect its degradation and optoelectronic properties is crucial for designing more stable devices. Here, we combine experiments and density functional theory to investigate the interaction of Ni, Cu, Ag, and Au with MAPbI_3_. Our simulations show that Au and Ni spontaneously incorporate into MAPbI_3_, as revealed by their negative formation energies. Although Au, Cu, and Ag prefer interstitial configurations, Au behaves distinctly, indicating that the atomic radius plays a more decisive role than the valence configuration. Ni, in turn, preferentially substitutes Pb sites without introducing midgap states despite its partially filled 3d shell. Experimentally, X-ray photoelectron spectroscopy, current–voltage measurements, and UV–vis absorption reveal that all metals diffuse into MAPbI_3_, but only Ag and Cu form semiconductor halide phases that degrade device performance. In contrast, Au and Ni migrate without compromising optical absorption or charge transport, consistent with theoretical predictions. In general, these results highlight Au and Ni as promising contact materials for perovskite solar cells, as their incorporation avoids detrimental electronic or radiative effects compared to other metals.

## Graphical abstract

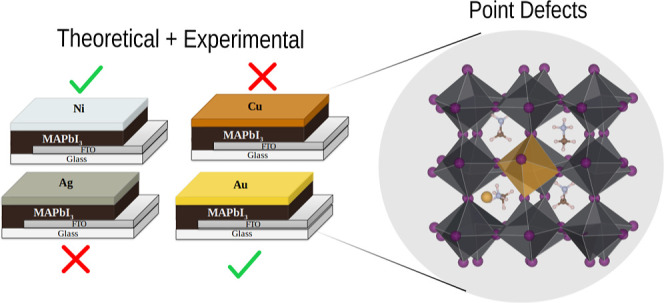

## 1 Introduction

Halide perovskites have rapidly emerged as leading light-absorbing materials for solar cell applications, primarily due to the remarkable rise in their power conversion efficiency (PCE) over the past decade, i.e., PCE increased from 3.8% in 2009 ^1^ to approximately 26.7% in 2024.^2–4^ These materials generally adopt the chemical formula *ABX*_3_, where the *A* site hosts a monovalent cation, *B* is a divalent metal cation, and *X* represents a halide monovalent anion. Hybrid organic–inorganic perovskites, in particular, integrate inorganic frameworks (e.g., A = Cs^+^, B = Pb^2+^, and X = I^–^ or Br^–^) with an organic cation at the A site, such as methylammonium (MA^+^, CH_3_NH_3_^+^).^5^ These hybrid structures combine the advantages of facile and cost-effective synthesis with exceptional optoelectronic performance, exemplified by methylammonium lead iodide (MAPbI_3_).^6^

Among the various halide perovskites, MAPbI_3_ remains the prototypical composition due to its relatively simple crystal structure, excellent optical absorption, and well-characterized intrinsic defect chemistry, making it an ideal model for investigating degradation mechanisms. Incorporation of organic cations MA^+^ contributes to the outstanding efficiency of the material but also introduces structural instability through its dynamic behavior and sensitivity to external stimuli.^7,8^ Continued progress in perovskite solar cell technology therefore depends on elucidating the fundamental origins of these instability pathways and designing strategies that improve long-term stability without compromising performance.^9^

Defects play a central role in the long term stability of halide perovskites. For example, they can act as pathways for ion migration and as nonradiative recombination centers, leading to performance degradation under operating conditions.^10^ Moreover, certain defects promote interfacial reactions with metallic contacts, forming insulating compounds such as AgI or CuI, which further hinder charge transport and accelerate device degradation.^11,12^ These defect-driven processes ultimately induce chemical and structural instabilities that limit the operational lifetime of perovskite-based devices. Such defects may originate during synthesis or be generated by thermally activated ion migration. In this context, point defects predominate and can be classified as intrinsic or extrinsic. Intrinsic defects involve native species of the material, whereas extrinsic defects occur when external atomic species enter the structure.^13^ Different types of imperfections include vacancies, antisites, interstitials, substitutionals, among others.^11,14,15^ Furthermore, the architecture of a perovskite solar cell incorporates various materials, each performing a specific role. For example, the absorber layer (perovskite) was sandwiched between additional layers, such as the electron transport layer (ETL), hole transport layer (HTL), and metallic contacts. Therefore, understanding the interactions between perovskites and the other device components is essential to optimize both the efficiency and stability.

In particular, the presence of metals brings particular features. Recently, metallic grid morphology has been employed as charge collecting electrodes with the advantages of enhancing conductivity and enabling module interconnection,^16^ i.e. allowing the connection of several smaller subcells to form a larger solar cell device.^17^ Beyond the benefits, the interaction between metals and the absorber material can be detrimental, introducing extrinsic defects or combining with intrinsic defects already present in the perovskite structure. It is worth mentioning that even in a situation in which the metallic contacts do not directly interface the perovskite layer, metal atoms can migrate from the contacts to the perovskite layer facilitated by the presence of defects or pinholes in the interfacial layers, and/or by diffusion of mobile species.^18^

Experimental studies reported the use of specific metal species as grids to improve perovskites solar cells. For example, Al metal grids on Indium tin oxide (ITO) substrates result in reduced substrate resistance, thus improving the conductivity of solar cells.^17^ Moreover, Kim et al. employed Au grids deposited over FTO glass,^19^ demonstrating that perovskite solar cells manufactured on this substrate achieved a certified PCE of 12.1%, while devices without these metal grids exhibited a PCE of only 5.5%. The findings include the work of Li et al., who fabricated flexible perovskite solar cells with a PCE of 13.6% using Cu grids on PET substrates as a low-cost solution for industrial applications.^20^ Furthermore, Yang et al. demonstrated in 2021 the fabrication of flexible PET/PDMS substrates containing embedded hexagonal shaped Ag grids with low electrical resistance.^21^ These experiments demonstrate the advantages of metal grids and guide the choice of metallic species for next-generation.

Atomistic simulations help to elucidate the hidden mechanism and design of improved devices, particularly by investigating the interplay between metal adatoms and the perovskite layers through the exploration of metal defects. The most well-known case is that of Au, with previous work by Kerner et al. showing that this metal occupies the interstitial site in MAPbI_3_ with a charge state (*q*) of +1.^10^ Lyons and Swift, studied the substitutional doping of Ag, Na, Cu in MAPbI_3_ at the sites *A* and *B*. Their results indicated that Na and Ag act as shallow acceptor dopants when occupying the Pb site in MAPbI_3_.^22^ Soopy et al., in 2023, tested the insertion of Cu^+^ into substitutional and interstitial sites and concluded that Cu prefers to occupy the interstitial site, reducing the energy barrier by lowering the work function of the perovskite film, which significantly enhances carrier extraction.^23^ The work of Liu et al. suggests that using Ni^2+^ in MAPbI_3_ can access the substitutional site of Pb, passivating Pb vacancies and Pb–I antisite defects to enhance structural stability.^24^ The other studies claim that the metal Ag, Na, Cu, Bi, In, Au is capable of activating different sites, specifically the substitutional and interstitial sites, each with likely different charge states.^22,25^

Relevant applications have stimulated the study of various metals as grid materials for use in electronics and photovoltaic industries, including Ag, Au, Al, Cu, Ni, Mo, Pd, Ta, Pt, among these, Au, Ag, Cu, and Ni are of particular interest due to their extensive use as contacts or grids in perovskite solar cells, each presenting distinct trade-offs between conductivity, stability, and cost, as developed in recent experiments.^26^ Da Silva et al. highlight the use of Au and Ag as contacts in highly efficient lab-scale perovskite solar cells. However, these metals are not suitable for long-term device operation because of the migration of ionic species from the perovskite to the contacts, or the diffusion of the metals into the internal layers of the cell. In particular, interactions involving Ag and Au can degrade perovskite performance, requiring its understanding (or the search for alternative materials) to develop strategies to enhance both the efficiency and operational stability of devices using these metals. Da Silva et al. also proposed Ni as the most suitable choice for industrial applications in perovskite solar cells and minimodules, considering long-term operation. In addition, they suggested that Cu is a highly desirable metal for use in perovskite contacts because of its high electrical conductivity (greater than that of Ni) and low cost, but this metal might not be stable over time. It is also important to note that there have been efforts to replace Ag with Cu even in silicon-based solar cells. Thus, these recent findings encourage further investigation of defects associated with Ni, Cu, Ag, and Au in MAPbI_3_.

Despite significant progress in understanding defect physics in hybrid perovskites, a comprehensive picture of how different metal species interact with the MAPbI_3_ lattice, considering both substitutional and interstitial incorporation as well as their possible charge states, remains incomplete. In this study, we address this knowledge gap by integrating first-principles calculations with experimental validation to systematically compare the behavior of Au, Ag, Cu, and Ni in MAPbI_3_. This combined approach establishes direct correlations between defect energetics, stability, and electronic behavior, providing a unified framework to understand metal-induced effects in hybrid perovskites. Our theoretical model employs a carefully constructed pristine supercell to reproduce realistic features, into which dopant metals were introduced to evaluate their energetic behavior through formation energies (*E*_F_) for various charge states at substitutional and interstitial sites. This analysis reveals that Au, Ag, and Cu preferentially occupy interstitial sites, while Ni replaces Pb atoms in the host MAPbI_3_. Regarding the electronic structure, although Au, Ag, and Cu belong to the same group in the periodic table, Cu and Ag alter the band structure markedly different from Au. Thus, the atomic radius is more important than the electronic shell. Remarkably, incorporation of Ni results in a clean band gap, indicating its potential as a promising metal contact for perovskite-based solar cells with enhanced stability.

On the experimental side, we employ X-ray photoelectron spectroscopy, current–voltage characterization, and UV–vis absorption to evaluate the stability and interfacial behavior of ultrathin Ag, Au, Cu, and Ni contacts on MAPbI_3_ films under accelerated aging. Our results show that although all metals diffuse in the perovskite layer, only Ag and Cu undergo interfacial reactions that severely degrade the electrical response, consistent with the formation of insulating AgI and CuI groups. In contrast, Au and Ni also migrate but preserve both the optical absorption and charge transport properties, consistent with theoretical predictions that their incorporation does not introduce midgap states. These findings underscore the distinct chemical interactions of different metals with MAPbI_3_ and provide guidance for the rational selection of stable contact materials in devices based on perovskite.

## 2 Theoretical and Experimental Techniques

### 2.1 Total Energy Calculations

This work presents theoretical simulations performed based on the DFT framework, as implemented in the Vienna Ab initio simulation package (VASP), version 5.4.4,^27,28^ using the full potential projector augmented-wave (PAW) method to account for interactions between valence and core electrons. The Kohn–Sham (KS) orbitals are expanded in a plane-wave basis set.^29,30^ Structural optimizations were carried out using the semilocal exchange–correlation (XC) functional developed by Perdew, Burke, and Ernzerhof (PBE).^31^ To account for weak van der Waals (vdW) interactions, which is particularly relevant in hybrid organic–inorganic perovskite systems,^70^ we included the D3 dispersion correction proposed by Grimme and co-workers^32^ in all geometry optimizations.

For evaluating energetic properties such as formation energies and electronic properties, a more accurate methodology was employed. Since the conventional DFT-PBE approach is known to suffer from self-interaction errors, leading to underestimated electronic band gaps and inaccuracies in localized electronic states,^33,34^ we adopted the hybrid XC functional proposed by Heyd, Scuseria, and Ernzerhof (HSE),^35^ which incorporates a variable mixing parameter α to tune the fraction of exact Fock exchange.^36^ This α parameter was adjusted to reproduce the experimental band gap of pristine MAPbI_3_ in the structure modeled in this work.^37,38^ Additionally, spin–orbit coupling (SOC) was further included in all electronic and energetic properties to account for relativistic effects, particularly important for heavy elements such as Pb,^39,40^ an approach that we label as HSE + SOC in this work.

The equilibrium volume of the 2 × 2 × 2 MAPbI_3_ supercell was determined under a cubic symmetry constraint (*a*_0_ = *b*_0_ = *c*_0_ and α = β = γ = 90°). Both the stress tensor and the atomic forces were minimized using a plane wave energy cutoff of 620 eV, corresponding to 1.5× ENMAX_max_, where ENMAX_max_ = 413.992 eV denotes the highest recommended cutoff among the constituent atomic species (that is, carbon) as specified in the PAW potential files. The 1.5 scaling factor was used to ensure adequate convergence of the stress tensor components with respect to the size of the set of plane wave bases. For defect-containing structures, the lattice volume was fixed to that of the pristine bulk to isolate the effects of atomic relaxations.

All calculations that did not require the evaluation of the stress tensor were performed using a reduced cutoff energy of 465 eV (see 1.125× ENMAX_max_). The Brillouin zone was sampled using an automated ***k***-point mesh defined by *R*_***k***_ = 30 Å, which corresponds to a 2 × 2 × 2 ***k***-point grid for the 2 × 2 × 2 MAPbI_3_ supercell. For density of states (DOS) calculations at the PBE level, a denser sampling was used at *R*_***k***_ = 60 Å, while HSE + SOC simulations were confined to the Γ-point alone, due to limitations in computational resources. The convergence criterion for the electronic self-consistent field (SCF) cycle was established at 1 × 10^–5^ eV, and structural relaxations were deemed complete once all atomic forces dropped below 0.025 eV/Å.

### 2.2 Structure Models

#### 2.2.1 MAPbI_3_ Bulk Model

Idealized geometry models for perovskites, such as tetragonal, orthorhombic, and cubic structures,^41^ based on repeated structural motifs, were already employed in DFT reported investigations to provide useful physical insights. However, as recently noted, these ideal structures fail to capture local symmetry breaking, which is essential to accurately correlating bond length distributions and electronic structure between theory and experiment.^42^ In practice, such local distortions can be introduced by randomly displacing of halide atoms, followed by full structural relaxation. Another critical feature involves the orientation of the MA^+^ cations. Due to their linear and polar nature, their orientational degrees of freedom significantly affect the structural and electronic properties in simulations. Therefore, constructing supercells with a negligible net electric dipole (achieved by randomly rotating the MA^+^ molecules) is an effective strategy for realistic DFT modeling.^43^ Based on this approach, we constructed geometry models incorporating local asymmetries and random orientations MA^+^, following recent successful studies.^44,45^ Specifically, a cubic 2 × 2 × 2 supercell was used, introducing structural distortions via random displacements of iodine and rotations MA^+^.

Figure 1 illustrates the steps adopted to build the geometry models. We started from a 1 × 1 × 1 cubic unit cell with a lattice parameter of *a*_0_ = 6.28 Å, which was expanded to a 2 × 2 × 2 supercell. In the subsequent step, the eight MA^+^ molecules were randomly rotated using a pseudorandom number generator (PRNG), ensuring a negligible net electric dipole from their combined orientations. Finally, random displacements of the I^–^ ions were introduced by shifting their positions to 0.5 Å in arbitrary directions, also using a PRNG. To evaluate the impact of structural randomness, multiple configurations were generated, each with different I displacements and MA^+^ rotations.

**Figure 1:**
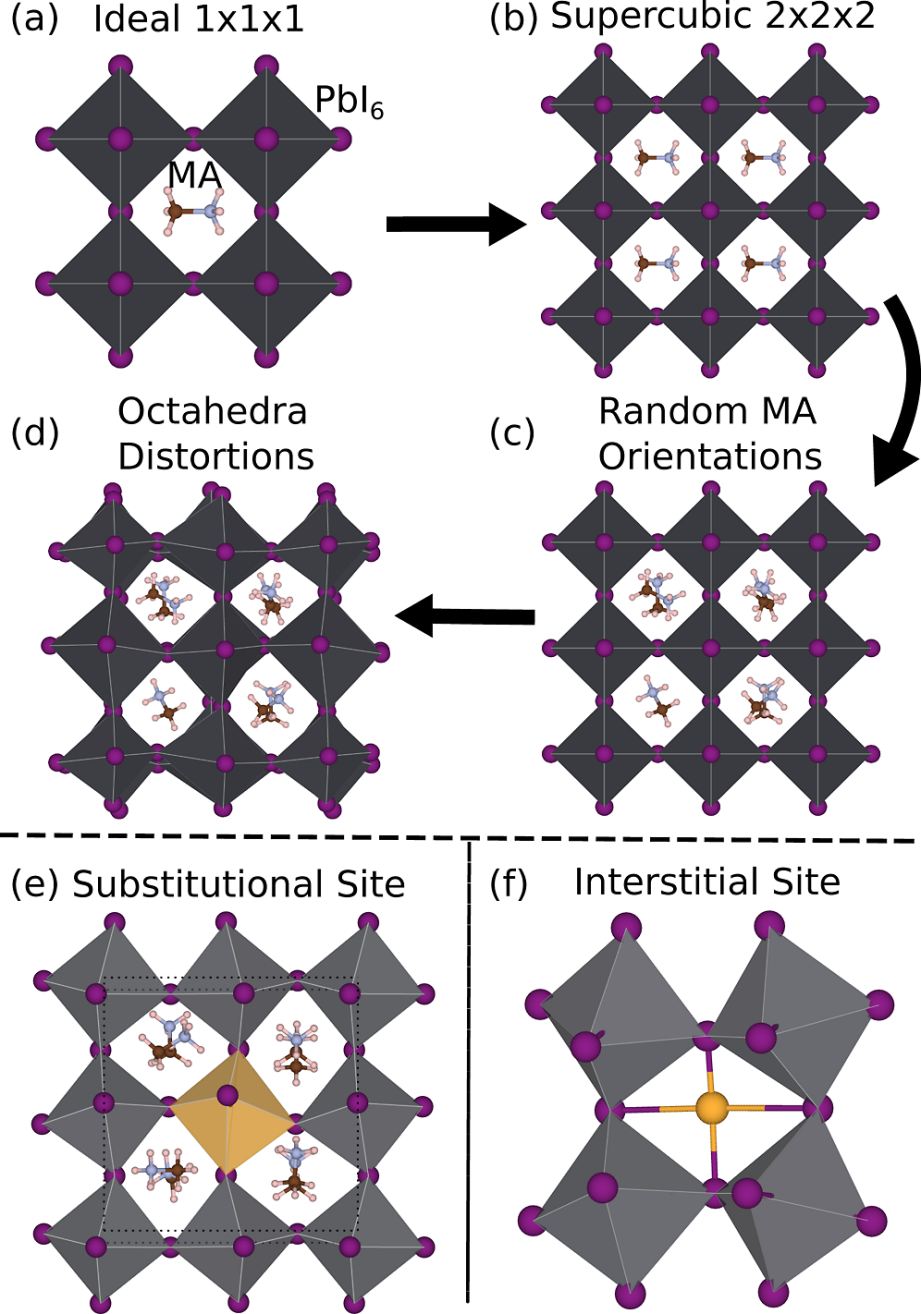
Schematic representation of the procedure used to construct the MAPbI_3_ structure for DFT simulations. (a) Initial 1 × 1 × 1 cubic unit cell. (b) Expanded 2 × 2 × 2 supercell derived from the ideal structure. (c) Random rotations applied to the MA^+^ molecules. (d) Random displacements of iodide atoms. The lower panels show the point defects investigated: (e) substitutional doping, where a Pb^2+^ atom is replaced by a dopant atom; and (f) interstitial doping, in which the dopant metal is inserted between iodine atoms belonging to different octahedra. Purple and yellow spheres represent iodine and dopant atoms, respectively, while the gray octahedron highlights a Pb^2+^.

#### 2.2.2 Selection of Point Defects in MAPbI_3_ from Literature

To guide this study, we performed a screening of point defects involving metals Au, Ag, Cu, and Ni, which are among the most frequently investigated dopants in MAPbI_3_, according to recent literature. This survey includes reports on the formation of these metal-related defects in various charge states (0, −1, +1, +2, and +3), along with discussions of the most energetically favorable doping sites in each case.^10,23,25,46,47^ Substitutional doping is often found at the B site, followed by interstitial incorporation (where the dopant occupies a site between iodine atoms belonging to different BX_6_ octahedra). Although rare, a few studies have explored doping at the A-site; however, its relevance is limited. For instance, in the case of Ag doping, this configuration was explicitly ruled out as energetically unfavorable compared to other sites.^48^ The unfavorable nature of A-site doping may be attributed to the inability of small monovalent metal cations to stabilize the perovskite lattice when placed at this position, especially when their ionic radii deviate significantly from the requirements imposed by the Goldschmidt tolerance factor for forming a stable and photoactive ABX_3_ phase.^49^ Similarly, X-site doping would require halide substitutions by monovalent anions, which is not characteristic of most metal dopants. Therefore, the relevant doping configurations considered in this work are limited to substitutional defects at the B site and interstitial dopants. Additional details can be found in the Supporting Information (SI).

### 2.3 Formation Energy Evaluation

Analyzes of formation energy (*E*_F_) offer a quantitative metric to evaluate the energetic expenditure involved in the creation of particular defects within materials. This facilitates the discernment of the most thermodynamically advantageous configurations. It also accounts for the possible trapping of charge in localized defect states.^50–52^ In this work, *E*_F_ is evaluated using the following equation

1 $$E_{F}\left(TM,q\right)=E_{tot}\left(TM,q\right)-E_{p}-\sum_{i}\mu_{i}n_{i}+q\left(\mu_{E}+E_{VBM}+\Delta E\right)+E_{corr}$$

where *E*_tot_(TM, *q*) denotes the total energy of the MAPbI_3_ supercell doped with a transition metal (TM) atom, in charge state *q* (in units of *e*). *E*_p_ is the total energy of the pristine (undoped and neutral) system. Each atomic species added to or removed from the system during defect formation is indexed by *i* = Pb, Au, Ag, Cu, Ni, where *n*_*i*_ represents the number of atoms added (*n*_*i*_ > 0) or removed (*n*_*i*_ < 0). The corresponding values μ_*i*_ are the chemical elemental potentials of species *i*, calculated as the total energy per atom of their most stable bulk elemental phase. The term μ_E_ is the electronic chemical potential, which is varied from 0 to the band gap value. *E*_VBM_ is the valence band maximum (VBM) eigenvalue obtained from the pristine supercell, and Δ*E* is a correction term used to align the VBM values of the pristine and doped systems. Finally, *E*_corr_ accounts for the spurious interactions between periodic images of the charged defect in supercell calculations. In the following, we discuss in detail the evaluation of Δ*E* and *E*_corr_.

#### 2.3.1 Potential Alignment—Δ*E*

The expression of the formation energy (eq 1) requires the maximum valence band energy (*E*_VBM_), ideally taken from the pristine (undoped) system. However, when defects are modeled within the supercell approach, as in this work, a mismatch arises between the reference eigenvalues of the doped and undoped systems,^51^ necessitating a correction to *E*_VBM_. We estimated this correction, Δ*E*, as the difference between the core-level eigenvalues of the doped and undoped systems, specifically the 1*s* eigenvalues of the Pb atoms, calculated using the initial-state approach in VASP.^53^ Since these core levels are influenced by the distance between the Pb atoms and the defect site, and because we consider two different defect types that prevent the use of a common reference atom in all cases, Δ*E* was determined as the average of the 1*s* eigenvalues of the Pb atoms located farthest from the dopant metal atom.

#### 2.3.2 Correction for Charged Defects—*E*_corr_

The supercell approach also introduces spurious errors when extra charges are added to the simulation cell.^51^ In particular, the electrostatic interaction between a charged defect and its periodic images causes the electrostatic potential to diverge, which can be solved with the addition of a compensating background charge within the jellium approximation^50^ to neutralize the excess charge. This approximation was done ordinarily by neglecting the zero-order Fourier transform term of the charge density in the Hartree potential evaluation.^54^ In such a neutralized system, the total energy converges slowly with increasing supercell size, due to the residual interaction between the localized charged defect and the uniform jellium background. To address this, Makov and Payne^50^ proposed a correction term for eq 1, which depends on the linear supercell dimension *L* = *V*_SC_, where *V*_SC_ is the supercell volume

2 $$E_{corr}=\frac{q^{2}\alpha_{M}}{2\epsilon L}+\frac{2\pi qQ_{r}}{3\epsilon L^{3}}$$

where, α_M_ = 2.837 is the Madelung constant for the cubic cell, ϵ = 22 a.u. is the static dielectric constant of the host (or MAPbI_3_ pristine) as suggested by Wilson et al.,^55^ and *Q*_r_ is the second radial moment of the electron density difference ${\mathrm{\rho ̃}}_{D,q}\left(r\right)=\rho_{D,q}\left(r\right)-\rho_{H}\left(r\right)$ , with ρ_D,q_(***r***) corresponds to the system with a defect *D* in charge state *q*, and ρ_H_(***r***) refers to the pristine host

3 $$Q_{r}=\int_{V_{SC}}d^{3}r{\mathrm{\rho ̃}}_{D,q}\left(r\right)r^{2}$$

However, in the work of Lany and Zunger,^52^ they verified that there is a linear dependence between the second and first terms in eq 2, i.e.

4 $$\frac{2\pi qQ_{r}}{3\epsilon L^{3}}=f\frac{q^{2}\alpha_{M}}{2\epsilon L}$$

where, *f* is a linear coefficient. Thus, by applying this linear dependence, eq 2 can be rewritten as

5 $$E_{corr}=\left(1+f\right)\frac{q^{2}\alpha_{M}}{2\epsilon L}$$

Lany and Zunger also reported that the linear coefficient *f* is −0.35 for cubic supercells. The resulting expression for *E*_corr_, as proposed by these authors, provides improved agreement between theoretical predictions and experimental observations. This approach has been used successfully in numerous studies^10,56^ for charge correction in periodic systems with charged defects. Accordingly, the formation energies reported in our simulations incorporate this correction to ensure accurate results.

### 2.4 Accelerated Aging of Perovskite–Metal Interfaces

All experimental procedures, including metal deposition, XPS analysis, and *I*–*V* measurements, were conducted under an inert atmosphere to avoid exposure to moisture and oxygen. This ensured that the observed variations arose exclusively from metal diffusion and aging effects. The following subsections summarizes the key aspects of the experimental methodology used to obtain our data.

#### 2.4.1 Prepared Samples

A set of experiments was carried out to qualitatively support the theoretical results presented here. For that, we investigated samples containing direct contact between the perovskite layer and the metal layers, as prepared (called “fresh” samples) and after inducing accelerated degradation (called “aged” samples). In this paper, accelerated degradation was induced by heating the samples. More specifically, the samples were kept inside a glovebox (free of moisture and oxygen air to mimic an encapsulated device and avoid the effects of external contaminants), then heated to 85 °C for 24 or 72 h, which would roughly correspond to 6 months and 12 months of solar cell operation, respectively, according to tests used in the Silicon photovoltaic industry. Thus, a batch of 12 samples was analyzed, composed of FTO/MAPbI_3_/metal stacks containing Ag, Au, Cu, or Ni as metallic top contact, both in the fresh and “aged” forms.

#### 2.4.2 Spectroscopic Characterization

X-ray photoemission spectroscopy (XPS) data were collected to analyze whether there were variations in the relative percentage of metal present in the sample surface, comparing the “fresh” and “aged” samples for each given metal. For this analysis, the thickness of the top metallic layer was kept around 4 nm, to allow the collection of XPS signal from both the metal and the sublayer of perovskite, to ensure that the signal from the entire depth of the metal layer (at least in fresh samples) was collected. The methodology followed standard surface-sensitive analysis procedures, ensuring that the photoemission signal reflected compositional variations arising from diffusion or interfacial reactions. Details of sample preparation are provided in the Supporting Information and the results are shown in Figure 6.

#### 2.4.3 *I*–*V* Curve Characterization

In addition, investigate whether the diffusion/mixing of metal atoms with perovskite components would change the optical and electrical properties of these systems; current–voltage (*I*–*V*) curves were obtained for systems prepared with the same stack layers (FTO/MAPbI_3_/metal) as used for XPS but prepared with a thicker metallic layer (∼80 nm) to ensure a functional electrical contact for collecting the *I*–*V* profile of “fresh” and “aged” samples. These measurements were performed under dark, controlled conditions, and the resulting *I*–*V* curves were used to evaluate changes in charge transport and interface resistance between fresh and aged samples.

## 3 Results and Discussion

To gain a deeper understanding of the interactions between metals Ni, Cu, Ag, Au and the host material MAPbI_3_, we performed a series of simulations addressing structural, energetic and electronic properties. For each metal, both substitutional and interstitial defects in multiple charge states were investigated. Our analysis began with the characterization of pristine MAPbI_3_ to ensure a reliable crystalline structure model, taking into account the challenges associated with the simulating of organic–inorganic perovskites. We then evaluated the formation energies (*E*_F_) to identify the most thermodynamically favorable configurations. This was followed by electronic characterization through DOS and band structure calculations for the systems with the lowest *E*_F_. Additional band gap corrections were applied to the cases in which the dopants generated relevant electronic effects. Finally, we compared our theoretical results with our own generated experimental data to provide physical insights. Although only the key findings are presented in the main text, additional details are provided in the Supporting Information.

### 3.1 Pristine MAPbI_3_ Study

First, we examined the undoped host material to ensure a realistic structural model, even when employing a cubic 2 × 2 × 2 supercell. Ten distinct structures were generated by randomly rotating the MA^+^ cations and slightly displacing the iodine atoms, using different seeds for the PRNG. These structures are labeled Cubic w-DisRot *k*, with *k* ranging from 01 to 10. The first row of Table 1 presents the lowest-energy configuration (*k* = 01), while the second row reports the Boltzmann-weighted average over all ten configurations at 300 K. The close agreement between these two sets of values in all properties listed in the table confirms the robustness of the model. The largest discrepancy, approximately 2°, occurs for ${\mathrm{\theta ̅}}_{Pb-I-Pb}$ , which reflects minimal variations in octahedral tilting caused by the different MA^+^ orientations and random iodine displacements. The MA^+^ rotations and random displacements of iodine atoms are both essential to stabilize the system.^43^ Table 1 reports the relative energy (Δ*E*_tot_), using a configuration with unrotated molecules and undisplaced iodine atoms as a zero-energy reference. Introducing both MA^+^ rotations and iodine displacements results in a significant decrease in Δ*E*_tot_, with energy differences of up to −128 meV/f.u. The Boltzmann-weighted average yields Δ*E*_tot_ = −110 meV/f.u., which can be attributed to the energetic stabilization induced by MA^+^ distortions and I^–^ displacements. Additional details are provided in the Supporting Information.

**Table 1:** Table With the Lattice Parameters (*a*_0_, *b*_0_, *c*_0_) in Å, Average Pb–I Bond Lengths in Å, Average Effective Coordination Number of Lead (ECN_av_^Pb^) in NNN, and Average Pb–I–Pb Bond Angles $\left({\mathrm{\theta ̅}}_{Pb-I-Pb}\right)$ in °^a^

structure	*a*_0_	*b*_0_	*c*_0_	*d*_av_^Pb–I^	ECN_av_^Pb^	${\mathrm{\theta ̅}}_{Pb-I-Pb}$	Δ*E*_tot_	*E*_g_
cubic w-DisRot 01	12.55	12.55	12.55	3.22	5.96	155.59	–128	1.54
cubic w-DisRot av	12.56	12.56	12.56	3.22	5.95	157.57	–110	1.53
orthorhombic^57^	8.55	9.18	12.58	–	–	–	–	1.60
**orthorhombic** ^37^	8.83	8.56	12.58	–	–	–	–	1.51
**tetragonal** ^38^	8.85	8.85	12.64	–	–	–	–	1.52
**orthorhombic** ^58^	8.87	8.58	12.63	3.19	–	154.50	–	–
**tetragonal** ^58^	8.81	8.81	12.71	3.18	–	165.30	–	–
**cubic** ^58^	6.32	6.32	6.32	3.16	–	180.00	–	–

a The table also includes the relative total energy per formula unit (Δ*E*_tot_) in meV/f.u., where Δ*E*_tot_ = *E*_xx_ – *E*_Cubic w/o‑DisRot_. The bandgap (*E*_g_) in eV for the Cubic w-DisRot 01 structure was calculated using HSE 33% + SOC. For the average structure, the Boltzmann average of all random structures was calculated using the environmental temperature of 300 K. The bold characters indicate the experimental results.

Table 1 also includes a comparison with theoretical and experimental data from the literature. Although the absolute values of *a*_0_ and *b*_0_ differ among some structures due to variations in their unit cells, *c*_0_ can be directly compared (except for the experimental cubic phase, for which 2*c*_0_ should be considered). In this case, the deviations are smaller than 1.3%. Furthermore, renormalized values of *a*_0_ and *b*_0_ should also be compared by mapping them onto a cubic unit cell, since orthorhombic and tetragonal cells correspond to supercells $\sqrt{2}\times \sqrt{2}\times 2$ . Consequently, we rescaled *a*_0_ and *b*_0_ for Cubic w-DisRot 01 by $1/\sqrt{2}$ , obtaining values of 8.99 and 8.87 Å, respectively. These values are in excellent agreement with those reported in the literature, differing only by 0.04 and 0.02 Å from experimental refs 37 and 38 Therefore, the lattice parameters for the Cubic w-DisRot model 01 are in excellent agreement with the experimental data and will be used for defect investigations in the following sections.

It is important to note that the simulated cubic structures 1 × 1 × 1 should not be directly comparable to the experimental data. The last row of Table 1 reports experimentally measured values for the cubic phase, where the straight Pb–I–Pb bond angle ${\mathrm{\theta ̅}}_{Pb-I-Pb}=180^{{}^{\circ}}$ is not reproduced by DFT simulations for 1 × 1 × 1 unit cells due to the linear morphology of the MA^+^ cations. Experimental measurements correspond to thermal averages, which yield idealized angles and distances inconsistent with simulations at 0 K. Moreover, experiments demonstrated phase transition triggered by the temperature. At low temperatures, the orthorhombic phase occurs below 165 K, when a transition to the tetragonal phase occurs, which is stable up to (327 K). The cubic phase occurs above 352 K.^58^ Here, it is worth mentioning that the variation in lattice parameters across these different temperature-triggered experimentally measured phases (orthorhombic, tetragonal, and cubic) is smaller than the variation observed among our ten Cubic w-DisRot *k* model structures. This highlights a limitation of our supercell approach, which cannot capture these temperature-induced phase transitions.

Our structures also maintain coherence of local structural parameters with values reported in the literature, as summarized in Table 1. The average Pb–I bond length (*d*_av_^Pb–I^) obtained for the lowest-energy configuration agrees with the Boltzmann-weighted average and is approximately 1.2% longer than the experimental values for the orthorhombic and tetragonal phases, a deviation expected for the GGA-PBE approach. In contrast, the average effective coordination number of Pb (ECN_av_^Pb^) differs by only 0.01 NNN between the two models (no reference values were found in the literature), indicating that the PbI_6_ octahedra undergo minimal distortion despite mutual tilting. This behavior, characteristic of Pb-containing systems, does not suggest Jahn–Teller distortions, in contrast to the pronounced distortions observed in the tin-based perovskite CsSnI_3_.^59^ Furthermore, the observed angle variations suggest a compact packing of the octahedra, in which a larger lattice parameter is compensated by octahedral inclination, thereby reducing both the effective lattice constant and the accessible cell volume.

The band gap predicted by the PBE functional is well-known to be underestimated due to self-interaction errors and the absence of relativistic spin–orbit coupling effects.^33,34^ Therefore, we evaluated the total energies used to predict formation energies and band gaps by including SOC and employing the hybrid HSE exchange–correlation functional. We adjusted the mixing parameter for the exact-exchange term to achieve agreement with the experimental band gap, *E*_g_. The commonly used value of α = 1/4, derived from perturbation theory^60^ (i.e., HSE06), does not reproduce the well-established experimental band gap of 1.51 eV. To determine a more accurate value, we performed a linear interpolation between the PBE + SOC and HSE06 (α = 1/4) results and found α = 0.33 to be optimal. We refer to this level of theory as HSE33%+SOC. Our simulated band gaps are listed in Table 1. Zhang et al. employed a similar procedure and obtained α = 45%.^57^ We attribute this discrepancy to differences in the structural model: our study uses a 2 × 2 × 2 cubic supercell with iodine displacements, whereas Zhang et al. used an orthorhombic cell without iodine displacements. The local motif symmetry affects the band gap by altering the hopping interactions between I and Pb sites, an effect also reported in perovskites by Dias et al.^44^

Our analysis shows that the Cubic w-DisRot 01 structure has the lowest total energy among the ten configurations, reproduces the experimental band gap at the HSE33% + SOC level, and exhibits lattice parameters and local symmetry descriptors consistent with experimental data. Consequently, this structure was selected for further investigation of substitutional and interstitial metal doping in all subsequent sections of this work. The next step is to evaluate the formation energies (*E*_F_) for these doped systems.

### 3.2 Formation Energies of Point Defects

Previous studies from our research group^61^ investigated surface and interfacial defect processes in MAPbI_3_. The results reported herein extend this understanding to the bulk region, incorporating the additional consideration of charged defect states. Using eq 1, we calculated *E*_F_ for two types of Ni, Cu, Ag, and Au impurities: (i) substitutional and (ii) interstitial point defects, as shown in panels (e,f) of Figure 1. The corresponding results are presented in Figure 2. The slopes of the curves correspond to charged systems, whereas the horizontal lines represent neutral systems. μ_E_ was varied from 0 eV, corresponding to the valence band maximum, up to 1.54 eV, corresponding to the conduction band minimum; in other words, μ_E_ varies within the band gap as determined from the HSE33% + SOC calculations. These specific curves were selected from the lower *E*_F_ charge states for all metals, and our complete analysis can be appreciated in the Supporting Information. Moreover, only interstitial Au favors a charged state (*q* = +1), while all the other metals have a neutral lowest energy configuration.

**Figure 2:**
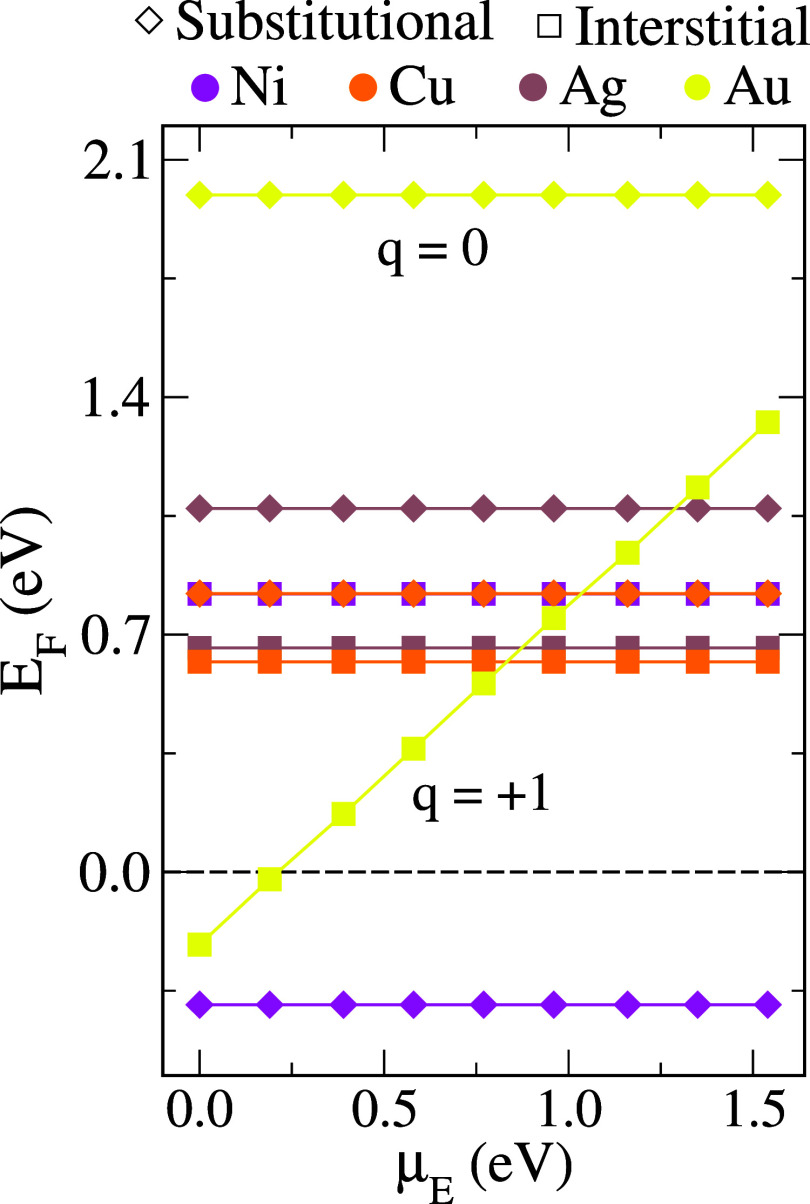
Formation energy (*E*_F_) as a function of electrostatic potential (μ_E_) is shown for substitutional and interstitial doping of all metals, where only the configurations with the lowest *E*_F_ are investigated. Other cases are provided in the Supporting Information. The horizontal lines represent systems with a charge state of 0, while the sloping line represents the system with a charge state of +1. The values were calculated using HSE33% + SOC at the gamma point.

To validate our calculations, we compared them with literature data. Kerner et al.^10^ investigated Au incorporation into MAPbI_3_, the same host material considered in this work, and reported that Au preferentially occupies interstitial sites in the +1 charge state for electronic chemical potentials close to the valence-band maximum. Our simulations reproduce this qualitative behavior for *E*_F_, with only minor quantitative deviations arising from differences in the structural models. Both studies consistently show that within a specific range of μ_E_, *E*_F_ becomes exothermic, indicating that metal incorporation into the perovskite lattice is thermodynamically favored over metal cluster formation. The small differences in absolute *E*_F_ values between our results and those of Kerner et al. can be attributed to two factors: (i) the smaller size of our supercell, which enhances Au–Au interactions and yields slightly higher *E*_F_; and (ii) local distortions present in our structural model, which alter the interaction between Au and the host lattice. These distortions also influence the choice of the HSE mixing parameter α. While Kerner et al. reported an optimal value of α = 0.43, we obtained α = 0.33. Despite these modest quantitative differences, the overall agreement is excellent, particularly considering the complex chemical interactions inherent to hybrid organic–inorganic halide perovskites.^62^

Contrasting the dopants with one another, it is noteworthy that only interstitial Au stabilizes in a charged state, namely *q* = +1, whereas all other metals adopt a neutral configuration (*q* = 0) as their lowest-energy state. Our simulations further predict that Ni is the only dopant species that favors substitutional incorporation rather than interstitial. More importantly, substitutional Ni exhibits an exothermic *E*_F_ across the entire range of μ_E_, suggesting that Ni in contact with MAPbI_3_ tends to diffuse into and incorporate at the *B* site, in contrast to the other metals considered in this study. For substitutional Au, we find that its formation energy is significantly higher than that of the interstitial site, indicating that Au incorporation is unfavorable at substitutional positions but energetically favorable at interstitial sites. Similar analyses were performed for Ag and Cu, though in these cases the energy differences between interstitial and substitutional incorporation are less than 0.4 eV, with a lower energy interstitial doping. Both of these dopants exhibit positive *E*_F_ values over the full μ_E_ range, indicating that their incorporation is endothermic and that the system is more likely to remain phase-separated into individual bulk components. Interestingly, Ag and Cu differ from Au, which strongly favors interstitial incorporation. In fact, for Au, a region of μ_E_ near the valence-band maximum yields exothermic *E*_F_ values, suggesting that under certain conditions Au can spontaneously migrate into the MAPbI_3_ lattice. This result for Au is in excellent agreement with the findings of Kerner et al.^10^

These findings provide important insights into the role of metal dopants in modulating the electronic structure of perovskites for solar cell applications. While Au and Ni can spontaneously diffuse into the perovskite lattice, their effects differ markedly: Ni incorporation contributes to lattice stabilization, whereas Au tends to induce local structural distortions. In contrast, the unfavorable incorporation of Ag and Cu accounts for their experimentally observed degradation pathways through interfacial compound formation. Overall, the correlation between defect energetics and electronic structure modulation elucidates how metal diffusion and defect formation directly influence the long-term stability and durability of perovskite-based devices.

### 3.3 Electronic Properties

#### 3.3.1 PBE + SOC Density of States

To elucidate the impact of metals on electronic properties, we evaluated the DOS and band structures, shown in Figures 3 and 4, respectively. In this analysis, we considered only the configurations with the lowest *E*_F_ (Ni substitutional and Cu, Ag, Au interstitial), as these systems are the most likely to occur.

**Figure 3:**
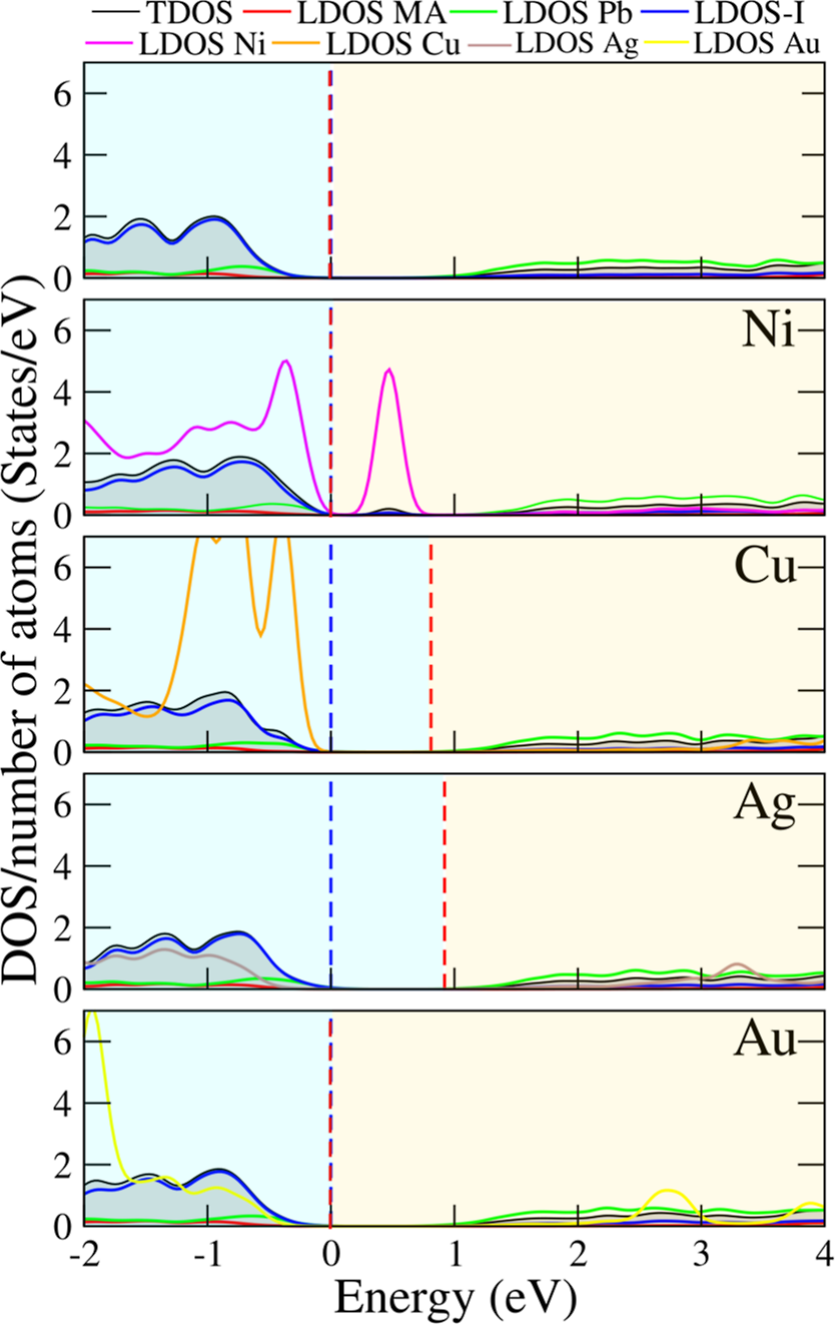
Density of states per number of atoms (DOS/number of atoms) for the undoped and metal-doped systems, corresponding to the configuration with the lowest *E*_F_, is shown. The dashed red line represents the Fermi level, and the dashed blue line indicates the valence band maximum (VBM), set as the energy zero. The regions in blue and yellow correspond to the valence band (VB) and conduction band (CB), respectively. All calculations were performed using the PBE + SOC functional.

**Figure 4:**
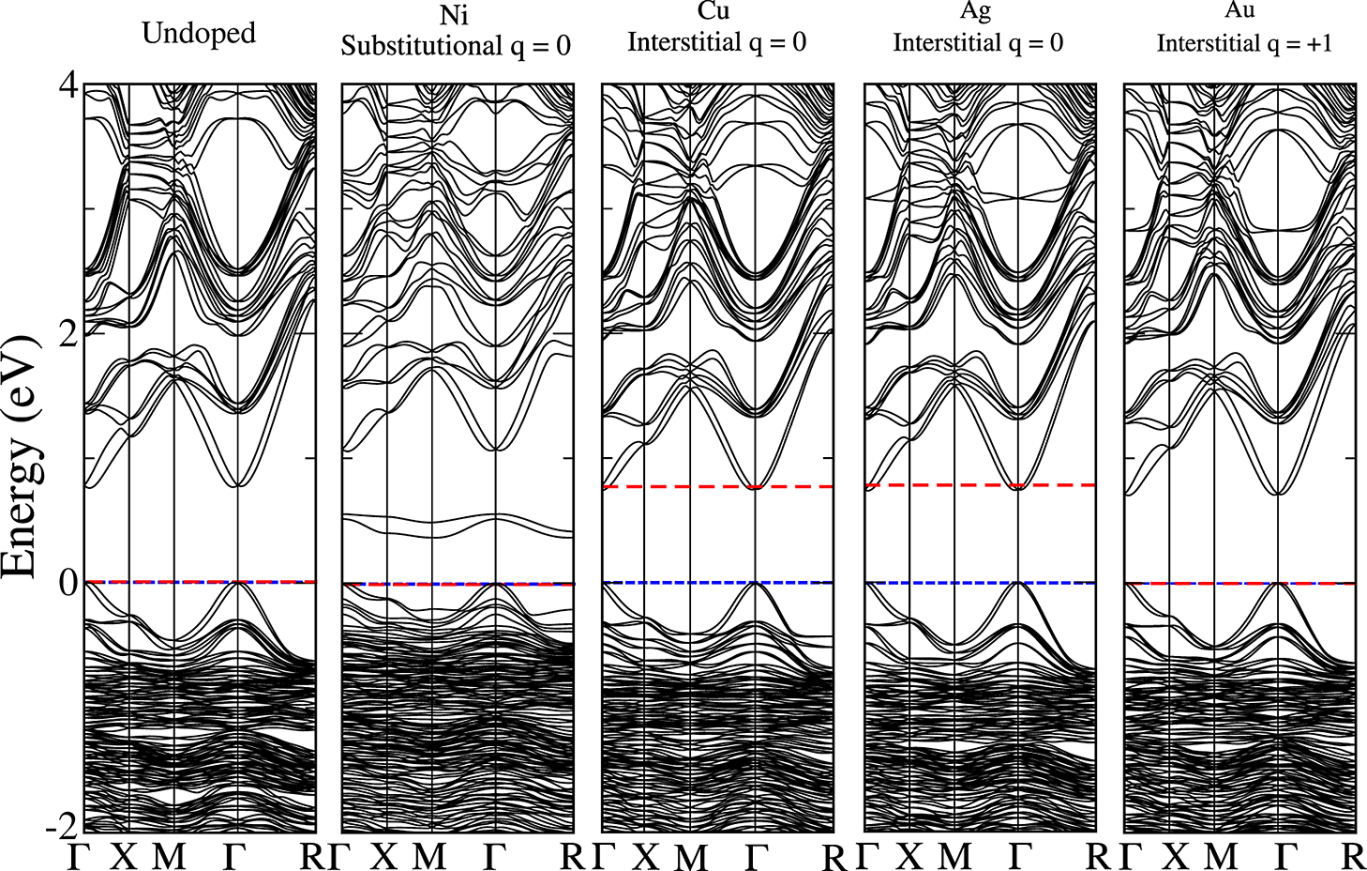
Electronic band structures for MAPbI_3_ perovskite undoped and doped with metals calculations with PBE + SOC. Only the configurations with the lowest *E*_F_ for each metal are plotted. The blue dashed line are the zero of system and the red dashed line indicates the Fermi level, where states below are occupied and those above are unoccupied.

In Figure 3, we present the density of states (DOS) normalized by the number of atoms, evaluated at the PBE + SOC level. We first focus on the pristine MAPbI_3_ (top panel), which clearly exhibits a semiconducting electronic structure with a well-defined band gap. The valence band is dominated by I states, whereas the contributions from the MA cation appear several electronvolts below the Fermi level and are therefore not visible in this panel, but are provided in the Supporting Information. The conduction band is mainly made up of Pb^2+^ and I^–^ states. This electronic structure is consistent with the general picture of hybrid halide perovskites, in which I^–^ and Pb^2+^ atoms govern the optical properties through their dominant contributions at the band edges, while the MA^+^ cation contributes primarily to deeper energy levels. Our results for pristine MAPbI_3_ are in good agreement with previous literature reports.^63^ The band gap obtained at the PBE + SOC level is 0.71 eV. Although this value underestimates the experimental band gap, it is consistent with theoretical calculations at the same level of theory that explicitly account for spin–orbit coupling.^64^ The strong SOC effect arises from the degeneracy of the Pb^2+^ states near the valence band, which are split under SOC, thus reducing the band gap by approximately 1 eV.

Upon metal doping, the DOS shows distinct features near the band gap region depending on the dopant species. The most significant modifications occur in the valence band and within the forbidden gap, while changes in the conduction band are comparatively minor. In particular, substitutional Ni introduces 3d states below the valence band and localized states within the band gap, consistent with its partially filled 3d orbitals. Both Cu and Ag display another behavior: additional states emerge inside the valence band, near the edge. In particular, Cu contributes states at the top of the valence band, indicating Cu-d/I-p hybridization, whereas Ag introduces weaker states located deeper in the valence band, with the most significant contribution below −2 eV. In contrast, Au doping leads to defect states just below the valence band, along with additional contributions around −3 eV below the valence band maximum, indicating the presence of deeper defect levels. The defect-induced states within the band gap become progressively deeper in order Cu → Ag → Au, which can be attributed to their increasing atomic radii and the consequent weaker orbital overlap (reduced hopping) with neighboring I sites.

A central feature of these dopants is the interaction between (i) the preservation or loss of the semiconducting behavior, (ii) the Fermi-level position, and (iii) the preferred defect charge state *q*. Although substitutional Ni introduces localized states within the band gap at the PBE + SOC level, the Fermi level remains within the gap, preserving semiconducting behavior. This suggests that Ni effectively replaces the two electrons originally provided by Pb^2+^. Similarly, Au doping maintains semiconducting behavior, but for a different reason: it prefers an interstitial configuration and stabilizes in a positively charged state (*q* = +1). In this case, the single electron provided by Au is compensated by the defect charging, preserving the semiconducting behavior. In contrast, Cu^+^ and Ag^+^ favor neutral interstitial configurations, thus introducing an extra electron into the system. This shifts the Fermi level into the conduction band, which is not expected in the experimental case because of the exothermic formation energies of these defects observed in the theoretical data presented here.

#### 3.3.2 PBE + SOC Band Structure

This section presents the analysis of the electronic band structure at the PBE + SOC level, with the main objective of further examining the Fermi-level position, band splittings, and dispersion features resolved in crystalline momentum (***k***) space (see Figure 4). In agreement with the DOS and previous reports,^63^ the band structure of the pristine system exhibits semiconducting behavior. The Pb-derived states give rise to a pronounced SOC effect, evidenced by band splittings in the conduction band. As expected for a semiconductor, the Fermi level aligns with the valence-band maximum. Furthermore, a direct band gap appears at the Γ point as a consequence of band folding in the 1 × 1 × 1 unit cell, which corresponds to a direct gap at the *R* high-symmetry point in the primitive Brillouin zone. In the following, we analyze how the metal doping changes this picture.

The Ni-doped system shows the Fermi level aligned with the zero-energy reference (i.e., the valence-band maximum), indicating a fully occupied valence band and an empty conduction band, but with localized states appearing inside the band gap. This behavior originates from the strong hybridization between Ni–3d and I–5p orbitals, which produces localized states within the band gap, consistent with previous reports.^24,47^ When the hybrid functional is employed, these states shift toward the valence band, reducing their midgap character, as discussed in the next section. Such defect states are unfavorable for photovoltaic applications, as they can act as recombination centers, facilitating nonradiative decay pathways that reduce device efficiency.^65^ In contrast, the behavior of Cu and Ag is similar to each other but distinct from the other metals: both introduce additional electronic occupation at the bottom of the conduction band, as revealed in the DOS and more clearly in the band structure. In this case, doping donates extra electrons to the host, leading to *n*-type conductivity. The fraction of conduction band filled depends on dopant concentration; in our simulations, one dopant per eight MAPbI_3_ formula units corresponds to a doping level of 12.5%. A different scenario is observed for Au: the preferred interstitial configuration with charge state *q* = +1 results in a fully filled valence band and an empty conduction band, without states in the band gap. Thus, among the investigated dopants, Au more effectively preserves the intrinsic semiconducting character of the host, a behavior that is not detrimental to carrier transport.

In the specific case of Ni, the emergence of states within the band gap may be partly attributed to the limitations of the exchange–correlation functional, as the semilocal PBE + SOC approach suffers from self-interaction errors and typically underestimates band gaps. To address this issue, we subsequently employed the HSE33% + SOC hybrid functional to re-evaluate the electronic structure of the most promising dopants, namely the Ni- and Au-doped systems.

#### 3.3.3 Band Gap Corrections

The PBE + SOC approach used in the previous sections is useful for unveiling features such as projected density of states and ***k***-resolved band splittings. However, it cannot provide reliable band gap values or accurate positions of localized states. It is worth emphasizing that all our formation energy calculations were performed within the HSE33% + SOC hybrid functional framework, while the DOS and band structures discussed earlier relied on PBE + SOC, owing to the prohibitive computational cost associated with including empty states and dense ***k***-meshes. Since Ni introduces defect states inside the band gap, a more accurate assessment of its electronic structure is mandatory. To this end, we carried out additional HSE33% + SOC calculations, restricted to the Γ-point, which, together with the PBE + SOC DOS and band structures, provides a consistent and accurate picture.

The results are summarized in Figure 5. In this analysis, we focus exclusively on Au and Ni, as these dopants exhibit the lowest *E*_F_ values, suggesting spontaneous incorporation in the perovskite layer. In contrast, the instabilities observed for Cu and Ag arise from charge counting effects,^66^ not from the misplacement of defect levels, and therefore cannot be resolved by using a more accurate functional. For Au, the HSE33% + SOC electronic structure reveals a significant band gap widening, but no new states appear within the gap, as expected. Interestingly, the resulting band gap even exceeds that of pristine MAPbI_3_, while maintaining the semiconducting character. This effect is likely a consequence of the relatively high concentration of dopants used in our simulations (12.5%), which may lead to an overestimation of the gap. In the case of Ni, the defect states that previously appeared within the band gap at the PBE + SOC level are shifted into the conduction band, specifically around 2.3 eV above the valence band maximum. As with Au, the HSE33% + SOC approach also increases the band gap, bringing it close to the pristine value, thereby restoring a clean semiconducting gap for substitutional Ni. This result confirms that Ni adopts a +2 oxidation state, consistent with the substitution of Pb^2+^ at the *B*-site, and demonstrates that Ni largely preserves the intrinsic electronic properties of pristine MAPbI_3_.

**Figure 5:**
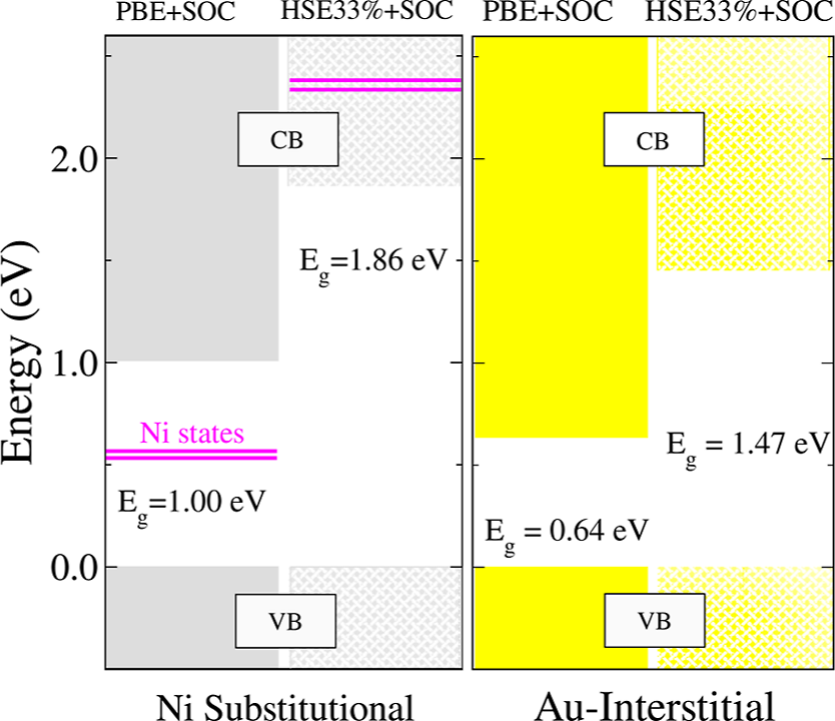
Energy diagram for the systems with the lowest *E*_F_. These calculations were performed using PBE + SOC and HSE33% + SOC at the Γ-point only, to achieve higher precision and examine the band gap states of the metals in MAPbI_3_. The values were shifted to zero, using the Au and Ni results obtained with PBE + SOC as the reference.

These theoretical results provide direct insight into how metal incorporation affects the photovoltaic performance of perovskite devices. The absence of midgap states for Au and Ni indicates that these metals do not introduce nonradiative recombination centers, thereby preserving carrier lifetimes and sustaining high open-circuit voltage; both essential for achieving elevated PCE. In contrast, Cu and Ag introduce defect-related states near the band edges, which can act as shallow traps or nonradiative recombination sites, ultimately hindering charge transport and promoting efficiency losses. Overall, the predicted electronic structures suggest that devices employing Au and Ni contacts are likely to exhibit enhanced efficiency and operational stability compared with those using Cu or Ag, in agreement with the experimental trends discussed below.

### 3.4 Insights from Experimental Data

Figure 6 displays the relative amount of metal measured in each sample, estimated from XPS data. These values consider all metallic species present in the sample, regardless of their oxidation states (for example, it was not differentiated between Au^0^ and Au^+^). Therefore, even if chemical reactions between the metal and perovskite components occur (i.e., formation of AgI and CuI, which may easily happen in this type of interface),^12,67^ the metallic element would still be considered and quantified in the relative amount calculation. The values were calculated considering the contents of O, N, Pb, and I atoms in the sample probed volume.

**Figure 6:**
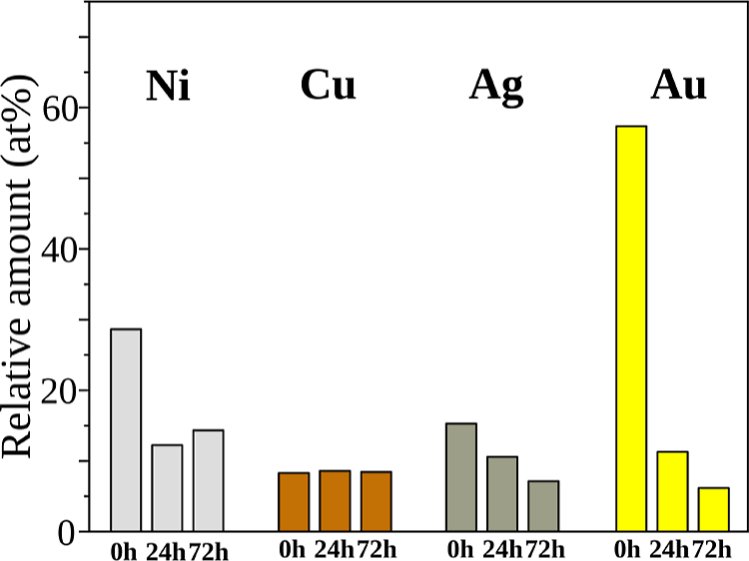
Relative amount (in at %) of metal observed in the surface of the sample by XPS measurements. Samples consisted of FTO/MAPbI_3_/metal stacks, where the metal layer was composed of ∼4 nm of Ag, Au, Cu, or Ni. Measurements were collected for “fresh” samples (0 h) and “aged” samples (heated at 85 °C for 24 or 72 h).

It is important to note that the measured signal from elastic photoelectrons, which contributes to quantification, comes from a depth of up to 15 nm from the surface while being exponentially biased toward the surface, since the signal corresponding to elastic photoelectrons is attenuated differently with depth for each metal through the perovskite (Figure S-23 from Supporting Information),^68^ as is the signal from the perovskite layer itself, also attenuated differently between metals. This is one of the causes for the distinct (at %) in the fresh sample between the different metals. Au, for example, has the highest relative concentration at 0 h because the large attenuation (small Inelastic Mean Free Path—IMFP) of the perovskite layer signal across the Au layer causes photoelectrons coming from Au itself to become the most significant part of the measured signal. In addition, the smaller amount of metal present in fresh samples may also be related to a coalescence effect of metallic particles in thin films deposited on the perovskite surface (an effect that has been clearly observed for Ag and Cu by FEG-SEM analysis in a previous work),^26^ which would expose a larger volume of the perovskite film to the XPS reading.

Interestingly, for the series measured here, only the samples prepared with Cu did not show variations in relative amount after accelerated aging. Significant decreases were observed for aged samples containing the other metals, with the reduction more intense in the following order: Au > Ni > Ag. A decreased amount of metal measured on the surface of the sample is most likely related to the diffusion/migration of metal atoms to the bulk of the MAPbI_3_ film or the diffusion/migration of perovskite components to the surface. The loss of metal through the formation of volatile components is not likely to occur. Therefore, the most likely effect responsible for the variations observed in relative amounts of Au, Ni, and Ag is the diffusion/migration of metal species to the majority of the perovskite layer. When metal atoms diffuse from the electrode into the perovskite layer, they may chemically react with the perovskite components, irreversibly modifying its composition, or act as dopants that introduce trap states within the band gap. Both effects can alter the optical absorption and charge-transport properties of the active layer, thereby reducing device performance. Because metal migration is often accelerated by prolonged operation or thermal stress, these degradation mechanisms tend to become more pronounced over time. It is also important to note that different metals will induce distinct effects depending on their electronic configuration, electronegativity, and atomic radius.

It is worth mentioning that other authors have previously reported the diffusion of Ag and Au into the perovskite layer.^67,69^ Here, we propose that Ni is also prone to diffuse, causing a significant variation of the amount of metal exposed on the surface (as seen in Figure 6), in agreement with the favorable interaction (spontaneous substitutional doping by Ni) discussed in theoretical modeling. Although not quantifiable in absolute values, the reduction in the atomic percent (at %) of a given metal due to diffusion into the perovskite layer should proceed in the same order as the signal attenuation for a given metal, in the order of Cu > Ni > Ag > Au (Figure S-23 from Supporting Information, inset). That is, relative to being measured as a top layer Cu would suffer the most reduction in the total at % if it diffused a give (measurable) depth into the perovskite. On the other hand, Au would suffer the least reduction in the at %. As seen in Figure 6, the reduction with aging being more intense in the order Au > Ni > Ag > Cu indicates that the depth to which the metal penetrates also differs between them even if considering the coalescence effects, which were not observed for Au and Ni.

From the current–voltage curves displayed in Figure 7, it can be seen that only minor changes in the electrical properties of the systems containing Au or Ni contacts; whereas significant loss of conductivity was observed in the samples containing Ag or Cu contacts following accelerated aging. A reduction in conductivity after aging indicates an increase in series resistance, leading to fewer charge carriers being efficiently extracted by the electrodes. This effect directly lowers the short-circuit current density (*J*_sc_) and the fill factor (FF), thereby reducing the overall power conversion efficiency (PCE) of the device. From these results, we draw two conclusions: (i) even if there is diffusion of Au and Ni to most of the perovskite layer, the interaction between these metals and the perovskite components does not appear to significantly affect the electronic properties of the system. This is in agreement with the theoretical findings that doping with Au or Ni would introduce no intermediary states in the band gap (see the HSE33% + SOC results, i.e., the corrected electronic structure in Figure 5), so it would not cause a loss in the photovoltaic performance of the cells. (ii) In samples containing Ag and Cu, there is some sort of change that leads to a significant decrease in conductivity. According to the literature, the interaction between Ag and Cu with perovskite leads to the formation of AgI and CuI, respectively,^12,67^ which could passivate the electrical contacts, thus causing a significant loss of conductivity. These results reinforce the close correlation between the predicted defect energetics and the experimentally observed degradation behavior.

**Figure 7:**
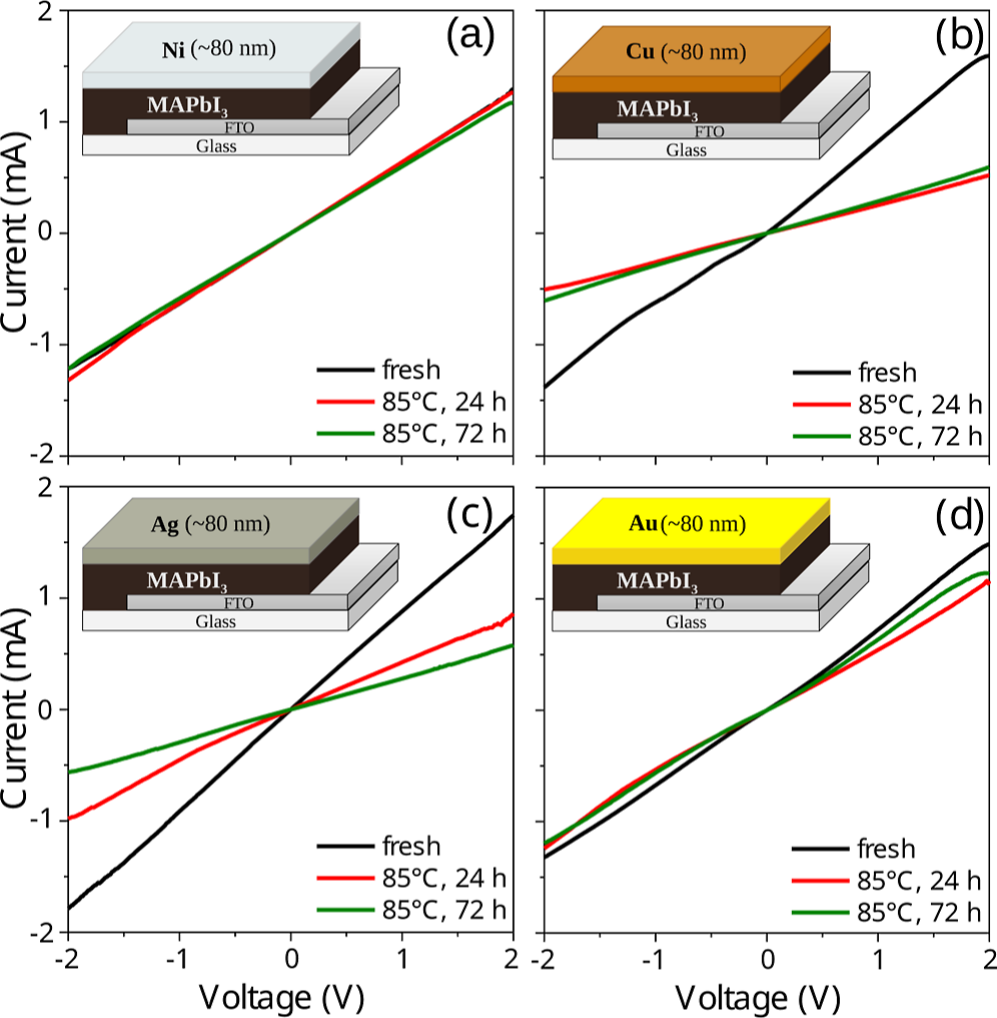
Current–voltage curves of FTO/MAPbI_3_/metal systems containing the four different metals, (a) Ni, (b) Cu, (c) Ag, and (d) Au, for “fresh” and “aged” samples.

Finally, the optical properties of the “fresh” and “aged” samples were evaluated by collecting UV–vis absorption spectra of the FTO/MAPbI_3_/metal stacks. For this experiment, the metal layer used had ∼4 nm of thickness (as used for the XPS analysis) to ensure that the optical response of the perovskite layer could be measured. The data collected from this analysis is shown in Figure S-24 (see the Supporting Information). No changes in the region related to band gap absorption were observed for any of the samples of MAPbI_3_, suggesting that the interactions of Au, Ag, Cu, and Ni with MAPbI_3_ do not introduce intermediary electronic states in the band gap of this perovskite, in agreement with the theoretical data presented here.

In summary, comparing experimental evidence with theoretical modeling, we suggest that although there is a preferred interaction between MAPbI_3_ and Au and Ni, which may cause a facilitated diffusion of species, this movement would not cause significant damage to the optical and electronic properties of those samples. To provide an overview of the observed trends, Table 2 summarizes the migration tendencies of the investigated metals (Ag, Au, Cu, and Ni) and their corresponding effects on the electronic structure and stability of MAPbI_3_.

**Table 2:** Summary of Metal Behavior and Effects on MAPbI_3_

property	Au	Ag	Cu	Ni
site	interstitial	interstitial	interstitial	substitutional
*E*_F_	exothermic	endothermic	endothermic	exothermic
midgap states	no	yes	yes	no^a^
effect (aged)	stable	drops	drops	stable

a In-gap states are observed only at the PBE + SOC level.

## 4 Conclusions

To summarize our results, we found that the metals Au and Ni have negative formation energy, i.e., spontaneously diffuse into the perovskite MAPbI_3_, however, they do not add localized states close to the band gap region. The Au metal is more likely to activate the interstitial site, which aligns with previous findings in the literature, while Ni tends to occupy the substitutional site. Furthermore, metals from the same column of the periodic table exhibit different behaviors in terms of state occupancy. Specifically, Au does not introduce states into the band gap, whereas Ag and Cu contribute to the occupancy near the bottom of the CBM. For Ni, our results indicate a high probability of substitutional incorporation, where the metal states are not located in the middle of the band gap but instead reside approximately 0.3 eV above the conduction band. This region is not detrimental to solar applications, corroborated by experimental results, suggesting that Au and Ni can diffuse into MAPbI_3_ without affecting its electronic properties.

These findings suggest that the use of Au and Ni in perovskite solar cells is promising. When incorporated into MAPbI_3_, these metals do not cause significant harmful effects. This work advances our understanding of the inevitable interactions between perovskites and metals, providing clearer guidance for future applications in solar cell technology, particularly those that may occur in long-term operating devices.

## Supplementary Material



## Data Availability

As mentioned, all DFT calculations were done using the Vienna textitAb initio Simulation Package (VASP) package, which can be used under a nonfree academic license. Additional details can be obtained from the link, https://www.vasp.at/. Furthermore, additional details are provided within the electronic Supporting Information, while additional input and output of all data can be obtained under the URL https://data.mendeley.com/ and all additional crude data can be obtained directly with the authors under request.

## Notes

The data employed for the figures, as well as complementary analyses and additional technical details, are reported within the Electronic Supporting Information (PDF)

## Abbreviations

DFT: density functional theory
PBE: Perdew–Burke–Ernzerhof
PVK: perovskites
VASP: vienna ab initio simulation package
HSE: Heyd–Scuseria–Ernzerhof
SOC: spin–orbit coupling
FEG-SEM: field emission gun-scanning electron microscope
FTO: fluorine-doped tin oxide
XPS: X-ray photoelectron spectroscopy
