# Supplementary material for: Metal Defects in MAPbI3 Perovskites: Uncovering the Roles of Ni, Cu, Ag, and Au
Source: ACS Omega. 2025 Dec 5;10(49):60886–99. doi: 10.1021/acsomega.5c09558 (PMC12713506; doi:10.1021/acsomega.5c09558)
Supplement: Supplementary file 1 [file ao5c09558_si_001.pdf]

Supporting Information

Metal Defects in $MAPbI_3$ Perovskites: Uncovering the Roles of Ni, Cu, Ag, and Au

Lucas G.Chagas,[†] Andreia de Morais,[‡] Israel C. Ribeiro,[¶] Zeno C. Brandão,[§] Francisco C. Marques,[§] Ramiro M. dos Santos,[¶] Juarez L. F. Da Silva,[¶] Jilian N. de Freitas,[‡] and Matheus P. Lima[*,†]

†*Department of Physics, Federal University of São Carlos,* 13565-905*, São Carlos, SP, Brazil*

‡*Center for Information Technology Renato Archer – CTI,* 13069-901*, Campinas, SP, Brazil*

¶*São Carlos Institute of Chemistry, University of São Paulo, Av. Trabalhador São-Carlense* 400*,* 13560-970*, São Carlos, SP, Brazil*

§*Institute of Physics Gleb Wataghin, University of Campinas,* 13083-859*, Campinas, SP, Brazil*

E-mail: mplima@df.ufscar.br

Contents

S-1 Introduction **S-2**

S-2 Review of the Literature **S-3**

S-2.1 Pristine $MAPbI_3$ S-3

S-2.2 Point Defects in $MAPbI_3$ S-3

S-3 Additional Computational Technical Details **S-7**

S-3.1 Selected PAW Projectors S-7

S-3.2 Input File: INCAR S-7
S-3.3 Generation of the $MAPbI_3$ Structures S-9
S-3.4 Computational Tests: Contrasting 2×2×2 and 4×4×4 Supercell Sizes. . . S-11

S-4 Additional Results for the Pristine $MAPbI_3$ Compound S-12

S-4.1 Structural Properties S-13
S-4.2 Total and Local Density of States S-22
S-4.3 Electronic Band Structures S-24

S-5 Additional Results: Tests for Au Point Defects in $MAPbI_3$ S-27

S-5.1 Tests for Dopants at Different Substitutional Sites S-28
S-5.2 Tests for Dopants at Different Interstitial Sites S-29
S-5.3 Au Doping at Substitutional B and Interstitial Sites S-30

S-6 Additional Results for Points Defects in $MAPbI_3$ S-32

S-6.1 Additional Formation Energy Data S-33
S-6.2 Additional Electronic Data S-34

S-7 Additional Details: Experimental Procedure S-43

References S-48

S-1 INTRODUCTION

The electronic supporting information file contains complementary theoretical and experimental data, figures, tables, and discussions. Specifically, (i) a review of the literature, (ii) input files, projector selection, and a description of the generation of random supercells, (iii) additional data on doping tests with the Au atom, and (iv) additional data on the structural, energy, and electronic analysis of point defects, (v) a description of the experimental procedure, detailing the preparation of the substrate and the $MAPbI_3$ film; (vi) the deposition process; (vii) XPS analysis; ($viii$) I–V measurements; and (ix) UV–vis absorption spectroscopy.

S-2 REVIEW OF THE LITERATURE

S-2.1 Pristine $MAPbI_3$

Perovskite-derived materials have shown significant promise for use in solar cells, especially after groundbreaking research by Kojima *et al.* in 2009.[1] This material typically follows a general formula ABX_3, where site A contains an organic or inorganic cation, site B is a metallic ion, and site X is a halide.[2] In the case of the perovskite $MAPbI_3$ (MA = $CH_3NH_3^+$), which incorporates both organic and inorganic components, recent advancements have led to energy conversion efficiencies reaching approximately 25 %, a value close to that of silicon solar cells, which is about 26 %.[3] The $MAPbI_3$ has been well-studied, and its properties are widely described in the literature. For example, it undergoes two phase transitions: one from orthorhombic to tetragonal at T = 161 K (−112 °C) and the second from tetragonal to cubic at T = 327 K (54 °C).[4] To summarize this information from the literature, we have compiled the known $MAPbI_3$ parameters in Table S-1.

Table S-1. Different geometrical parameters and band gaps for $MAPbI_3$ have been reported in the literature. This table summarizes lattice parameters (a_0, b_0, c_0), volumes per formula unit (V_0/f.u.), band gaps (E_g), references (Ref.), and methodologies used in each study.

Phase	Supercell Models	a_0 (Å)	b_0 (Å)	c_0 (Å)	V_0/f.u. (Å^3)	E_g (eV)	Ref.	Method
Orthorhombic	$\sqrt{2}\times\sqrt{2}\times 2$	8.55	9.18	12.58	246.85	1.60	[5]	Theory.
Orthorhombic	$\sqrt{2}\times\sqrt{2}\times 2$	8.56	8.84	12.58	237.75	1.51	[6]	Exp.
Tetragonal	$\sqrt{2}\times\sqrt{2}\times 2$	9.02	9.02	12.88	261.77	1.67	[7]	Theory.
Tetragonal	$\sqrt{2}\times\sqrt{2}\times 2$	8.89	8.89	12.07	238.48	1.60	[8]	Theory.
Tetragonal	$\sqrt{2}\times\sqrt{2}\times 2$	8.85	8.85	12.64	247.48	1.52	[9]	Exp.

S-2.2 Point Defects in $MAPbI_3$

However, this material, $MAPbI_3$, suffer from structural instability (due to, for example, chemical reaction with H_2O, and release gases).[10,11] One of the causes of the instability problem is defects in the material, defects are imperfections in the crystalline structure of the material that occur during synthesis and influence its properties, such as MA,

Pb and I (V_{MA}, V_{Pb}, V_I) vacancies and interstitial (MA^{int}, Pb^{int}, I^{int}) defects, where the iodine vacancies are considered to be one the main source of defects in $MAPbI_3$.[12,13]

Another class of defects that can occur is doping defects, which involve the insertion of a foreign atom or ion into the lattice site of an atom in the material. The literature shows that MA^{int} and Pb^{int} are unable to introduce states within the band gap of the perovskite. This lack of capability can affect the electronic and optical properties of the material, thereby impacting its solar applicability. On the other hand, the I^{int} defects are related how to common defects, with small energy formation, and affect the material with the introduction of the deep states in the band gap;[5] this causes non-radiative recombination, i.e. the loss of energy in the form of heat. The iodine vacancy, V_I, is reported to be an electron donor and therefore more stable in the positive charge state, V_I^{1+}. The V_{MA} vacancy is found as an electron acceptor near the valence band maximum (VBM), designated as V_{MA}^{1-}; however, the formation energy calculations indicate a low concentration of this defect. In the case of the lead vacancy, V_{Pb}, studies show this defect as a charge acceptor in the (0,−2) states. The charged defect V_{Pb}^{2-} is considered one of the most stable in $MAPbI_3$.[14]

When dealing with the use of perovskite in photovoltaic devices, it is essential to consider the interaction between the perovskite and the other components of the photovoltaic cell. Metals are used as charge-collecting materials in the top electrode, and also in the bottom electrode when integrating perovskite as a photovoltaic material over large areas. This approach is widely used because the transparent substrate typically employed for assembling perovskite cells has low conductivity, leading to significant energy losses over large areas. The metallic grids improve conductivity and connect several smaller modules to form a larger cell. Different metals have been tested as grid materials, including Ag, Au, Al, Cu, Ni, and Pt.[15]

The choice of metal varies according to factors such as cost, conductivity, and ease of deposition, which are influenced by the technique used. Among the wide range of metals available, we selected nickel (Ni), copper (Cu), silver (Ag) and gold (Au). The Au is a noble metal commonly used in perovskite photovoltaic devices due to its

high conductivity and excellent resistance to oxidation and corrosion when exposed to environmental conditions. However, its cost is relatively high for applications that require significant quantities. Ag is also frequently used because it has a lower cost and higher conductivity than gold. However, silver is highly susceptible to oxidation in the environment, and although it is more affordable than Au, it is still more expensive than metals such as Ni and Cu. On the other hand, Cu has excellent conductivity, surpassing that of Au and Ni, second only to Ag in this respect. It is also cheaper than Au and Ag, although it is highly prone to oxidation. Ni, while less expensive than Ag and Au, has good environmental stability but lower conductivity compared to Au, Ag, and Cu. Thus, each metal has advantages and disadvantages.

The decision to use a particular metal depends on balancing these factors according to the specific needs of the project, such as cost, stability, conductivity, and manufacturing method. In table S-2, we present a literature review of the most important defect studies, focusing on tests with the metals Au, Ag, Ni, and Cu. We searched for studies investigating the use of these metals with different charge states, including 0, −1, +1, +2, and +3, and which sites are more favorable to doping, such as preference for sites B and interstitial sites (I_{Metal}). Site A can also be doped, but its occurrence in the literature is limited. The only example of doping at site A found was with Ag, but this work dismissed this dopant at site A, as it was shown to be unfavorable.[16] This is particularly associated with the fact that monovalent metal cations cannot occupy site A in the ABX_3 perovskite structure, as they are too small and mismatched for the lattice to sustain a photoactive $ABX3$ with an appropriate Goldschmidt tolerance factor.[17] Additionally, site X requires monovalent ions, and these metals, being monovalent cations, cannot occupy this site. Based on this information, we will use it as an initial guide for our work.

Table S-2. Literature review on metal (Au, Ag, Cu, Ni) doping in $MAPbI_3$ from the most recent studies. The columns indicate the dopant, type of defect, doping site (A, B, X), interstitial doping (Y^{int}), charge state, methodology used in the work, relevant information for comparison with our study, and references.

Dopant	Doping	Charge State	Study Method	Information	Reference
Au	A	-	-	-	-
Au	B	+3	teo./exp.	DOS	[18]
Au	B	0	teo./exp.	DOS	[19]
Au	X	-	-	-	-
Au	Au^{int}	0, +1	teo./exp.	E_F	[8]
Ag	A	0	teo./exp.	E_F	[20]
Ag	B	+1	teo./exp.	Internal Parameters	[16]
Ag	B	−1	teo./exp.	E_F	[20]
Ag	X	-	-	-	-
Ag	Ag^{int}	+1	teo./exp.	E_F	[8]
Cu	A	-	-	-	-
Cu	B	0	teo./exp.	Internal Parameters	[16]
Cu	B	+2	teo./exp.	E_F	[21]
Cu	X	-	-	-	-
Cu	Cu^{int}	0	teo./exp.	E_F	[21]
Ni	A	-	-	-	-
Ni	B	0	teo./exp.	DOS and Bands	[22]
Ni	B	+2	teo./exp.	DOS	[23]
Ni	X	-	-	-	-
Ni	Ni^{int}	-	-	-	-

S-3 ADDITIONAL COMPUTATIONAL TECHNICAL DETAILS

S-3.1 Selected PAW Projectors

Table S-3. Selected PAW projectors. All POTCAR files were obtained from the potpaw_pbe_5.4 library. The table presents the most important information for the selected PAW projectors, including the PAW-PBE projector name (POTCAR), date of projector creation (Date), number of valence electrons (Z_{val}), electron configuration of valence states (Valence), and maximum recommended cutoff energy (ENMAX) for each element.

Element	POTCAR	Date	Z_{val}	Valence	ENMAX (eV)
H	H_GW	04/21/2008	1	$1s^1$	300.000
C	C_GW	09/28/2005	4	$2s^2 2p^2$	413.992
N	N_s_GW	04/04/2007	5	$2s^2 2p^3$	296.495
I	I_GW	03/12/2012	7	$5s^2 5p^5$	175.647
Ni	Ni_GW	03/31/2010	10	$3d^9 4s^1$	357.323
Cu	Cu_GW	05/19/2006	11	$3d^{10} 4s^1$	417.039
Ag	Ag_GW	03/06/2008	11	$4d^{10} 5s^1$	249.844
Au	Au_GW	03/23/2010	11	$5d^{10} 6s^1$	248.344
Pb	Pb_d_GW	04/14/2014	16	$5s^2 5d^{10} 6s^2 6p^2$	237.809

Table S-3 presents the selected PAW projectors. The POTCAR files for all atomic species include the GW variant. All optimized geometries were obtained using stress tensor calculations with a force tolerance criterion of 0.025 eV/Å. The chosen cutoff energy defining the plane-wave basis size (ENCUT) for these calculations is 1.5× the highest value of ENMAX among all elements (i.e., for C, ENCUT = 413.992 eV). However, the remaining properties use a lower value of ENCUT (517.490 eV). We follow this protocol due to the slower convergence of the stress tensor with ENCUT compared to the electronic properties.

S-3.2 Input File: INCAR

```
SYSTEM = MAPbI3 (bulk)


ENCUT = 620.998 !  Cutoff energy for plane waves basis set in eV
ALGO = Normal !  A Davidson blocked iteration scheme
NELMIN = 6 !  Minimum number of eletronic SCF steps (default 2)
```

NELM = 200 ! Maximum number of electronic SC steps (default 60)

NELMDL = -12 ! Number of NON-selfconsistency steps

EDIFF = 1.0E-5 ! Global-break condition for the electronic SC-loop (ELM)

AMIX = 0.1000 ! Charge mixing parameter (default 0.8)

BMIX = 0.00010 ! Charge mixing parameter (default 1.0)

LMAXMIX = 6

PREC = Normal ! Calculation level (Changes FFT-grids)

ISPIN = 1 ! Spin-polarized calculations (default)

ADDGRID = .TRUE. ! PAW flag: additional grid

LASPH = .TRUE. ! Non-spherical contributions from the gradient corrections inside the PAW spheres

LREAL = Auto ! Evaluation of projection operators: reciprocal space

IVDW = 11 ! Grimme D3 vdW correction

NSW = 5 ! Number of ionic steps : default =0

EDIFFG = -0.025 ! Stop if all forces are smaller than |EDIFFG| (default EDIFF X 10)

IBRION = 2 ! Conjugated-gradient algorithm used to relax ions

ISIF = 3 ! Force, stress, relax ions, change cell shape, change cell volume (default = 0)

POTIM = 0.50 ! Parameter for the structure optimization algorithm (default)

ISMEAR = 0 ! Gaussian smearing (default 1)

SIGMA = 0.01 ! Width of smearing (default 0.2)

LORBIT = 10 ! DOSCAR and PROCAR written, quick projection scheme, without providing the Wigner-Seitz radius

```
NWRITE = 1 !  Determines how much information will be written in OUTCAR
LWAVE = .FALSE. !  WAVECAR not written
LCHARG = .FALSE. !  CHG and CHGCAR not written

NCORE = 5 !  cores per orbital (default 1)
LPLANE = .TRUE.
```

S-3.3 Generation of the $MAPbI_3$ Structures

Although the literature indicates that the tetragonal phase of $MAPbI_3$ is commonly used for DFT calculations and is also observed at ambient temperatures, there exists a discrepancy between the theoretical and experimental descriptions in some cases. In a paper by Zhao *et al.*, the authors discuss the deviations from experimental values and advocate the use of polymorph structures, specifically the cubic supercell, for a more accurate description.[5] These polymorphous networks exhibit significantly lower predicted total energies, larger band gaps, and dielectric constants dominated by ionic contributions. The authors' study suggests that experimental data based on cubic parameters cannot be adequately described by monomorphic cells, such as orthorhombic and tetragonal cells, which are inherently monomorphic. Although it is possible to construct a tetragonal supercell, specifically with dimensions $\sqrt{2}\times\sqrt{2}\times 2$ (not monomorphic), this structure can only account for symmetry breaking, not temperature effects.

The latter can be described by random displacements of I atoms, which requires a larger number of atoms to simulate statistically random displacements of the I atoms. Our structures do not exhibit broken symmetry without disrupting the periodicity of the system. However, the cubic supercell (polymorph) allows for the representation of various local low-symmetry motifs, including tilting, rotations, and *B*-atom displacements. This theoretical cell simulation enables for a more accurate description of the experimental behavior of the perovskite. It accommodates the

assumption that the experimental data represent average values, which can be better simulated using the cubic supercell due to its ability to model rotations, tilting, and displacements within the cell. For these reasons, in this work we will use the cubic supercell with dimensions $2 \times 2 \times 2$.

One relevant question, when referring to the configurations of the material, is whether there are more free configurations for the MA cations in the center of the perovskite site A. This can result in a greater number of possible configurations and allows changes in the positions of halides. To assess the impact of these positional configurations, we performed calculations with different structures, each featuring random displacements of the I atoms and rotations of the MA molecule. The schematic generation of the structure is shown in Figure S-1. First, we initialize the ideal cubic cell $1 \times 1 \times 1$; second, we build the cubic supercell 2×2×2; and third, we generate random rotations of the molecule MA and random displacements of the atoms I. All structures were optimized using stress tensor calculations, and the results of these calculations are shown in Table S-6.

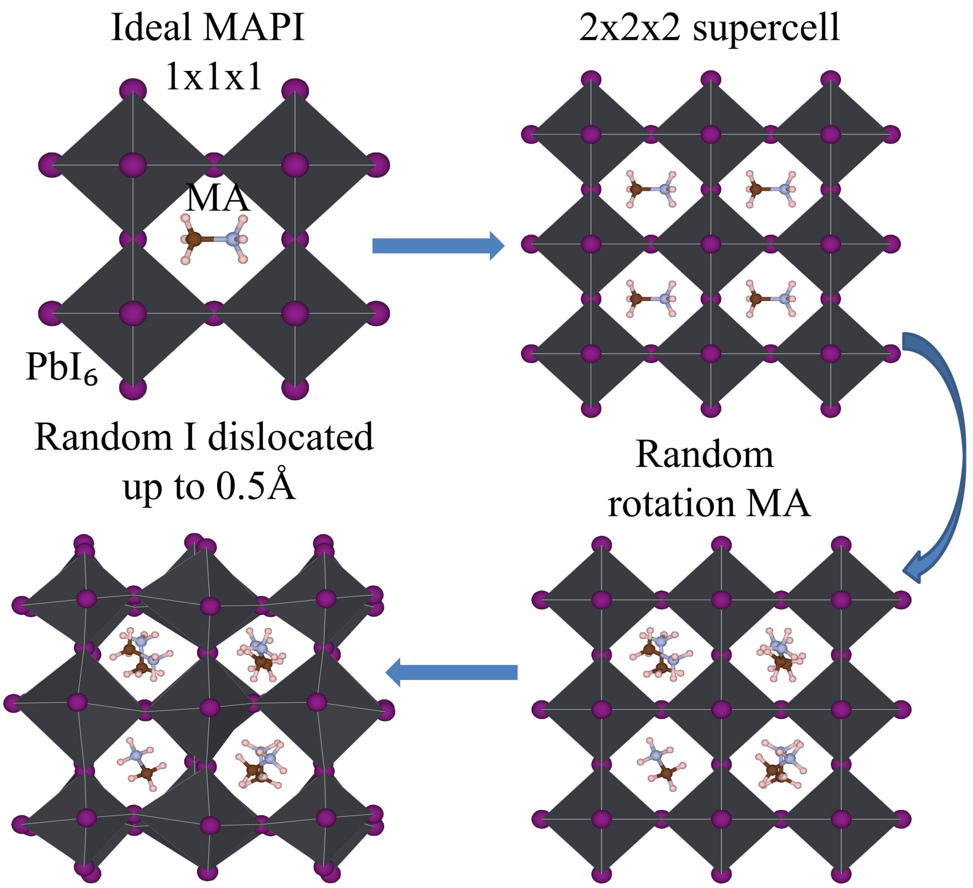

Figure S-1. Schematic representation of the construction of the $MAPbI_3$ structure used for DFT calculations. The sequence involves four steps: (i) starting from a $MAPbI_2$ 1×1×1 cubic unit cell with lattice parameters extracted from the literature; (ii) expanding this unit cell to a 2×2×2 supercell; (iii) applying random rotations to each MA molecule and selecting a final structure with a null electric dipole inside the unit cell; and (iv) introducing random displacements of the iodine atoms to break symmetry and mimic temperature effects. The final structure was optimized before extracting physical properties.

S-3.4 Computational Tests: Contrasting 2×2×2 and 4×4×4 Supercell Sizes.

We conducted tests using the 4×4×4 supercell, which represents larger structures compared to the 2×2×2 supercell. This complicates the calculations, as these larger structures entail additional computational costs. To assess the feasibility of calculations for any system that exhibits special behavior, we performed tests using the 4×4×4 supercell, which was generated for the lowest energy structure (1). Initially, our tests focused on run-time and memory coast. The results of these initial tests are presented in Table S-4. We conducted three calculations using different configurations, all employing only the gamma point and single point calculations. Our findings indicate that the

80 ppn configuration is preferable for future calculations, as it offers lower time and memory values compared to other configurations.

Table S-4. Estimated computational costs for two configurations: a 4×4×4 supercell with 786 atoms (H = 384, C = 64, N = 64, Pb = 64, I = 192) generated from the lowest-energy I Dist_Rot 01 structure, and a modified configuration with additional displacements of Pb and I atoms up to 0.2 Å (Pb and I Dist). Reported values include processes per node (ppn), number of cores (NCORE), elapsed time, and RAM usage for three test cases.

$2 \times 2 \times 2$ from I Dist_Rot 01	ppn	NCORE	Time (s)	Memory (kb)	Time (hour)
Undistorted	20	5	21 113.86	3 629 816	5.87
	40	5	15 493.78	3 137 612	4.30
	80	8	5178.62	3 076 772	1.44
With Pb and I Distortions	80	8	141 385.422	3 426 252	39.28

We conducted a second test in which we generated a new supercell using the $2 \times 2 \times 2$ supercell generated by Dist_Rot 1. The I and Pb atoms were randomly displaced by a maximum of 0.2 Å, and this structure was optimized only for forces. We used computational parameters based on the results of the first test, namely 80 ppn and NCORE = 8. The estimate coast for this calculation is summarized in Table S-4. These results are important parameters that show the viability of calculations with larger structures for systems that may be of particular interest.

S-4 ADDITIONAL RESULTS FOR THE PRISTINE MAPBI$_3$ COMPOUND

After building the supercell structure for $MAPbI_3$, we investigated the potential differences arising from rotations of the MA molecules and displacements of the I atoms. To achieve this, ten different random configurations were generated. These structures were optimized using stress tensor calculations. In this section, we consolidate and analyze the results of the relaxation calculations in detail, characterizing both local and global geometrical parameters, correlations between structural and energetic properties, and electronic parameters. This analysis aims to identify the most suitable configurations for the subsequent steps of the study.

S-4.1 Structural Properties

Table S-5. Optimized values for twelve structures studied: without rotations or displaced I (Cubic), displaced I only (Cubic w-Dis), and displaced I combined with random rotations in the MA molecules (Cubic w-DisRot k). All calculations were performed while keeping the cubic box fixed during the stress-tensor calculation. The first and second columns show the structure and the associated index, respectively. The remaining columns display the values for total energy (E_{tot}), relative total energy ($\Delta E_{tot} = E_{index} - E_{\text{Cubic w/o-DisRot}}$), lattice parameter ($a_0$), volume per unit formula (V_0/f.u.), and gamma-point ($\Gamma = 0,0,0$) band gap energy (E_g).

structure	index	E_{tot} (eV)	ΔE_{tot} (eV)	a_0 (Å) (Å)	V_0/f.u. (Å^3)	E_g (eV)
Cubic w/o-DisRot	−1	−414.839	0.000	12.71	256.71	1.67
Cubic w-Dis	0	−415.699	−0.860	12.67	254.12	1.88
Cubic w-DisRot 01	1	−415.863	−1.024	12.55	247.08	1.81
Cubic w-DisRot 02	2	−415.793	−0.954	12.55	247.02	1.84
Cubic w-DisRot 03	3	−415.558	−0.719	12.60	249.93	1.74
Cubic w-DisRot 04	4	−415.585	−0.746	12.57	248.50	1.77
Cubic w-DisRot 05	5	−415.610	−0.771	12.58	248.68	1.80
Cubic w-DisRot 06	6	−415.797	−0.958	12.53	246.08	1.83
Cubic w-DisRot 07	7	−415.631	−0.792	12.56	247.91	1.80
Cubic w-DisRot 08	8	−415.583	−0.744	12.56	247.44	1.77
Cubic w-DisRot 09	9	−415.478	−0.639	12.63	251.78	1.81
Cubic w-DisRot 10	10	−415.472	−0.632	12.57	248.50	1.76

Table S-6. Optimized values for twelve structures studied: without rotations or displaced I (Cubic w/o-DisRot), displaced I only (Cubic w-Dis), and displaced I combined with random rotations in the MA molecules (Cubic w-DisRot xx). All calculations were performed while keeping the cubic box fixed during the stress-tensor calculation. The first and second columns show the structure and the associated index, respectively. The remaining columns display the values for relative total energy ($\Delta E_{tot} = E_{index} - E_{Cubic}$), lattice parameter ($a_0$), gamma-point ($\Gamma = 0, 0, 0$) band gap energy ($E_g$), point symmetry for the system (Pt.Sym) and bond angles ($\bar{\theta}_{\mathrm{Pb-I-Pb}}$).

structure	index	ΔE_{tot} (eV)	a_0 (Å) (Å)	E_g (eV)	Pt.Sym.	$\bar{\theta}_{\mathrm{Pb-I-Pb}}$ (°)
Cubic w/o-DisRot	−1	0.000	12.71	1.67	C_{1h}	169.76
Cubic w-Dis	0	−0.860	12.67	1.88	C_1	158.76
Cubic w-DisRot 01	1	−1.024	12.55	1.81	C_1	155.59
Cubic w-DisRot 02	2	−0.954	12.55	1.84	C_1	156.21
Cubic w-DisRot 03	3	−0.719	12.60	1.74	C_1	159.05
Cubic w-DisRot 04	4	−0.746	12.57	1.77	C_1	157.88
Cubic w-DisRot 05	5	−0.771	12.58	1.80	C_1	158.12
Cubic w-DisRot 06	6	−0.958	12.53	1.83	C_1	154.74
Cubic w-DisRot 07	7	−0.792	12.56	1.80	C_1	157.58
Cubic w-DisRot 08	8	−0.744	12.56	1.77	C_1	157.33
Cubic w-DisRot 09	9	−0.639	12.63	1.81	C_1	158.09
Cubic w-DisRot 10	10	−0.632	12.57	1.76	C_1	157.07

Table S-7. Geometrical and energetic properties for twelve configurations: lattice parameter (a_0) in Å, average Pb–I bond length ($d_{av}^{\mathrm{Pb-I}}$) in Å, average effective coordination number of Pb ($\mathrm{ECN}_{av}^{\mathrm{Pb}}$) in NNN, and average Pb–I–Pb bond angle ($\bar{\theta}_{\mathrm{Pb-I-Pb}}$) in °. The table also reports the relative total energy per formula unit (ΔE_{tot}) in meV/f.u., where $\Delta E_{\mathrm{tot}} = E_{xx} - E_{\mathrm{Cubic\ w/o\text{-}Dis\_Rot}}$. In addition, it presents the band gap ($E_g^{\mathrm{PBE+D3}}$) in eV. The band gap (E_g) including HSE33% and SOC effects was estimated using the scissors operator, defined from the lowest-energy structure as $\Delta_{\mathrm{SCS}} = E_g^{\mathrm{HSE33\%+SOC}} - E_g^{\mathrm{PBE+D3}}$.

Structure	a_0	$d_{av}^{\mathrm{Pb-I}}$	$\mathrm{ECN}_{av}^{\mathrm{Pb}}$	$\bar{\theta}_{\mathrm{Pb-I-Pb}}$	ΔE_{tot}	E_g^{PBE+D3}	E_g
Cubic w/o-DisRot	12.71	3.19	5.81	169.76	0	1.67	1.40
Cubic w-DisRot 01	12.55	3.22	5.96	155.59	−128	1.81	1.54
Cubic w-DisRot 02	12.55	3.22	5.92	156.61	−119	1.84	1.57
Cubic w-DisRot 03	12.60	3.21	5.96	159.05	−90	1.74	1.47
Cubic w-DisRot 04	12.57	3.21	5.98	157.88	−93	1.77	1.50
Cubic w-DisRot 05	12.58	3.21	5.96	158.12	−96	1.80	1.53
Cubic w-DisRot 06	12.53	3.22	5.96	154.74	−120	1.83	1.56
Cubic w-DisRot 07	12.56	3.21	5.96	157.88	−99	1.80	1.50
Cubic w-DisRot 08	12.56	3.21	5.96	157.33	−93	1.77	1.50
Cubic w-DisRot 09	12.63	3.22	5.95	158.09	−80	1.81	1.54
Cubic w-DisRot 10	12.57	3.21	5.95	157.07	−79	1.76	1.49

Table S-8. Relative total energies ($\Delta E_{\text{tot}} = E_{\text{index}} - E_{\text{Cubic}}$) in eV, Boltzmann factors $\left(\exp\left[-\frac{\Delta E_{\text{tot}}}{k_B T}\right]\right)$, and Boltzmann weights (P_i), where $P_i = \frac{\exp\left(-\frac{\Delta E_{\text{tot}}}{k_B T}\right)}{\sum_1^N \exp\left(-\frac{\Delta E_{\text{tot}}}{k_B T}\right)}$. All values were evaluated at $T = 300$ K.

Structure	ΔE_{tot}	$\exp\left(-\frac{\Delta E_{\text{tot}}}{k_B T}\right)$	P_i
Cubic w-DisRot 01	−128	141.35	0.24
Cubic w-DisRot 02	−119	99.80	0.17
Cubic w-DisRot 03	−90	32.50	0.06
Cubic w-DisRot 04	−93	36.50	0.06
Cubic w-DisRot 05	−96	40.99	0.07
Cubic w-DisRot 06	−120	104.73	0.18
Cubic w-DisRot 07	−99	46.04	0.08
Cubic w-DisRot 08	−93	36.50	0.06
Cubic w-DisRot 09	−80	22.08	0.04
Cubic w-DisRot 10	−79	21.24	0.04

To investigate the role of distorted octahedra in $MAPbI_3$, we analyzed the geometric parameters, as shown in Figure S-2. This analysis allowed us to examine changes in octahedral distortion and gain a better understanding of the behavior of all structures associated with these variations. Error bars were included to ensure data clarity. In the first panel, we plot the average volume of the octahedra, revealing a trend of increasing volume from the system with I-displacement (0) to the Ideal (−1) configuration. The random rotation configurations are placed between the values −1 and 0, with error bars indicating their proximity to these extremes. This trend in volume highlights the impact of rotations on volume reduction, particularly for intermediate values.

In the second panel, the average ECN_{av}^{Pb} shows a trend towards larger values in the structures with rotations, along with a slight increase from −1 to 0. Interestingly, for randomly oriented structures, the values of ECN_{av}^{Pb} remain relatively constant. This suggests that rotations play a crucial role in increasing the regularity of octahedra. This behavior may result from the disturbance of the orientation of the MA molecules, which are more polarized in the −1 and 0 configurations. The resulting electric forces elongate the octahedra. Conversely, random rotations may lead to force cancelation, resulting in octahedra adopting more regular configurations that are potentially more stable.

In the last panel, we show the average Pb-I bond distance. The values exhibit a behavior between those of the −1 and 0 configurations. Again, the error bars reveal that the values for randomly oriented structures are close to the −1 and 0 configurations. This distance can be correlated with the volume shown in the first panel, as the trend is similar, although the correlation is not perfect for structures 2 and 5, for example.

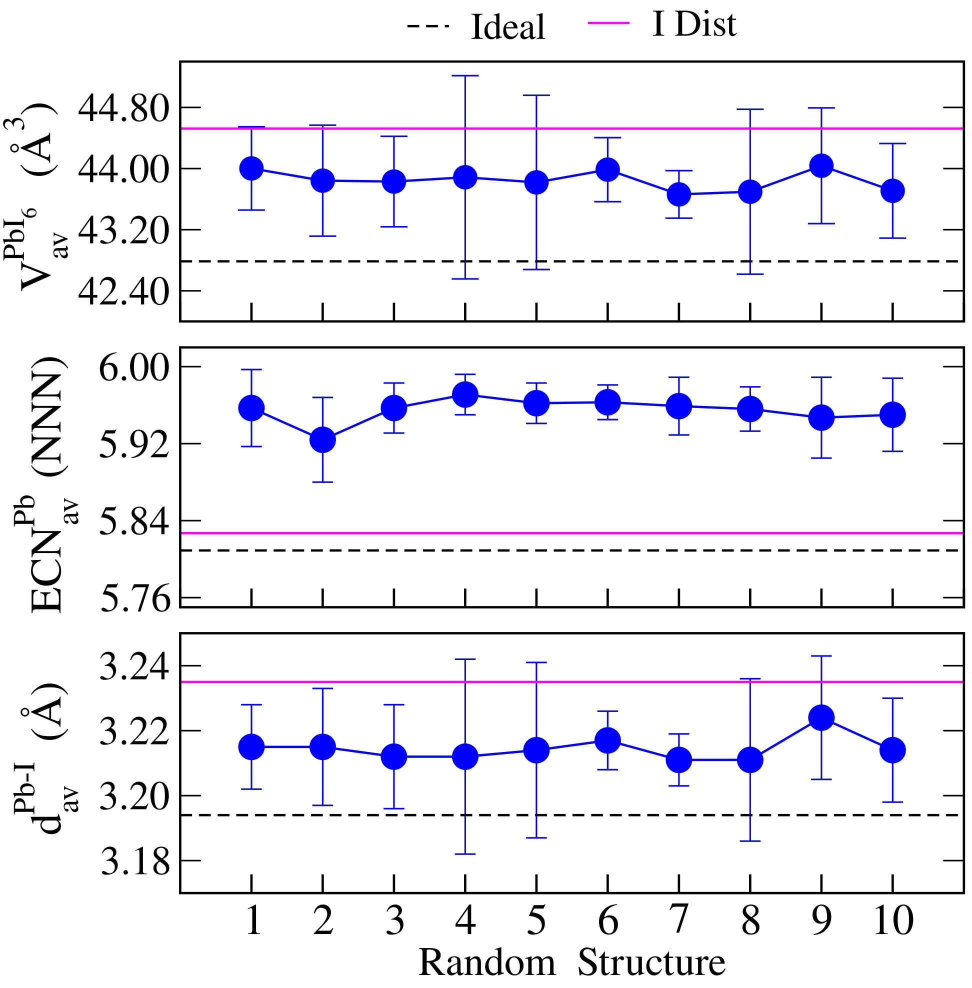

Figure S-2. Geometric parameters for each one of the twelve structures: the average volume of the PbI_6 octahedra ($V_{av}^{PbI_6}$), the average effective coordination number of the Pb atom (ECN_{av}^{Pb}), and the average Pb–I bond length (d_{av}^{Pb-I}) within the unit cell. The black dotted line and the solid purple line represent the Ideal and I Dist supercell values, respectively, while the blue vertical bars indicate the standard deviation.

Structural and Energy Parameter Correlations It is possible for a structure to exhibit different energy values and this variation may be related to one or more properties. As indicated in the literature, evidence suggests a correlation between energy and lattice parameters,[24] as well as other structural parameters. To explore the correlations

between the differential properties of all structures as a function of recovery energy, we generated Figure S-3. In the first panel on the left, only negative energy values are observed, indicating a preference for distorted structures over undistorted ones. The structure 1 exhibits the lowest energy. The roles of MA rotations and I-displacements in reducing total energy are significant. This behavior may be related to the packing of structures as a result of potential changes in octahedral configurations.

To identify trends in the plotted behavior, a linear regression analysis was performed on the values for the ten random structures. Linear regression reveals that the trends are not perfectly linear in all cases. Regarding the lattice parameter, the linear correlation between a_0 and relative energy suggests a clear trend of increasing the lattice parameter with increasing relative energy. This trend can be understood by considering that a decrease in the lattice parameter indicates a reduction in atomic distances. This phenomenon occurs when energy decreases in bonded systems, aligning with the behavior observed in our study. Furthermore, systems with energy lower than and higher than the 0 configuration were observed. This suggests that the 0 configuration allows for the construction of smaller and larger structures. However, this observation alone does not fully characterize all systems.

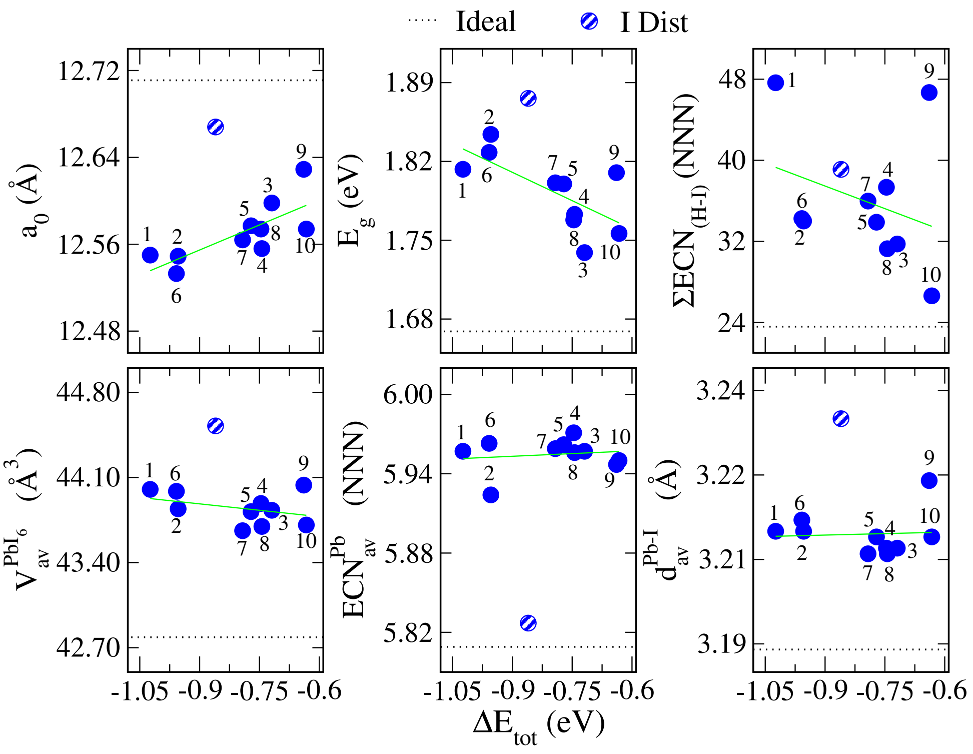

Figure S-3. Structural descriptors (y-axis) plotted against the relative total energy (x-axis). The descriptors include the equilibrium lattice parameter (a_0), the fundamental band gap at the Γ point (E_g), the sum of effective coordination numbers for H-I bonds ($\sum ECN_{\mathrm{H-I}}$), the average volume of the PbI_6 octahedra ($V_{av}^{\mathrm{PbI_6}}$), the average effective coordination number of the Pb atom (ECN_{av}^{Pb}), and the average Pb–I bond distance ($d_{av}^{\mathrm{Pb-I}}$).

In the second panel, we observe a decrease in band-gap energy with an increase in relative energy. A similar statistical distribution is observed, as in the previous case, where a group of structures exists below and above the structure 0. The band gap of all structures is greater than that of the structure −1. The literature suggests that hydrogen-bonding interactions play a significant role in systems of compounds involving MA and I. Specifically, N – H···I bonds are expected to be present.[?] To analyze these interactions, we calculated the effective coordination number for the H – I bonds across different structures and summed these values (as shown in panel 3 of Figure S-3). This method provides an estimation of the number of hydrogen bonds in the system. However, counting the hydrogen bonds reveals that this factor alone is not sufficient to explain a stable configuration. Although it may be a contributing factor, the structures 1 and 9 exhibit the same $\sum ECN$ but have opposite relative energy values.

The following panels display the average values of octahedral properties plotted against the relative energy. The descriptions of these properties reveal an approximately constant trend across all three values. This behavior suggests that the octahedra become more regular, with the variations for each structure being nearly linear. Interestingly, the reduction in relative energy cannot be attributed to internal parameters, as the octahedra appear to be more regular after random rotations. Their influence seems to be associated more with distortions between octahedra than with those within them.

Notably, octahedra tend to be more regular in systems exhibiting both distortion and rotation. A possible explanation for this observation is the alignment of the MA molecules in ordered structures. This alignment may induce an attractive force between MA and PbI, which is stronger than in cases of random rotations. Random rotations disrupt this alignment, leading to force cancelation and weaker interactions. Initial alignment promotes a stretching tendency in the octahedra. However, when this force is weakened because of disruption in alignment, the octahedra tend to become more regular. In conclusion, there is no single descriptor capable of characterizing the structure with the lowest energy. Instead, a combination of descriptors, such as distortions within octahedra and MA rotations, is required. This study provides important information on rotational systems and their characterization.

Structure and Electronic Parameter Correlations The literature suggests a correlation between the band gap and the lattice parameter. It is possible to correlate the structure and electronic parameters and evaluate this relationship for ten randomly chosen structures and the structure 0, we created a figure that illustrates the variation of the lattice parameter as a function of the $MAPbI_3$ band gap, as shown in Figure S-4. Previous literature has indicated that energy decreases with volume and lattice parameter.[24] However, in our system with random rotations, there is a trend showing a decrease in the band gap with an increase in the lattice parameter. Notably, the ideal structure has the smallest band gap and the largest lattice parameter. Structures with only I-dislocations exhibit a decrease in both the lattice parameter and the total energy, but

their band gap value is higher. The randomly generated structures generally show a decrease in volume associated with an increase in the band gap. This general behavior may be influenced by the random rotations that occur within the system.

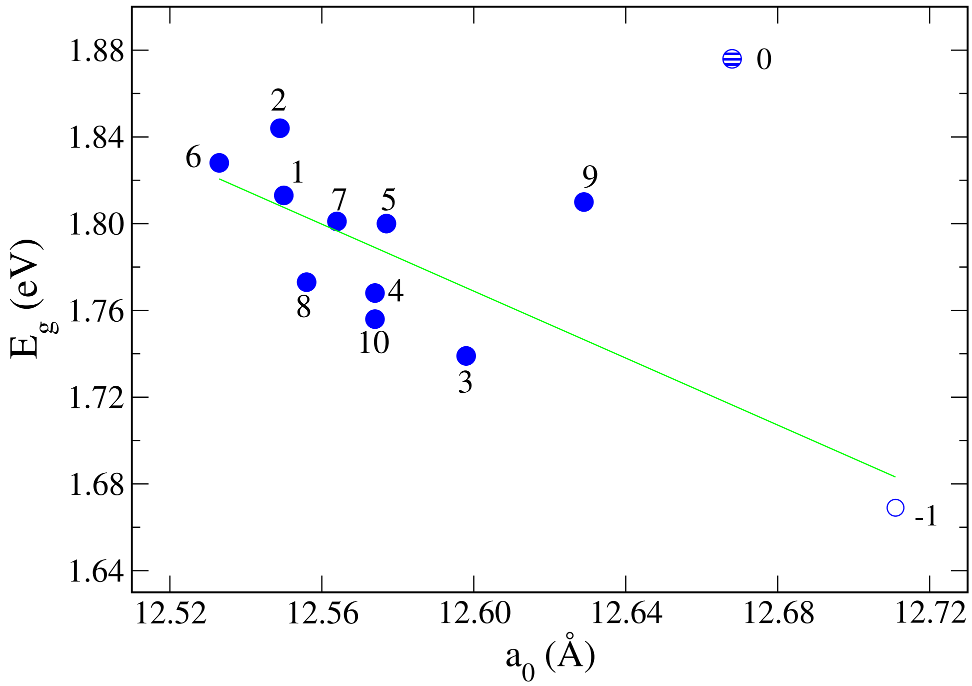

Figure S-4. Lattice parameter as a function of the band gap energy (E_g) for twelve structures: $MAPbI_3$ with I-dislocations only (index 0) and Ideal (index −1). The green line serves as a guide to the eye.

Structural Descriptors for Doping Sites: Substitutional and Interstitial The system studied in this work has several possible sites for metal doping, raising the question of which site may be preferable. Therefore, we conducted a study to investigate the feasibility of doping. The *B* site can theoretically accommodate doping at eight different positions. We compile a set of descriptors for each site, as shown in Table S-9. These parameters are crucial for describing each site and can serve as references to determine preferential sites or identify whether the system has equivalent sites. The same characterization was performed for interstitial sites, but with different parameters. A total of 16 sites were identified and their descriptors are listed in Table S-10.

Table S-9. Characterization of the parameters for each B site in the lowest energy $MAPbI_3$ structure calculated, I Dist_Rot 01; The site was defined as the sequence of Pb in the POSCAR file; Volume for the octahedron ($V^{oct.}$), average length (d_{av}), standard deviation for average length ($\sigma_{d_{av}}$), Effective Coordinate Number (ECN), and average angle for the I-Pb-I bond ($\bar{\theta}_{I-Pb-I}$), and standard deviation for average angle of the I-Pb-I bond ($\sigma_{\bar{\theta}}$).

I Dist_Rot 01-site B						
Site B	$V^{oct.}$ (Å^3)	d_{av} (Å)	$\sigma_{d_{av}}$ (Å)	ECN (NNN)	$\bar{\theta}_{I-Pb-I}$ (°)	$\sigma_{\bar{\theta}}$ (°)
1	44.893	3.233	0.044	5.967	175.083	2.576
2	44.294	3.218	0.031	5.982	175.818	1.542
3	43.865	3.223	0.087	5.868	169.269	5.592
4	43.682	3.209	0.056	5.948	172.754	2.610
5	44.543	3.230	0.042	5.970	172.335	3.524
6	43.698	3.206	0.030	5.984	174.914	5.083
7	43.861	3.211	0.057	5.945	174.524	3.040
8	43.178	3.194	0.018	5.994	174.800	2.063

Table S-10. Characterization of the parameters for each Interstitial site in the lowest energy $MAPbI_3$ structure calculated, I Dist_Rot 01; Effective Coordinate Number (ECN), the smallest distance between the bond I – Au – I (d^{short}_{I-Au-I}), the longest distance between the bond I – Au – I (d^{long}_{I-Au-I}), average length (d_{av}), standard deviation for average length ($\sigma_{d_{av}}$), and average angle for the short and long bond I-Au-I, (θ^{short}_{I-Au-I}) and (θ^{long}_{I-Au-I}), respectively.

I Dist_Rot 01-Au-Intertitial							
Site	ECN (NNN)	d^{short}_{I-Au-I} (Å)	d^{long}_{I-Au-I} (Å)	d_{av} (Å)	$\sigma_{d_{av}}$ (Å)	θ^{short}_{I-Au-I} (°)	θ^{long}_{I-Au-I} (°)
1	2.006	5.415	7.656	3.272	0.668	179.999	172.655
2	2.001	4.898	7.447	3.113	0.782	179.998	160.553
3	2.007	5.257	7.377	3.171	0.627	171.634	152.058
4	2.012	5.188	7.160	3.143	0.629	180.000	151.709
5	2.176	5.524	7.067	3.155	0.454	179.999	169.589
6	2.331	5.610	6.823	3.156	0.406	179.998	153.181
7	2.400	5.440	7.060	3.232	0.452	179.999	165.345
8	2.094	5.727	7.142	3.156	0.508	179.998	158.602
9	2.008	5.243	7.370	3.164	0.628	179.999	167.830
10	2.013	5.297	7.255	3.172	0.633	179.999	157.944
11	2.007	5.216	7.308	3.148	0.625	179.998	164.305
12	2.005	5.303	7.437	3.210	0.645	179.997	161.254
13	2.050	5.270	7.167	3.110	0.557	179.998	176.604
14	2.005	5.410	7.684	3.282	0.668	179.999	168.979
15	2.000	4.873	7.504	3.103	0.770	179.999	169.022
16	2.000	4.949	7.750	3.176	0.814	179.998	175.192

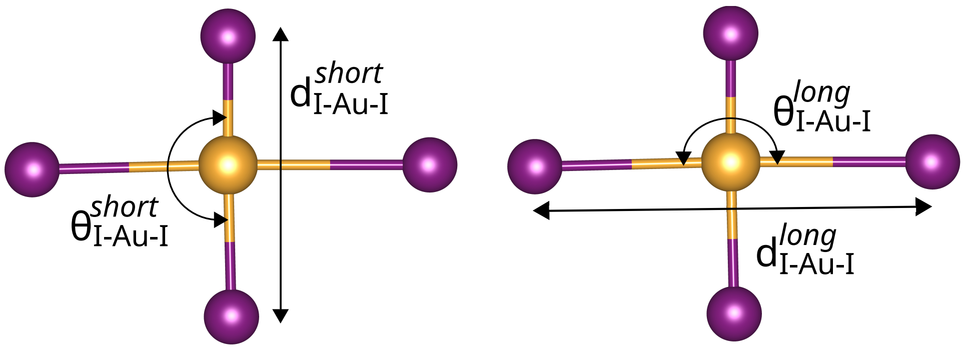

Figure S-5. Schematic representation of the angles and bond lengths, both short and long, considered in this study. Yellow and purple spheres represent Au and I atoms, respectively.

S-4.2 Total and Local Density of States

It is known to contribute to the formation of the valence and conduction bands and that metals can introduce states into the band gap of materials,[25] thereby altering the electronic and optical properties of perovskite materials and influencing their performance as solar cells. To investigate the electronic structure and provide a complete characterization of the structure without metals or other point defects, we selected two structures: one with the highest energy (Ideal) and the other with the lowest energy (1). We then generated graphs of the total density of states and band structures for both structures, as shown in Figure S-6. In this figure, certain features of the system are evident. For example, there is a greater contribution from MA atoms to deep states with localized characteristics. This point is important because it highlights the different contributions of MA to the structural properties due to the deep states.

However, the electronic properties are primarily associated with the octahedra, with contributions from I (the majority) and Pb (the minority) states observed at the top of the valence band (VBM), near the Fermi level. This pattern also reveals a strong influence of I on the optical properties of the material as a result of its major contribution of states near the VBM. Below the Fermi level and up to the band gap, the contribution of the Pb states is visible, but without any significant difference compared to the other atomic species. This analysis is consistent for both structures in Figure S-6.

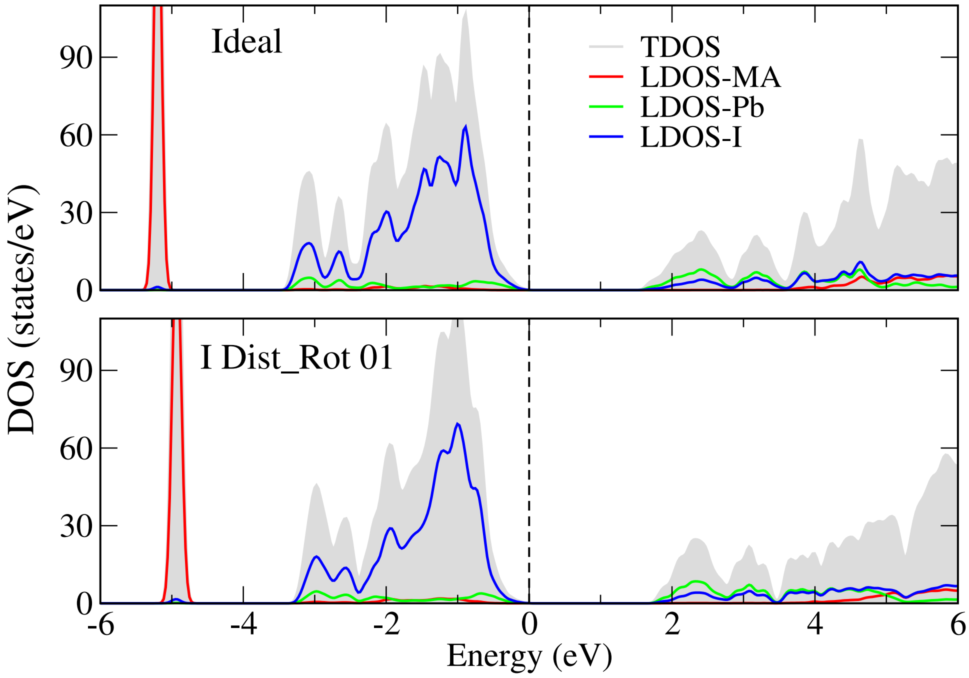

Figure S-6. Total density of states for the highest and lowest energy structures using the PBE+D3 functional. The gray area represents the total DOS, while the red, green, and blue lines represent the DOS for MA, Pb, and I, respectively. The dashed vertical line indicates the Fermi level.

For a more detailed characterization, we plotted the Local Density of States (LDOS), which illustrates the orbital contributions of the s, p, and d orbitals for each atomic species, namely MA, Pb, and I. This plot is presented in Figure S-7. Predominantly, there is a significant contribution from the orbitals both below and above the Fermi level in most cases. Specifically, the total energy density for the structures with the lowest and highest energies reveals a pronounced contribution from the s orbital of Pb below the Fermi level. Regarding the deep states, it should be noted that the orbitals s and p of MA are particularly significant, exhibiting localized states. This observation aligns with the DOS results for these states. Furthermore, the overlapping states of the s and p orbitals for MA are observed near 5 eV, indicating hybridization of these states. Furthermore, in the vicinity of the Fermi level, a notable contribution is evident from the p orbitals of the I atoms in the valence band. This analysis is consistent for both structures.

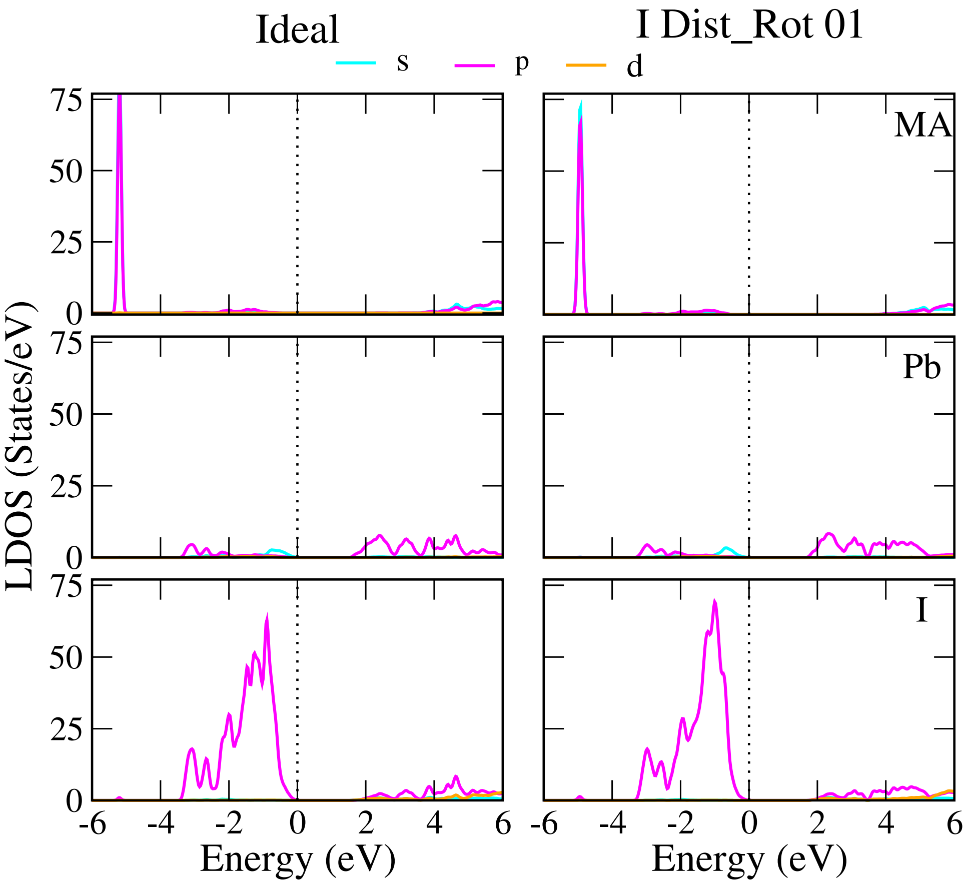

Figure S-7. Local density of states (LDOS) for MA, Pb, and I, including the sum of the contributions from each orbital. The dashed vertical line represents the Fermi level, and all data were obtained using the PBE+D3 functional.

S-4.3 Electronic Band Structures

PBE+D3 We calculated the band structure for $MAPbI_3$ using the PBE functional for two structures, −1 and 1, which represent the lowest and highest energy configurations, respectively. Our results are shown in Figure S-8. The expected semiconductor behavior is observed in the figure, with the band gaps at the gamma point (Γ). The band gap value shows a difference of approximately 0.14 eV. This difference is a relevant consequence of the lowest-energy structures obtained with I displacements and MA rotations. Nevertheless, it is known that the PBE functional underestimates the band gap value, and for this reason additional calculations with higher-level functionals, such as Spin-Orbit Coupling (SOC) and HSE, are needed. In the next section, we discuss the results for the SOC band structure.

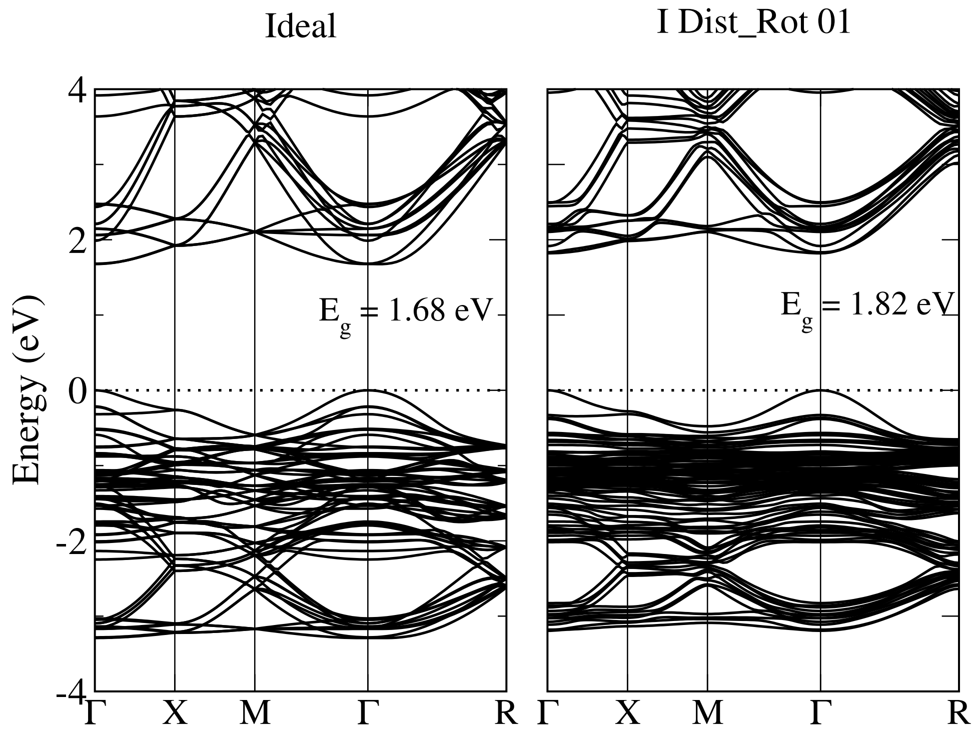

Figure S-8. Electronic band structures calculated at the PBE+D3 level for two structures: Ideal (index −1) and I Dist_Rot 01 (index 1). States below the black dashed line are occupied, while those above are unoccupied.

PBE+D3+SOC Heavy atoms require corrections that account for the interactions between the angular momentum and the spin moment. These interactions can significantly influence the electronic properties of materials, such as changes in the band gap. Specifically, for perovskites, these corrections are known to lead to a reduction in the band gap. This reduction occurs because of the breaking of the degeneracy in the states when considering all possible combinations of angular momentum and spin moment. Many configurations, which were previously counted multiple times due to spin polarization (up and down), are accounted for only once in this approach. The results show a decrease in the band gap with the emergence of new states above the Fermi level, as depicted in Figure S-9. The effect of Spin-Orbit Coupling (SOC) is to clarify the bands, resulting in a decrease in the band gap by 0.78 eV. This value is less than the experimental and theoretical values of 1.52 eV and 1.60 eV, respectively. For this reason, in the next section, we conducted tests using the Hybrid Functional (HSE06) in the next section.

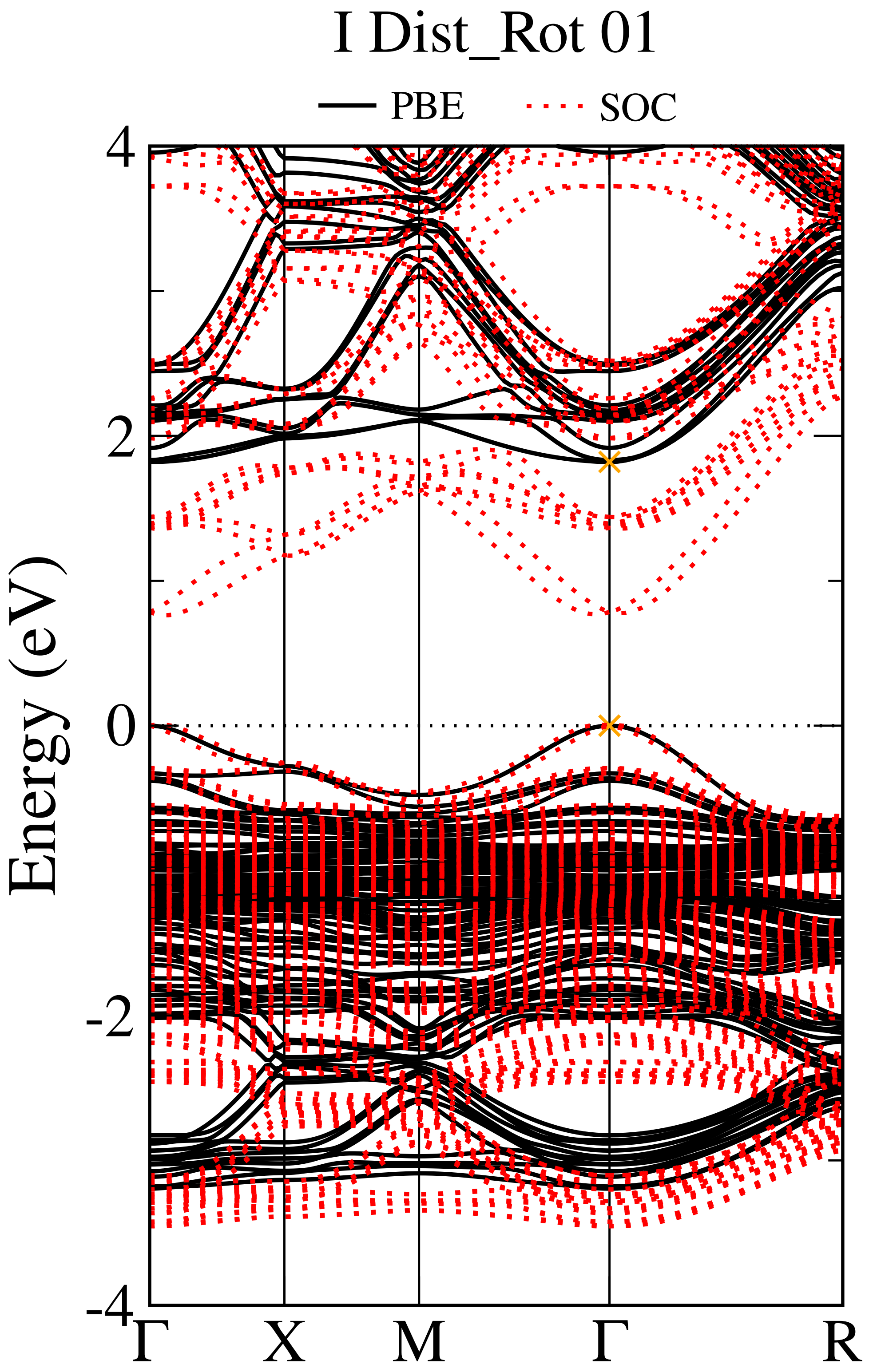

Figure S-9. Electronic band structure of $MAPbI_3$ calculated with SOC (red) and without SOC (black), using the GGA–PBE exchange–correlation functional. The red dashed line indicates the Fermi level.

Band Gap Corrections The Heyd–Scuseria–Ernzerhof (HSE) functional can calculate the band gap by adjusting a parameter that incorporates a percentage of the exact

Hartree–Fock function into the exchange functional approximation. Computationally, this percentage is adjusted using the α parameter. Typically, most calculations use $\alpha = 0.25$, and in this context it is commonly referred to as HSE06. However, utilizing the hybrid functional increases computational costs. Therefore, we performed calculations only at the Γ point and performed tests using PBE+D3 combined with HSE, as well as PBE+D3+SOC with HSE, varying the values of the parameter α to 0.25 and 0.33. The results are presented in Table S-11. The use of the PBE+D3 functional underestimates the band gap, while SOC decreases these values, PBE+D3 with HSE06 increases the band gap. Both effects on the band gap could yield more accurate results closer to the experimental values. However, it is necessary to correct for the value of the parameter α. We adjusted this parameter using the experimental value and found that $\alpha = 0.33$ provided the most appropriate correction for the band gap value (1.54 eV to 1.52 eV of the experimental value)[9]. For this reason, we will perform HSE33%+SOC calculations with the α parameter set to 0.33 at the Γ point in all future calculations.

Table S-11. Computational cost parameters for simulations at different theoretical levels: processors per node (ppn), α hybrid E_{xc} mixing parameter, number of cores (NCORE), calculation time, maximum RAM usage, and band gaps at the Γ point (E_g^Γ).

Theoretical level	α	ppn	NCORE	Time(h)	Memory (kb)	E_g^Γ (eV)
PBE+D3	-	20	5	0.03	369 160	1.82
PBE+D3+SOC	-	40	5	1.00	1 029 056	0.71
HSE06+PBE+D3	0.25	20	5	2.46	596 168	2.42
HSE06+SOC	0.25	40	5	24.75	1 091 180	1.32
HSE33%+SOC	0.33	20	5	17.83	1 364 812	1.54

S-5 ADDITIONAL RESULTS: TESTS FOR AU POINT DEFECTS IN $MAPBI_3$

In this section, we used the result of the previous step, where it was determined that I Dist_Rot 01 structure has the lowest energy (Section S-4). Therefore, our system with dopants was generated on the basis of this structure. Furthermore, we conducted tests considering that our review shows that only sites B and I_{TM} are significant when evaluating metals Au, Ag, Ni, and Cu for doping. Specifically, the well-known metal

Au was used for initial tests at both doping sites and with different charge states, as described in Tables S-13 and S-15.

S-5.1 Tests for Dopants at Different Substitutional Sites

Using data from Table S-9, we conducted tests to assess the possible preference for doping at the B site and its impact on properties when different B sites are considered. We performed tests with Au for the smallest formation energy charge states, −1 and 0, doping at sites 8, 3, and 1, which represent the smallest, medium and largest values for the parameters $V^{oct.}$, ECN, and d_{av}. The results of these tests are shown in Table S-12. The data indicate that the largest energy difference between the systems is approximately 0.16 eV. This value represents the error associated with our calculations using the PBE+D3 functional and will be considered as the imprecision in calculating the formation energy. However, this does not pose a significant issue, as the functional is capable of determining the energy trend, which is crucial to selecting the system with the lowest formation energy. Subsequently, the screened system will be calculated with higher accuracy using the HSE and SOC functionals.

Table S-12. Relative energies for Au-doped $MAPbI_3$ at non-equivalent B substitutional sites. Data for charge states $q = 0$ and $q = -1$ were analyzed. The relative energy was calculated as $\Delta E_{\text{tot}} = \Delta E^{i}_{\text{tot}} - \Delta E^{\text{ref}}_{\text{tot}}$, where ref denotes the system with the lowest total energy.

Site	Charge State (e)	ΔE_{tot} (eV)
1	0	0.160
	−1	0.145
3	0	0.096
	−1	0.000
8	0	0.000
	−1	0.111

Table S-13. Structural descriptors of optimized geometries obtained at the PBE+D3 level for $MAPbI_3$ doped with Au at the B site. Reported values include the octahedral volume ($V^{\text{oct.}}$), average bond distance (d_{av}), and Effective Coordination Number (ECN) for the charge states with the lowest formation energy.

Doping with Au at site B						
Site B	$q = 0$			$q = -1$		
	$V^{oct.}$ (Å^3)	d_{av} (Å)	ECN (NNN)	$V^{oct.}$ (Å^3)	d_{av} (Å)	ECN (NNN)
1	45.671	3.254	5.956	45.580	3.252	5.965
2	44.606	3.227	5.976	44.227	3.219	5.944
3	44.204	3.229	5.903	43.935	3.221	5.908
4	45.402	3.248	5.914	44.117	3.216	5.957
5	44.845	3.238	5.954	44.941	3.240	5.965
6	44.436	3.225	5.984	43.719	3.223	5.566
7	44.655	3.230	5.971	44.257	3.236	5.564
8	36.429	3.013	5.961	40.409	3.582	2.010

S-5.2 Tests for Dopants at Different Interstitial Sites

Testing different configurations of the system doped with Au interstitials. Based on the descriptors provided in Table S-10, we identified three representative sites for the system: those with the smallest, middle and largest values of the descriptor $d^{short}_{\text{I-Au-I}}$ (sites 1, 8, and 15). The results are shown in Table S-14, indicate that the energy differences between these sites are minimal, approximately 0.039 eV. This suggests that these sites can be considered equivalent for the purposes of this study, allowing for a more focused investigation of a single interstitial site without the need to explore all potential sites.

Table S-14. Relative energies for Au-doped $MAPbI_3$ at non-equivalent interstitial sites. Data for charge states $q = 0$ and $q = +1$ were analyzed. The relative energy was calculated as $\Delta E_{\text{tot}} = \Delta E^{i}_{\text{tot}} - \Delta E^{\text{ref}}_{\text{tot}}$, where ref denotes the system with the lowest total energy.

Site	Charge State (e)	ΔE_{tot} (eV)
1	0	0.030
	1	0.039
8	0	0.037
	1	0.032
15	0	0.000
	1	0.000

Table S-15. Structural descriptors of optimized geometries obtained at the PBE+D3 level for $MAPbI_3$ doped with Au at interstitial sites. Reported values include the octahedral volume ($V^{oct.}$), average bond distance (d_{av}), and Effective Coordination Number (ECN) for the charge states with the lowest formation energy.

Doping Interstitial with Au						
Site	$q = 0$			$q = +1$		
	$V^{oct.}$ (Å^3)	d_{av} (Å)	ECN (NNN)	$V^{oct.}$ (Å^3)	d_{av} (Å)	ECN (NNN)
1	45.200	3.243	5.892	45.119	3.247	5.836
2	43.522	3.206	5.964	43.513	3.208	5.955
3	43.996	3.218	5.923	43.977	3.225	5.822
4	42.753	3.184	5.975	42.785	3.190	5.948
5	45.051	3.243	5.909	45.066	3.246	5.878
6	43.751	3.203	5.968	43.697	3.202	5.957
7	44.171	3.219	5.951	44.108	3.219	5.940
8	42.834	3.182	5.992	42.811	3.182	5.989

S-5.3 Au Doping at Substitutional B and Interstitial Sites

In this section, we provide complementary data on doping with Au at the B site. The doping process involved the introduction of the Au metal at the B site. The initial supercell was a configuration $2 \times 2 \times 2$ (96 atoms), which was modified by replacing a Pb atom with an Au atom, resulting in a structure with 96 atoms. Initial calculations were performed for doping at the $Pb - 8$ site, and the formation energy was calculated for different charge states. The same structure was then used for interstitial doping with Au, resulting in a supercell with 97 atoms.

To evaluate the possibility of generating the system with the defect, we calculated the formation energy (E_F), as described by the following equation:

$$E_F(TM, q) = E_{tot}(TM, q) - E_p - \sum_i \mu_i n_i + q(\mu_E + E_{\text{VBM}} + \Delta E) + E_{corr}, \quad \text{(S-1)}$$

$E_{tot}(TM, q)$ is the total energy of the $MAPbI_3$ supercell doped with transition metal (TM) with an excess charge q. E_p is the total energy of the pristine system; μ_{TM} is the chemical potential of the transition metal TM, calculated as the total energy per atom of a monoelemental bulk system. μ_E is the electronic chemical potential that varies from 0 to the band gap value of 1.84 eV. E_{VBM} is the eigenvalue or maximum valence band

obtained from the pristine supercell, and ΔE is the term that aligns the VBM values of the pristine and doped systems. E_{corr} is the correction term that accounts for the interactions between the charged system and its images in the periodic calculations.

Since the eigenvalues for the pristine and doped systems differ, it is necessary to align their energy levels for consistency. To address this, we applied the correction ΔV in Equation S-1. This correction accounts for the difference in eigenvalues between the pristine and doped systems for charge states $q = 0$ and $q = +1$. Specifically, we used the eigenvalues of the lead species (Pb) from our calculations. We calculated the difference between the eigenvalues of the pristine and doped systems for each Pb atom and averaged these differences to determine ΔV. This correction term ensures that the energy levels are rescaled to the same reference scale, allowing the correct calculation of the formation energy (E_F).

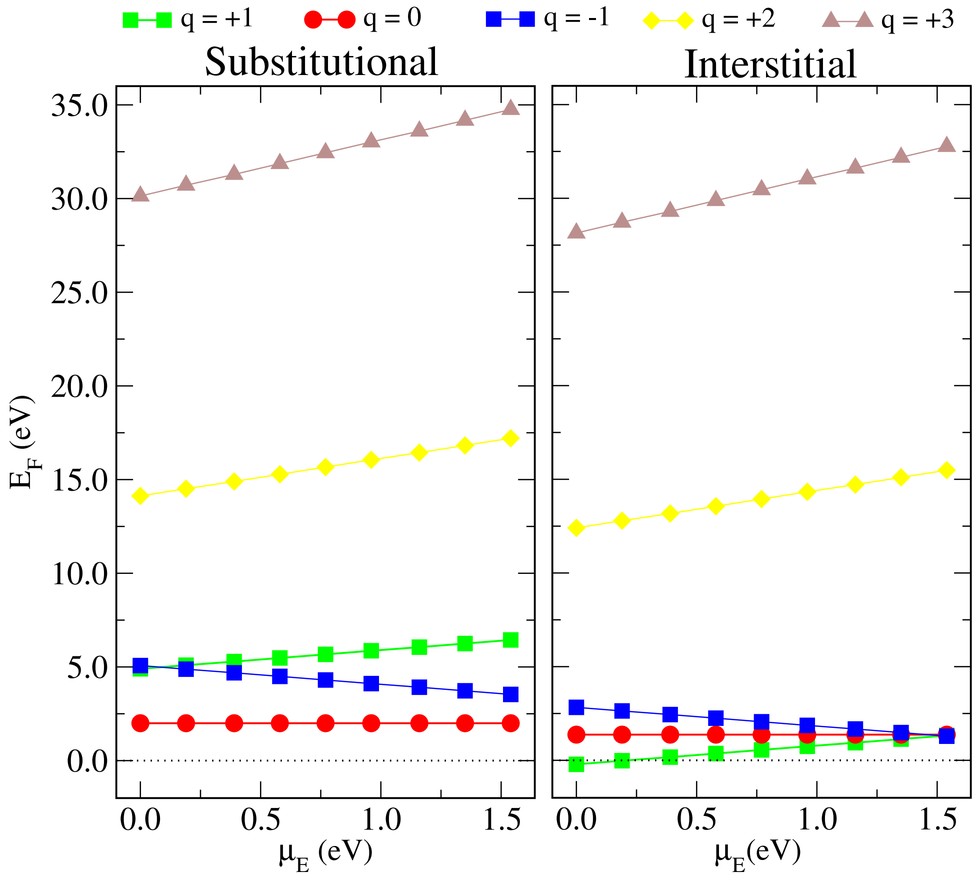

Figure S-10. Formation energies (E_F) of the Au-doped $MAPbI_3$ system at the B site (substitutional) and the interstitial site, considering charge states $q = 0$, +1, −1, +2, and +3. Calculations were performed with the HSE33% exchange–correlation functional including spin–orbit coupling (SOC), sampling the reciprocal space at the Γ point.

To validate our results, we compared them with data from the literature on specific Au point defects, focusing on doping at the B site and the interstitial site. Our calculations of the formation energy (E_F) revealed that the charge states $q = +2$ and $q = +3$ have the highest formation energies, which is consistent with the findings in the literature where the focus is on the charge states $q = 0$ and $q = +1$.[8] The substitutional state $q = -1$ exhibits a lower energy than $q = +1$, but our calculated E_F is higher than that of the interstitial $q = 0$ and $q = +1$. Consequently, this state is not relevant in our analysis or in the literature. When considering the $q = 0$ and $q = +1$ states, our results are comparable for both types of defects. However, interstitial doping with Au in the charge states $q = 0$ and $q = +1$ shows the lowest formation energy and offers a better range of variation in the electronic potential, which aligns well with the literature. The trend observed for interstitial doping matches that in the literature, including the region where exothermic behavior is observed for Au with $q = +1$. Despite this, a small difference of approximately 0.3 eV in E_F is observed, which we attribute to the smaller size of our structure. This difference may amplify the effect of Au atoms in our simulation compared to the model used by Kerner *et al.*, which utilizes a larger system.

S-6 ADDITIONAL RESULTS FOR POINTS DEFECTS IN $MAPBI_3$

In this step, we used different types of point defects based on the information collected in the literature review, as presented in Section S-2. Our review shows that only the B and I_{TM} sites are important when considering metals such as Au, Ag, Ni, and Cu for doping. Furthermore, certain charge states are relevant for the study of metal doping, as indicated in Table S-2. For the systems studied in the subsequent step, it was determined that the I Dist_Rot 01 structure has the lowest energy (Section S-4); therefore, our doped system was generated based on this structure. Additional details on this analysis are presented in this section.

S-6.1 Additional Formation Energy Data

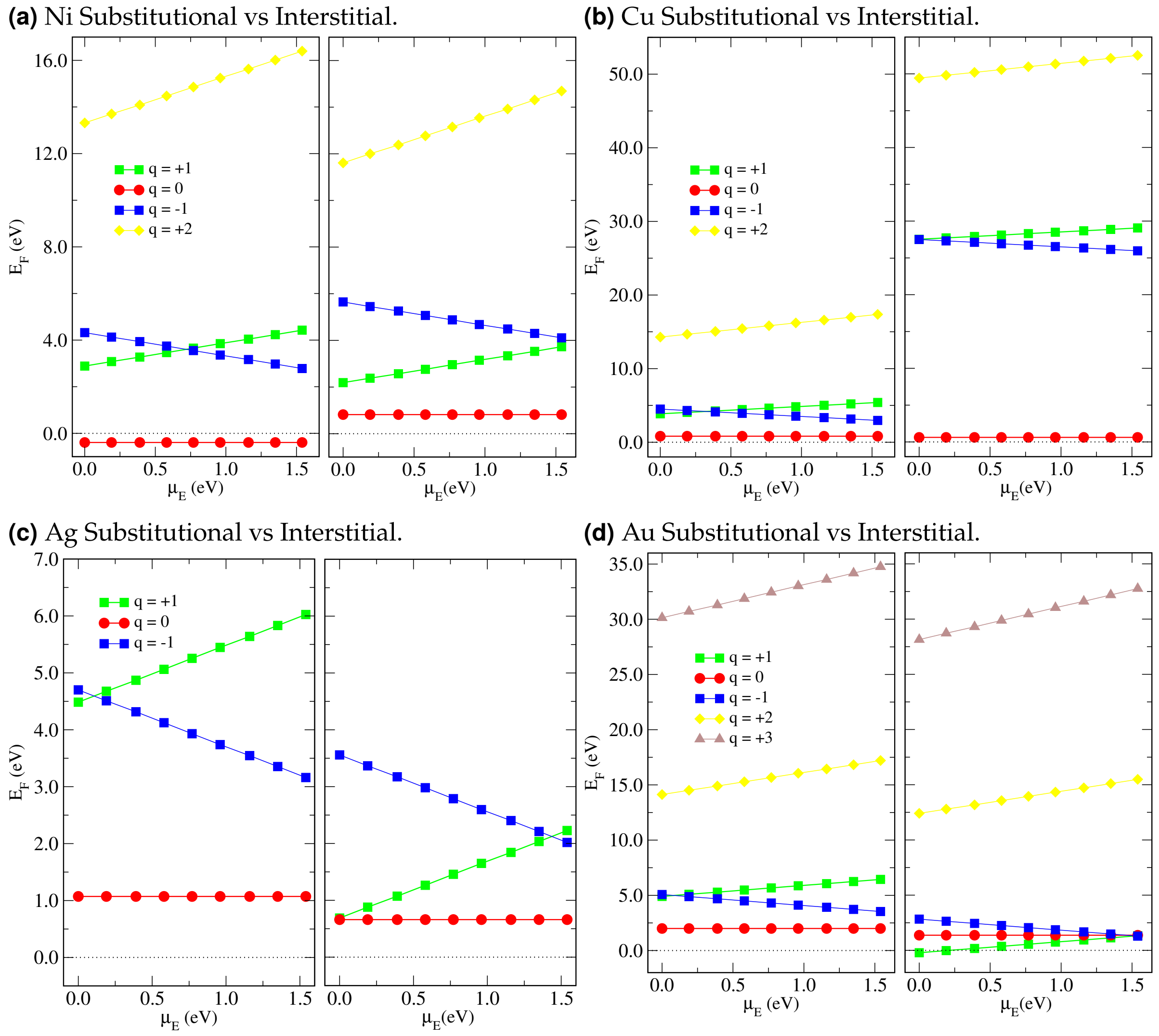

Figure S-11. Formation energies (E_F) of point defects incorporating Ni, Cu, Ag, and Au adatoms (panels a–d, respectively), considering charge states q reported in the literature. Calculations were performed with the HSE33% exchange–correlation functional including spin–orbit coupling (SOC), sampling the reciprocal space at the Γ point. Values correspond to doping at B and interstitial sites.

S-6.2 Additional Electronic Data

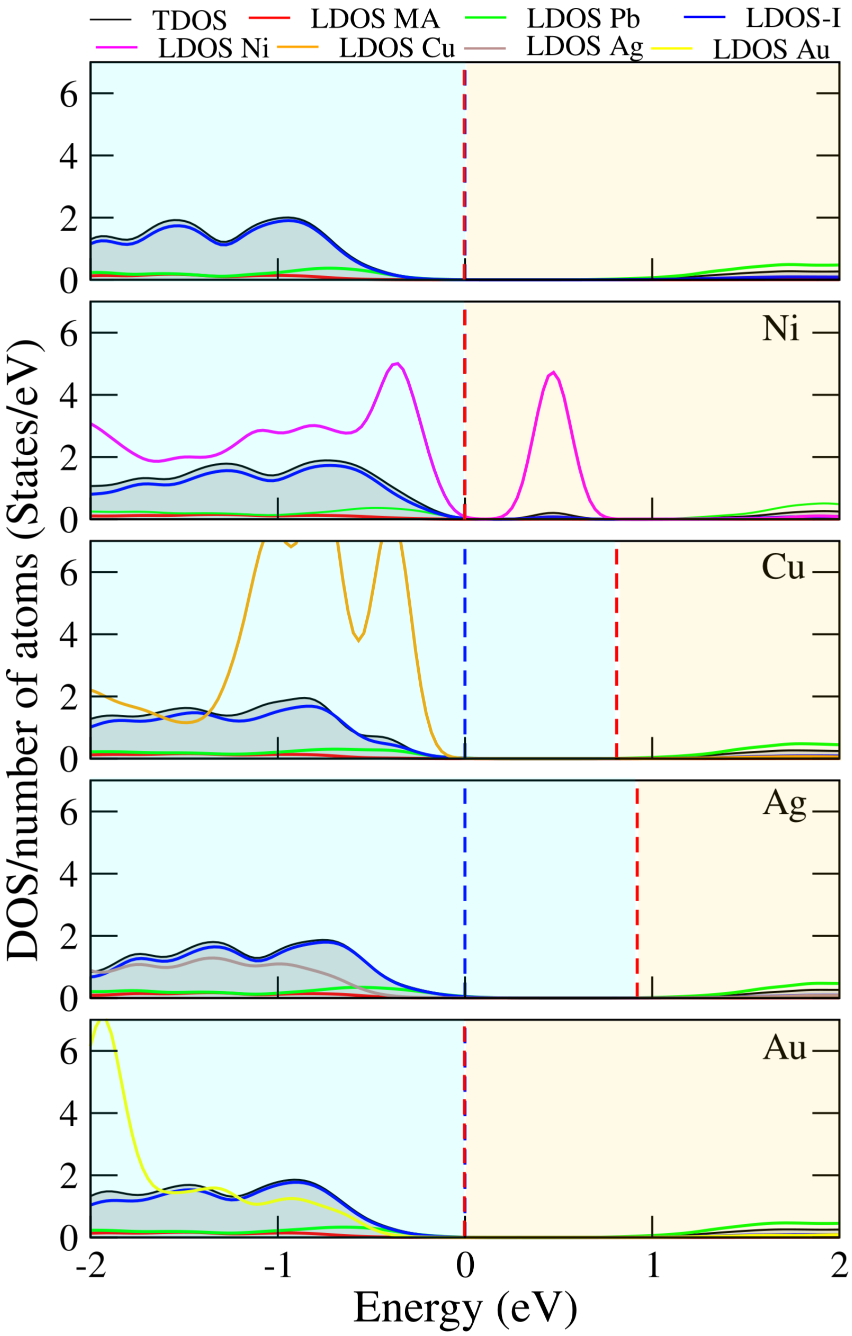

Figure S-12. Density of states (DOS) normalized by the number of atoms (DOS/number of atoms) for undoped and metal-doped $MAPbI_3$ systems, shown for configurations with the lowest formation energies E_F. The plots span the energy range from −2 to 2 eV. The dashed red line indicates the Fermi level, while the dashed blue line marks the valence band maximum (VBM), set as the energy zero. Blue and yellow regions correspond to occupied and empty states, respectively. All calculations were performed at the PBE+SOC level.

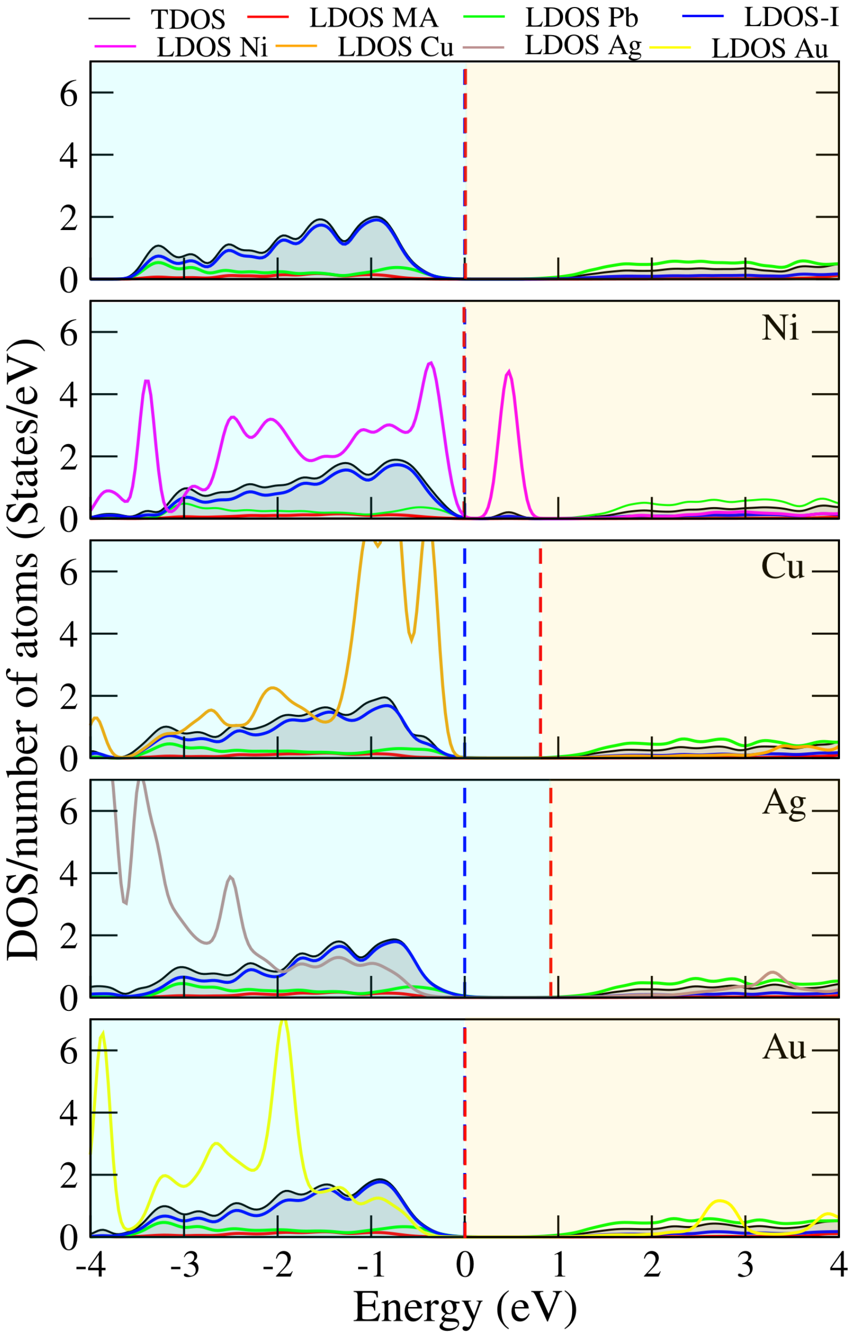

Figure S-13. Density of states (DOS) normalized by the number of atoms (DOS/number of atoms) for undoped and metal-doped $MAPbI_3$ systems, shown for configurations with the lowest formation energies E_F. The plots span the energy range from −4 to 4 eV. The dashed red line indicates the Fermi level, while the dashed blue line marks the valence band maximum (VBM), set as the energy zero. Blue and yellow regions correspond to occupied and empty states, respectively. All calculations were performed at the PBE+SOC level.

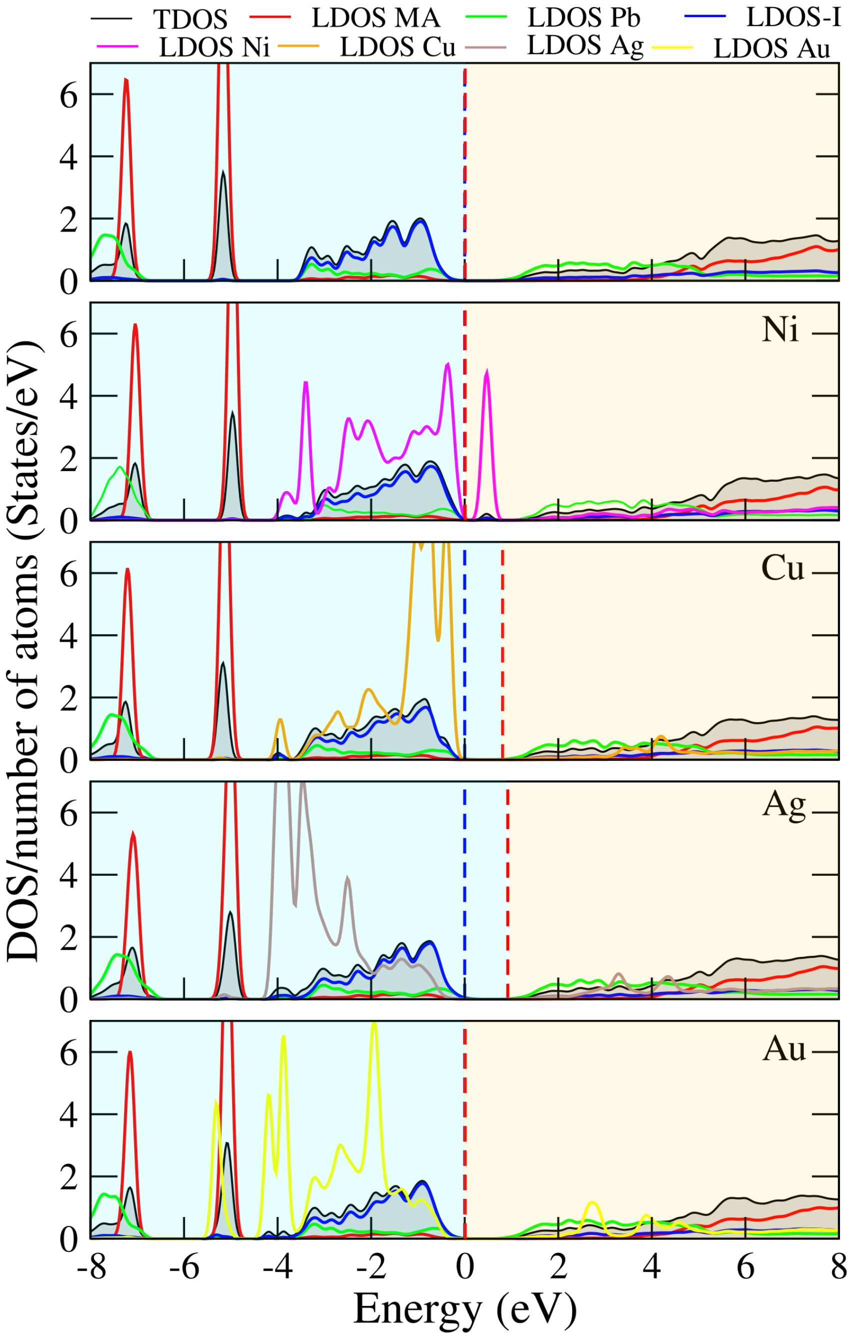

Figure S-14. Density of states (DOS) normalized by the number of atoms (DOS/number of atoms) for undoped and metal-doped $MAPbI_3$ systems, shown for configurations with the lowest formation energies E_F. The plots span the energy range from −8 to 8 eV. The dashed red line indicates the Fermi level, while the dashed blue line marks the valence band maximum (VBM), set as the energy zero. Blue and yellow regions correspond to occupied and empty states, respectively. All calculations were performed at the PBE+SOC level.

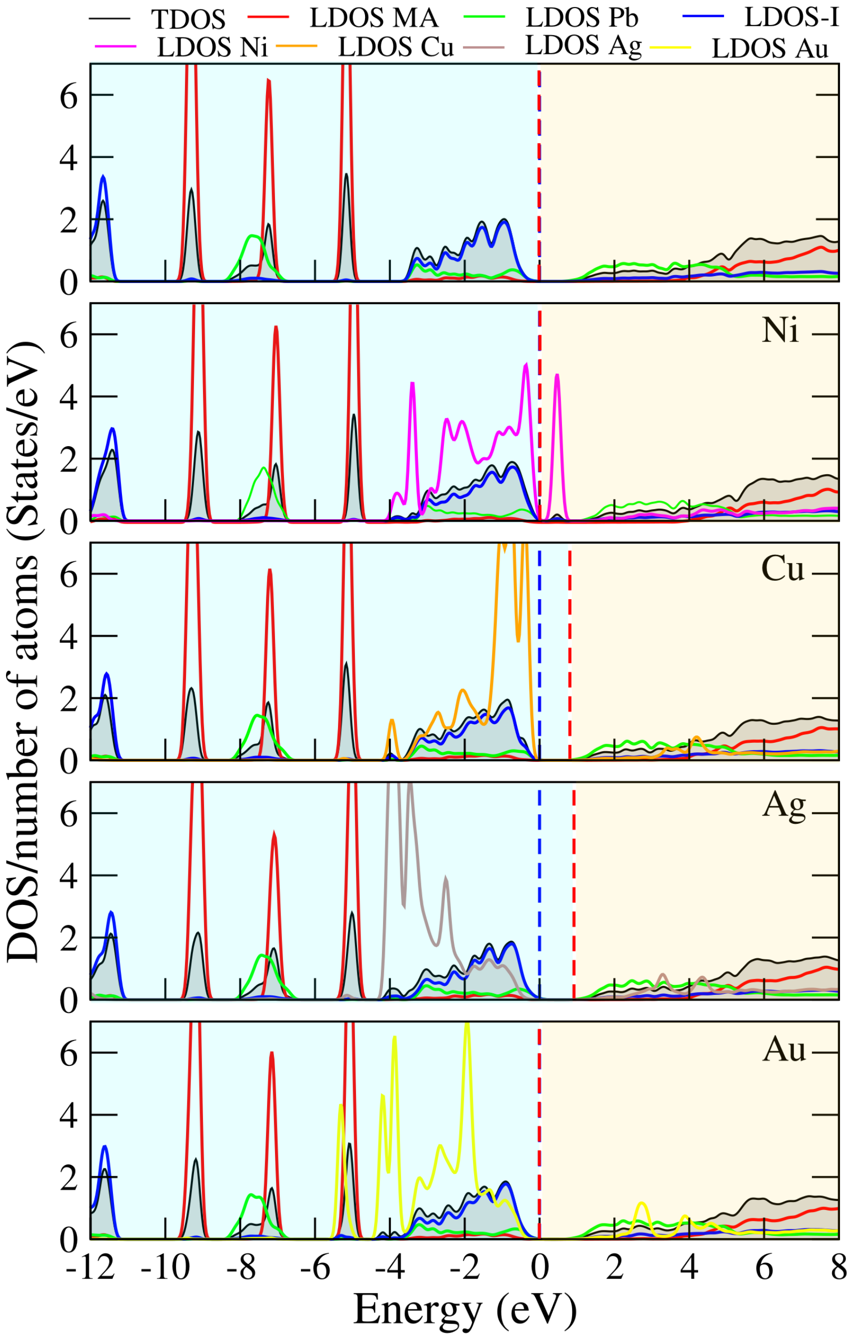

Figure S-15. Density of states (DOS) normalized by the number of atoms (DOS/number of atoms) for undoped and metal-doped $MAPbI_3$ systems, shown for configurations with the lowest formation energies E_F. The dashed red line indicates the Fermi level, while the dashed blue line marks the valence band maximum (VBM), set as the energy zero. Blue and yellow regions correspond to occupied and empty states, respectively. All calculations were performed at the PBE+SOC level.

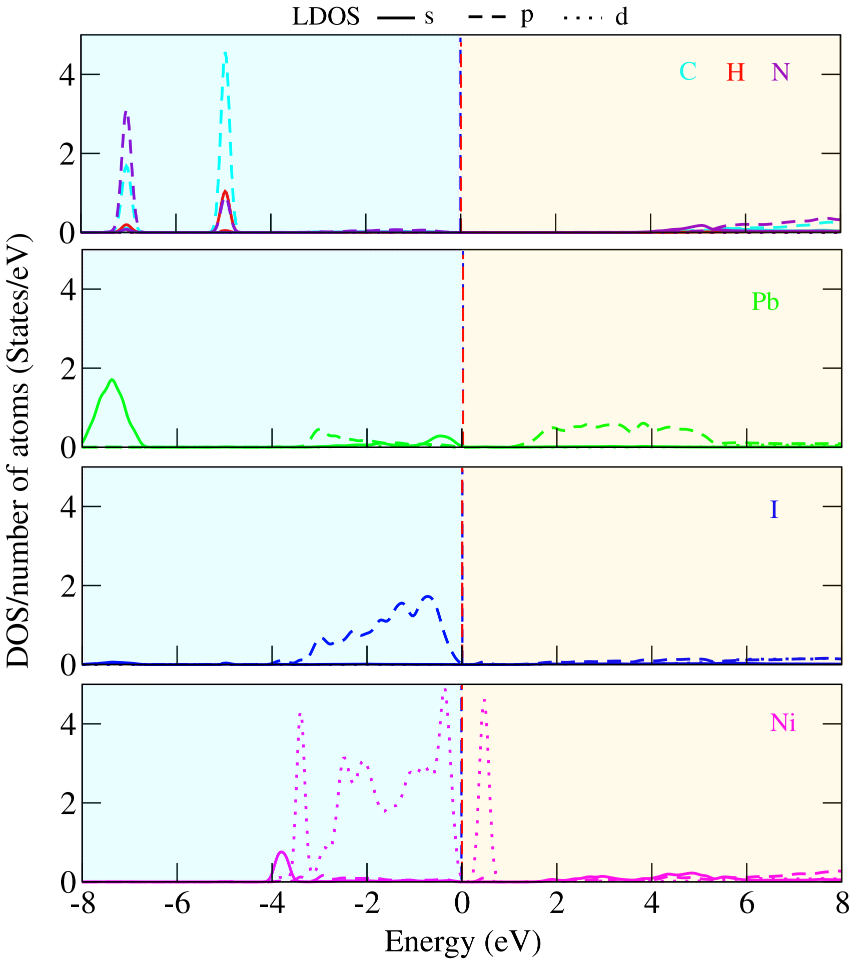

Figure S-16. Local density of states (LDOS) at the PBE+SOC level for the Ni-doped $MAPbI_3$ in the configuration with the lowest formation energy E_F. The LDOS is resolved into s, p, and d orbitals and normalized by the number of atoms (LDOS/number of atoms). The dashed red line indicates the Fermi level, while the dashed blue line marks the valence band maximum (VBM), set as the energy zero. Blue and yellow regions correspond to occupied and empty states, respectively.

(a) Ni Substitutional.

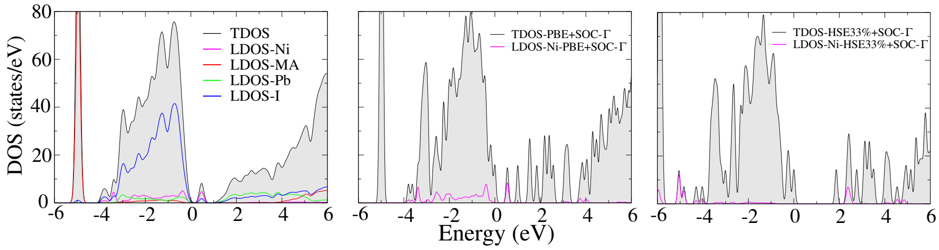

(b) Cu Interstitial.

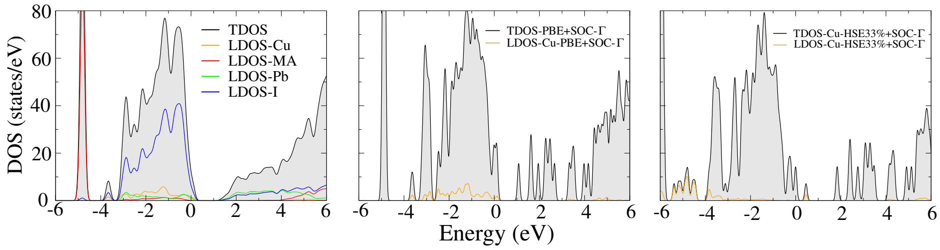

(c) Ag Interstitial.

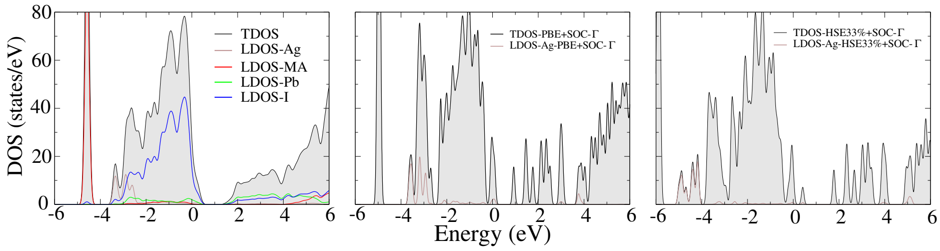

(d) Au Interstitial.

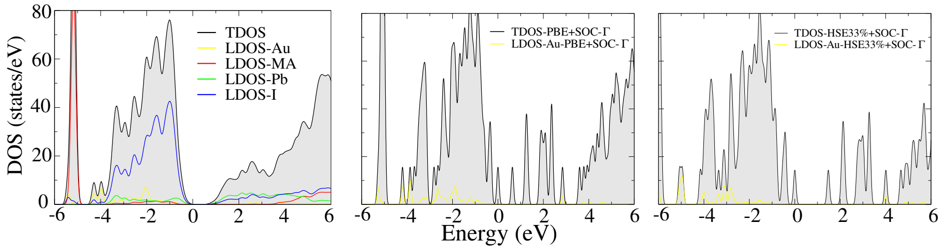

Figure S-17. Density of states (DOS) for $MAPbI_3$ doped with Ni, Cu, Ag, and Au metal adatoms (panels a–d, respectively), shown for configurations with the lowest formation energies E_F. The left panels display DOS calculated using a 4×4×4 **k**-point grid including spin–orbit coupling (SOC) effects. The middle panels present simulations performed at the PBE+SOC level using only the Γ point for reciprocal-space sampling. The right panels show results obtained with HSE33%+SOC using only the Γ point.

(a) Ni Substitutional.

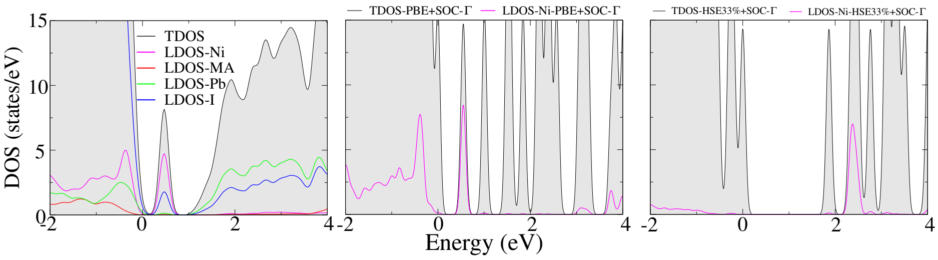

(b) Cu Interstitial.

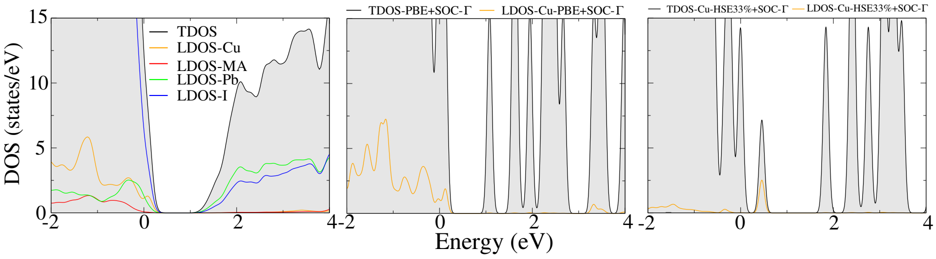

(c) Ag Interstitial.

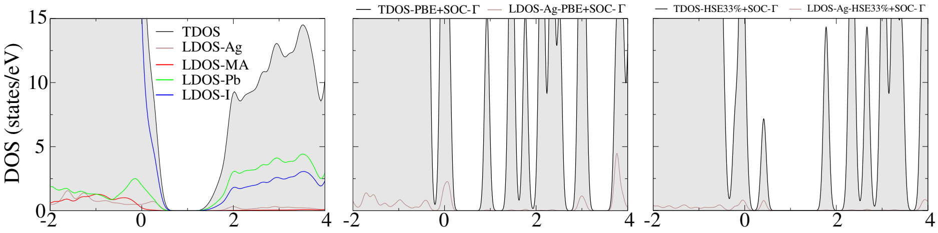

(d) Ni Interstitial.

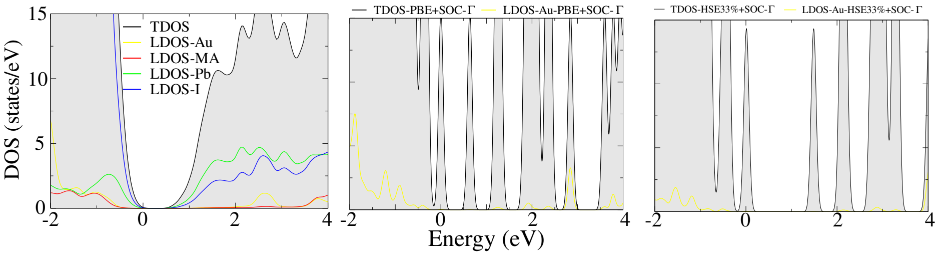

Figure S-18. Density of states (DOS) below 15 states/eV for $MAPbI_3$ doped with Ni, Cu, Ag, and Au metal adatoms (panels a–d, respectively), shown for configurations with the lowest formation energies E_F. The left panels display DOS calculated using a 4×4×4 **k**-point grid including spin–orbit coupling (SOC) effects. The middle panels present simulations performed at the PBE+SOC level using only the Γ point for reciprocal-space sampling. The right panels show results obtained with HSE33%+SOC using only the Γ point.

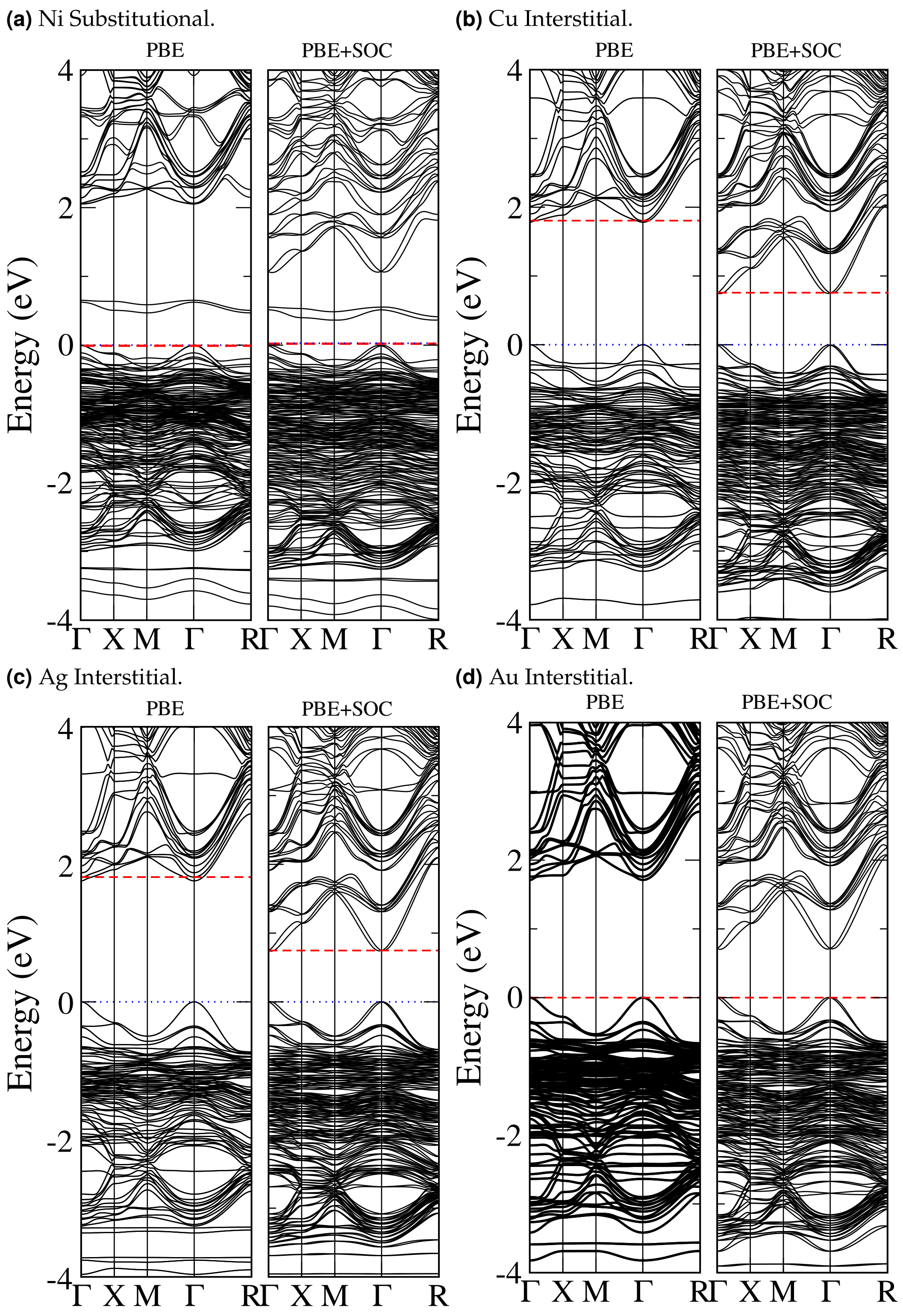

Figure S-19. Electronic band structures of four doped systems with Ni, Cu, Ag, and Au (panels a–d, respectively). The structures correspond to doping sites and charge states with the lowest formation energies E_F. Each panel contrasts results obtained at the PBE and PBE+SOC levels. The plots span the energy range from −4 to 4 eV. The dashed red line indicates the Fermi level, while the dashed blue line marks the zero of energy set at the valence band maximum.

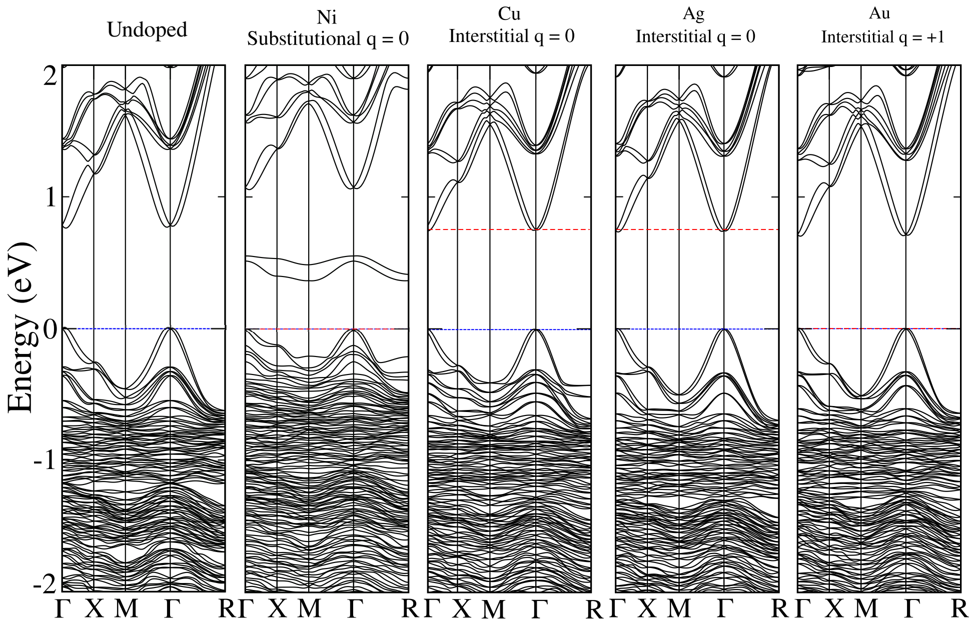

Figure S-20. Electronic band structures of pristine $MAPbI_3$ and doped structures with Ni, Cu, Ag, and Au. The plots span the energy range from −2 to 2 eV and correspond to doping sites and charge states with the lowest formation energies E_F. Calculations were performed at the PBE+SOC level. The dashed red line indicates the Fermi level, while the dashed blue line marks the zero of energy set at the valence band maximum.

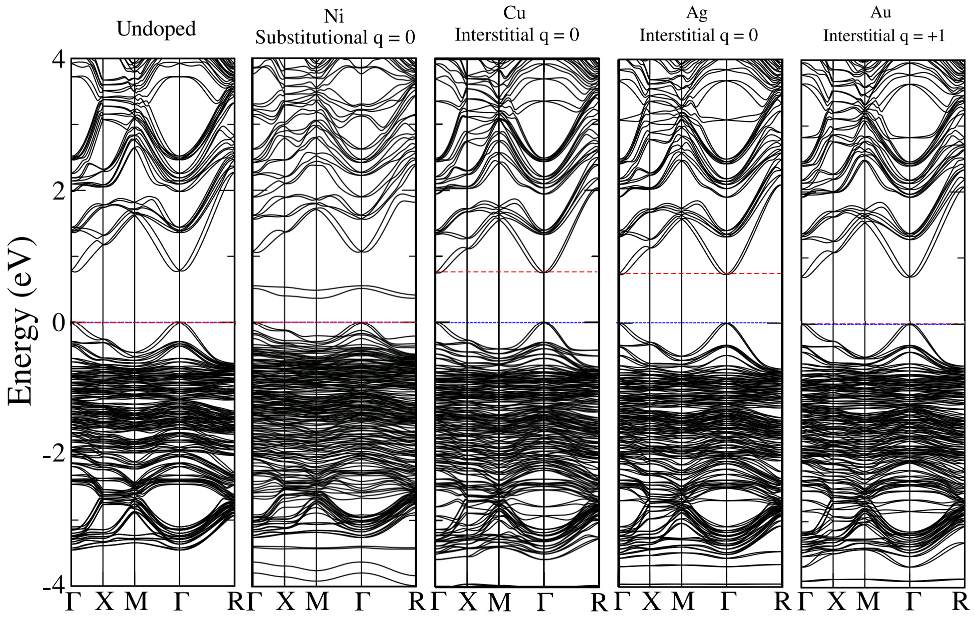

Figure S-21. Electronic band structures of pristine $MAPbI_3$ and doped structures with Ni, Cu, Ag, and Au. The plots span the energy range from −4 to 4 eV and correspond to doping sites and charge states with the lowest formation energies E_F. Calculations were performed at the PBE+SOC level. The dashed red line indicates the Fermi level, while the dashed blue line marks the zero of energy set at the valence band maximum.

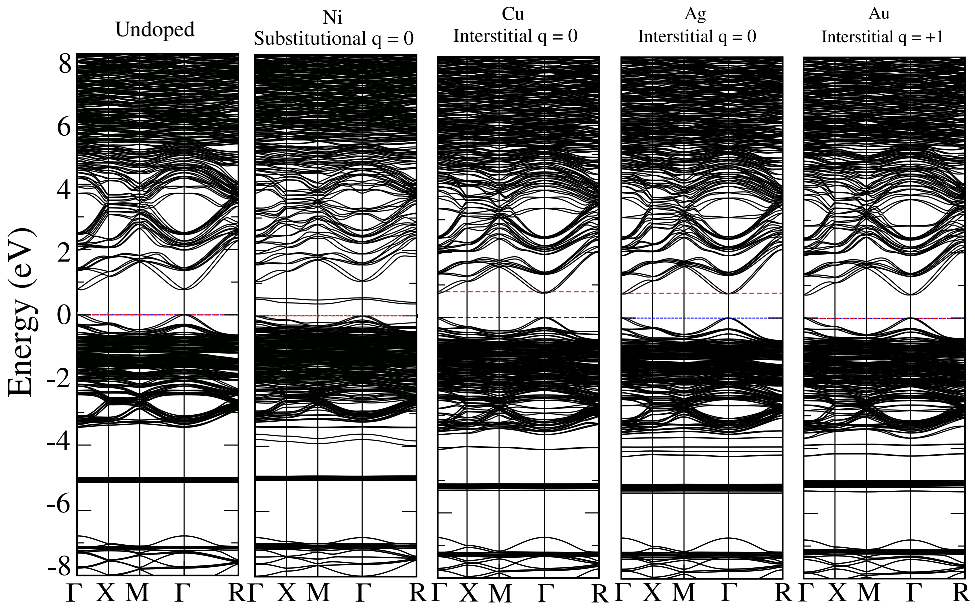

Figure S-22. Electronic band structures of pristine $MAPbI_3$ and doped structures with Ni, Cu, Ag, and Au. The plots span the energy range from −8 to 8 eV and correspond to doping sites and charge states with the lowest formation energies E_F. Calculations were performed at the PBE+SOC level. The dashed red line indicates the Fermi level, while the dashed blue line marks the zero of energy set at the valence band maximum.

S-7 ADDITIONAL DETAILS: EXPERIMENTAL PROCEDURE

1. **Substrate preparation**

   Fluorine-doped tin oxide (FTO) coated glasses substrates (TEC7 Glass Plates, ~7 Ω/sq), with dimensions of 2.5 cm × 1.5 cm were etched using Zn powder and HCl (4.0 mol/L) solution to create the desired pattern. The FTO surface was masked with polyimide (Kapton®) tape to prevent unwanted corrosion. The substrates underwent a sequential cleaning process in an ultrasonic bath, starting with an aqueous Hellmanex®(2 % v/v) solution for 25 min, followed by sequential rinsing with deionized water, acetone, and isopropanol (IPA) for 15 min each. After drying under a nitrogen gas flow, the substrates were submitted to UV-ozone treatment for 30 min at room temperature to eliminate organic residues and enhance surface properties.

2. **Preparation of $MAPbI_3$ films**

The $MAPbI_3$ perovskite layer (~300 nm thickness) was prepared using a one-step deposition method. Initially, the perovskite precursor solution was prepared by dissolving 645 mg of PbI_2 and 222 mg of MAI in 1 mL of a solvent mixture composed of DMF and DMSO in a 4:1 v/v ratio. An aliquot of this solution (60 µL) was deposited onto the substrate via spin-coating, starting at 1000 rpm for 10 s (ramp rate: 200 rpm/s), followed at 6000 rpm for 20 s (ramp rate: 2000 rpm/s). During the final 10 s of the spin-coating process, 200 µL of ethyl acetate was introduced as an antisolvent to facilitate rapid crystallization. Finally, the $MAPbI_3$ films were thermally annealed at 100 °C for 30 min.

3. **Deposition of the Metal Layer**

All metal layers were deposited in a 13.56 MHz RF Leybold-Heraus sputtering system, model LH-Z400, using three-inch diameter pure (> 99.99%) metal targets, separated by 5 cm from the anode. The base pressure in the deposition chamber was 3×10^{-6} mbar. All depositions were timed to approximately 40 Å total thickness from a known deposition rate for each metal target. For a given metal layer, all perovskite-metal samples were deposited simultaneously and taken for analysis or aging.

4. **XPS analysis**

XPS measurements were carried out on a Thermo Alpha110 hemispherical analyzer using a non-monochromatic Al-Kα anode as an excitation source. All measurements were taken with zero takeoff angle with respect to the surface normal using a low beam intensity. The XPS measurements were made using a 25 mm^2 square spot size in the middle of the samples. The pressure in the analysis chamber was $<1 \times 10^{-8}$ mbar during all measurements. All data treatment was done in the CasaXPS software, which was also used to remove Al-Kα satellites from the non-monochromatic X-ray source to correctly determine small peak areas that overlapped with satellites from stronger emissions. All samples were sat in a high to ultra-high vacuum for a few hours before measurements, waiting

for the chamber pressure to be sufficiently low. Quantification was performed on survey scans taken at 100 eV pass energy. Relative sensitivity factors used for quantification were based on Scofield Cross sections. Atomic percentages (at%) presented were calculated from quantification containing all elements but carbon in the sample, which was deemed mostly as an adventitious carbon contamination. Perovskite degradation due to X-ray beam damage was tracked during measurements and was seen to cause a minor formation of metallic lead.

5. **I-V Analysis**

   The devices based on FTO / $MAPbI_3$ / metal stacks (Ag, Au, Cu, or Ni) with an approximate metal thickness of 80 nm were fabricated to investigate the charge transport properties of $MAPbI_3$ films in contact with these metals. Current-voltage (I-V) curves were performed using a Keithley 2410-C source meter to analyze the electrical behavior of the interfaces. The active area of each device was 0.25 cm^2.

6. **UV-vis Absorption Spectroscopy**

   UV-vis absorption spectra of both freshly prepared and aged FTO / $MAPbI_3$ / metal stacks, containing approximately 4 nm of Ag, Au, Cu, and Ni, were acquired using an Agilent Cary UV-Vis spectrophotometer. A clean FTO substrate was employed as the baseline for all acquisitions.

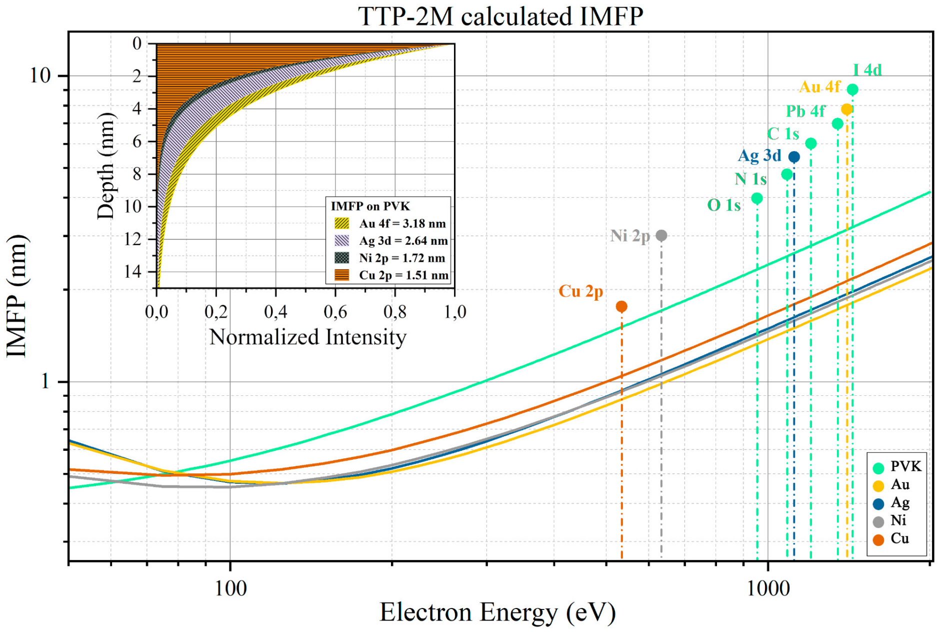

Figure S-23. Inelastic Mean Free Path (IMFP) for each material calculated using the NIST Electron Inelastic Mean Free Path Database (Version 1.2) software using the TTP-2M predictive formula with a nominal density of 4 g/cm^3 and a band gap of 1.55 eV for the $MAPbI_3$ perovskite.[26] Normalized Intensity as a function of depth of origin for photoelectrons emitted from the metal atoms core shell across the perovskite layer (inset). For a given element, the electron energy is the kinetic energy of the emitted photoelectron when excited by the 1486.6 eV Al-Kα radiation. The curves represent the IMFP of electrons across the metals or the perovskite layer, the vertical dotted lines marks the energy corresponding to each corresponding atom core shell photoelectron used for quantification.

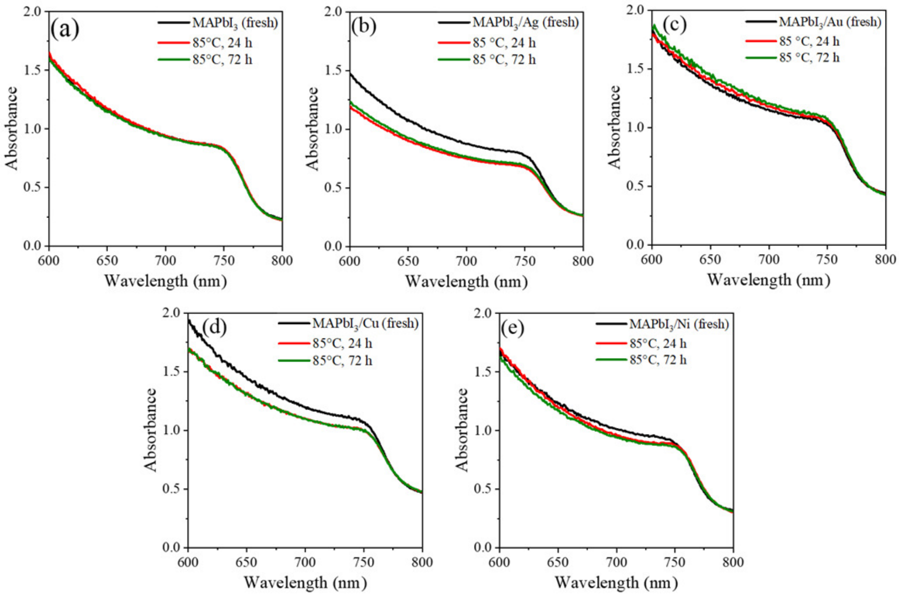

Figure S-24. UV–vis absorption spectra of freshly prepared and aged FTO / $MAPbI_3$ / metal stacks. Panel (a) presents the FTO / $MAPbI_3$ sample without a metal overlayer (fresh and aged reference), whereas panels (b–e) correspond to stacks incorporating ~4 nm of (b) Ag, (c) Au, (d) Cu, and (e) Ni. A clean FTO substrate was used as the baseline for all acquisitions.

Table S-16. Optical band gaps estimated from the absorption onset of the spectra shown in Figure S-23.

Sample	Aging time (h)	Band gap energy (eV)
FTO/$MAPbI_3$	0	1.56
	24	1.55
	72	1.56
FTO/$MAPbI_3$/Ag	0	1.55
	24	1.55
	72	1.55
FTO/$MAPbI_3$/Au	0	1.54
	24	1.54
	72	1.54
FTO/$MAPbI_3$/Cu	0	1.54
	24	1.52
	72	1.53
FTO/$MAPbI_3$/Ni	0	1.55
	24	1.54
	72	1.55

References

1 Kojima, A.; Teshima, K.; Shirai, Y.; Miyasaka, T. Organometal Halide Perovskites as Visible-Light Sensitizers for Photovoltaic Cells. *J. Am. Chem. Soc.* **2009**, *131*, 6050–6051, DOI: 10.1021/ja809598r.

2 Islam, J.; Hossain, A. K. M. A. Correction: Narrowing band gap and enhanced visible-light absorption of metal-doped non-toxic $CsSnCl_3$ metal halides for potential optoelectronic applications. *RSC Adv.* **2020**, *10*, 17869–17869, DOI: 10.1039/D0RA90054K.

3 Correa-Baena, J.-P.; Saliba, M.; Buonassisi, T.; Grätzel, M.; Abate, A.; Tress, W.; Hagfeldt, A. Promises and challenges of perovskite solar cells. *Science* **2017**, *358*, 739–744, DOI: 10.1126/science.aam6323.

4 Srikanth, M.; Ozório, M. S.; Da Silva, J. L. F. Optical and dielectric properties of lead perovskite and iodoplumbate complexes: an ab initio study. *Phys. Chem. Chem. Phys.* **2020**, *22*, 18423–18434, DOI: 10.1039/D0CP03512B.

5 Zhang, X.; Turiansky, M. E.; Shen, J.-X.; Van de Walle, C. G. Iodine interstitials as a cause of nonradiative recombination in hybrid perovskites. *Phys. Rev. B* **2020**, *101*, 140101, DOI: 10.1103/PhysRevB.101.140101.

6 Baikie, T.; Fang, Y.; Kadro, J. M.; Schreyer, M.; Wei, F.; Mhaisalkar, S. G.; Graetzel, M.; White, T. J. Synthesis and crystal chemistry of the hybrid perovskite $(CH_3NH_3)PbI_3$ for solid-state sensitised solar cell applications. *J. Mater. Chem. A* **2013**, *1*, 5628–5641, DOI: 10.1039/C3TA10518K.

7 Xue, H.; Brocks, G.; Tao, S. First-principles calculations of defects in metal halide perovskites: A performance comparison of density functionals. *Phys. Rev. Mater.* **2021**, *5*, 125408, DOI: 10.1103/PhysRevMaterials.5.125408.

8 Kerner, R. A.; Cohen, A. V.; Xu, Z.; Kirmani, A. R.; Park, S. Y.; Harvey, S. P.; Murphy, J. P.; Cawthorn, R. C.; Giebink, N. C.; Luther, J. M.; Zhu, K.; Berry, J. J.;

Kronik, L.; Rand, B. P. Electrochemical Doping of Halide Perovskites by Noble Metal Interstitial Cations. *Adv. Mater.* **2023**, *35*, 2302206, DOI: 10.1002/adma.202302206.

9 Stoumpos, C. C.; Malliakas, C. D.; Kanatzidis, M. G. Semiconducting Tin and Lead Iodide Perovskites with Organic Cations: Phase Transitions, High Mobilities, and Near-Infrared Photoluminescent Properties. *Inor. Chem.* **2013**, *52*, 9019–9038, DOI: 10.1021/ic401215x.

10 Leguy, A. M. A.; Hu, Y.; Campoy-Quiles, M.; Alonso, M. I.; Weber, O. J.; Azarhoosh, P.; van Schilfgaarde, M.; Weller, M. T.; Bein, T.; Nelson, J.; Docampo, P.; Barnes, P. R. F. Reversible Hydration of $CH_3NH_3PbI_3$ in Films, Single Crystals, and Solar Cells. *Chem. Mater.* **2015**, *27*, 3397–3407, DOI: 10.1021/acs.chemmater.5b00660.

11 Juarez-Perez, E. J.; Hawash, Z.; Raga, S. R.; Ono, L. K.; Qi, Y. Thermal degradation of $CH_3NH_3PbI_3$ perovskite into NH_3 and CH_3I gases observed by coupled thermogravimetry–mass spectrometry analysis. *Energy Environ. Sci.* **2016**, *9*, 3406–3410, DOI: 10.1039/C6EE02016J.

12 Kato, Y.; Ono, L. K.; Lee, M. V.; Wang, S.; Raga, S. R.; Qi, Y. Silver Iodide Formation in Methyl Ammonium Lead Iodide Perovskite Solar Cells with Silver Top Electrodes. *Adv. Mater. Interfaces* **2015**, *2*, DOI: 10.1002/admi.201500195.

13 Bhatt, P.; Kumar, A.; Singh, N.; Garg, A.; Nalwa, K. S.; Tewari, A. Long-Range Binding of Defect Clusters Leads to Suppressed Ion Mobility in Cs-Doped Methylammonium Lead Iodide. *ACS Appl. Energy Mater.* **2023**, *6*, 6615–6623, DOI: 10.1021/acsaem.3c00659.

14 Motti, S. G.; Meggiolaro, D.; Martani, S.; Sorrentino, R.; Barker, A. J.; Angelis, F. D.; Petrozza, A. Defect Activity in Lead Halide Perovskites. *Adv. Mater.* **2019**, *31*, DOI: 10.1002/adma.201901183.

15 da Silva Filho, J. M. C.; Gonçalves, A. D.; Marques, F. C.; de Freitas, J. N. A Review on the Development of Metal Grids for the Upscaling of Perovskite Solar Cells and Modules. *Solar RRL* **2021**, *6*, DOI: 10.1002/solr.202100865.

16 Abdi-Jalebi, M.; Pazoki, M.; Philippe, B.; Dar, M. I.; Alsari, M.; Sadhanala, A.; Divitini, G.; Imani, R.; Lilliu, S.; Kullgren, J.; Rensmo, H.; Grätzel, M.; Friend, R. H. Dedoping of Lead Halide Perovskites Incorporating Monovalent Cations. *ACS Nano* **2018**, *12*, 7301–7311, DOI: 10.1021/acsnano.8b03586.

17 Saliba, M.; Matsui, T.; Domanski, K.; Seo, J.-Y.; Ummadisingu, A.; Zakeeruddin, S. M.; Correa-Baena, J.-P.; Tress, W. R.; Abate, A.; Hagfeldt, A.; Grätzel, M. Incorporation of rubidium cations into perovskite solar cells improves photovoltaic performance. *Science* **2016**, *354*, 206–209, DOI: 10.1126/science.aah5557.

18 Abdelhady, A. L.; Saidaminov, M. I.; Murali, B.; Adinolfi, V.; Voznyy, O.; Katsiev, K.; Alarousu, E.; Comin, R.; Dursun, I.; Sinatra, L.; Sargent, E. H.; Mohammed, O. F.; Bakr, O. M. Heterovalent Dopant Incorporation for Bandgap and Type Engineering of Perovskite Crystals. *J. Phys. Chem. Lett.* **2016**, *7*, 295–301, DOI: 10.1021/acs.jpclett.5b02681.

19 Ma, X.; Yang, L.; Lei, K.; Zheng, S.; Chen, C.; Song, H. Doping in inorganic perovskite for photovoltaic application. *Nano Energy* **2020**, *78*, 105354, DOI: 10.1016/j.nanoen.2020.105354.

20 Lyons, J. L.; Swift, M. W. Trends for Acceptor Dopants in Lead Halide Perovskites. *J. Phys. Chem. C* **2023**, *127*, 12735–12740, DOI: 10.1021/acs.jpcc.3c01972.

21 Soopy, A. K. K.; Parida, B.; Aravindh, S. A.; O. Al Ghaithi, A.; Qamhieh, N.; Amrane, N.; Benkraouda, M.; Liu, S. F.; Najar, A. Towards High Performance: Solution-Processed Perovskite Solar Cells with Cu-Doped $CH_3NH_3PbI_3$. *Nanomaterials* **2024**, *14*, DOI: 10.3390/nano14020172.

22 Suzuki, A.; Oe, M.; Oku, T. Fabrication and Characterization of Ni-, Co-, and Rb-Incorporated $CH_3NH_3PbI_3$ Perovskite Solar Cells. *J. Electron. Mater.* **2021**, *50*, 1980–1995, DOI: 10.1007/s11664-021-08759-1.

23 Liu, W.; Feng, Y.; Li, L.; Ma, Y.; Hu, R.; Wu, X.; Chu, L.; Li, X.; Huang, W. Stable and Efficient Pb–Ni Binary Metal Perovskite Solar Cells. *ACS Sustainable Chem. Eng.* **2021**, *9*, 17112–17119, DOI: `10.1021/acssuschemeng.1c06322`.

24 Dias, A. C.; Lima, M. P.; Da Silva, J. L. F. Role of Structural Phases and Octahedra Distortions in the Optoelectronic and Excitonic Properties of $CsGeX_3$ (X = Cl, Br, I) Perovskites. *J. Phys. Chem. C* **2021**, *125*, 19142–19155, DOI: `10.1021/acs.jpcc.1c05245`.

25 Chagas, L. G.; Da Silva, J. L. F.; Lima, M. P. Role of Jahn-Teller distortion in the relative stability between the black and yellow phases of transition metal doped $CsSnI_3$ perovskites. *Phys. Rev. B* **2024**, *109*, 014106, DOI: `10.1103/PhysRevB.109.014106`.

26 Powell, C. J.; Jablonski, A. Progress in Quantitative Surface Analysis by X-ray Photoelectron Spectroscopy: Current Status and Perspectives. *J. Electron Spectrosc. Relat. Phenom.* **2010**, *178–179*, 331–346, DOI: `10.1016/j.elspec.2009.05.004`.
